# Specialized Yeast Ribosomes: A Customized Tool for Selective mRNA Translation

**DOI:** 10.1371/journal.pone.0067609

**Published:** 2013-07-08

**Authors:** Johann W. Bauer, Clemens Brandl, Olaf Haubenreisser, Bjoern Wimmer, Manuela Weber, Thomas Karl, Alfred Klausegger, Michael Breitenbach, Helmut Hintner, Tobias von der Haar, Mick F. Tuite, Lore Breitenbach-Koller

**Affiliations:** 1 Department of Cell Biology, University of Salzburg, Salzburg, Austria; 2 Department of Dermatology, General Hospital Salzburg/PMU, Salzburg, Austria; 3 Kent Fungal Group, School of Biosciences, University of Kent, Canterbury, Kent, United Kingdom; The John Curtin School of Medical Research, Australia

## Abstract

Evidence is now accumulating that sub-populations of ribosomes - so-called specialized ribosomes - can favour the translation of subsets of mRNAs. Here we use a large collection of diploid yeast strains, each deficient in one or other copy of the set of ribosomal protein (RP) genes, to generate eukaryotic cells carrying distinct populations of altered ‘specialized’ ribosomes. We show by comparative protein synthesis assays that different heterologous mRNA reporters based on luciferase are preferentially translated by distinct populations of specialized ribosomes. These mRNAs include reporters carrying premature termination codons (PTC) thus allowing us to identify specialized ribosomes that alter the efficiency of translation termination leading to enhanced synthesis of the wild-type protein. This finding suggests that these strains can be used to identify novel therapeutic targets in the ribosome. To explore this further we examined the translation of the mRNA encoding the extracellular matrix protein laminin β3 (LAMB3) since a LAMB3-PTC mutant is implicated in the blistering skin disease Epidermolysis bullosa (EB). This screen identified specialized ribosomes with reduced levels of RP L35B as showing enhanced synthesis of full-length LAMB3 in cells expressing the LAMB3-PTC mutant. Importantly, the RP L35B sub-population of specialized ribosomes leave both translation of a reporter luciferase carrying a different PTC and bulk mRNA translation largely unaltered.

## Introduction

The ability to elevate the expression of a target protein without impacting on bulk mRNA translation is a key requirement of many current biotechnological processes and of certain biomedical intervention strategies. In the former case, the aim is to elevate the levels of recombinant protein expression to commercially feasible levels without harming the cellular machinery that ensures the protein is correctly folded and delivered in an authentic form. In the latter case, a wide range of human diseases can be treated by the directed suppression or enhancement of the levels of key disease-related proteins.

Presently, there are two major routes to modulating gene expression at the post-transcriptional level. One involves the manipulation of either the 5′ and 3′ non-coding regulatory sequences [1], [2] or the coding sequences [3], [4] of a target mRNA to be expressed endogenously or in a heterologous host. The second requires the application of chemical interventions such as antibiotics to inhibit pathogen, but not host-directed protein synthesis [5]. These two approaches are based on time-consuming, technological developments and are difficult to direct towards specific mRNA targets.

Recently, the ribosome has emerged as a potential target for delivering the desired, directed mRNA translation. The translating ribosome is assembled from two unequal subunits, each composed of ribosomal RNA (rRNA) and ribosomal proteins (RPs). The prokaryotic 70S ribosome consists of a 50S large subunit (LSU) and a 30S small subunit (SSU) [6], [7] while the more complex eukaryotic ribosome consists of 60S and 40S subunits [8]. The eukaryotic and prokaryotic LSU and SSU are primed for mRNA translation by complex yet distinct translation initiation processes which generate the translationally competent ribosome. For several decades, it has been thought that during this process, all ribosomes serve as static translation platforms, receiving regulatory input from both general and mRNA-specific translation factors [9]. However, several lines of evidence now point to the possibility that sub-populations of ribosomes - specialized ribosomes - with intrinsically altered translational activity may actually exist in a cell and that these favour altered translation of a subpopulation of mRNAs.

The first evidence that substrate-specific sub-populations of ribosomes could exist emerged from the development of orthogonal ribosomes in prokaryotes [10] which are artificially engineered ribosomes that are able to operate alongside, but independently of, the endogenous ribosome pool. Such orthogonal ribosomes have served as a test bed to establish how variations in ribosomal RNA or specific RPs can enhance the incorporation of the rare amino acid selenocysteine [11], and to introduce unnatural amino acids into proteins via evolution of a quadruplet-decoding ribosome [12]. They can also be used to perform structure-function studies of ribosomes [13].

These findings have triggered renewed interest in reports on the existence of specialized ribosomes in nature. For example, the prokaryotic ribosome under conditions where translation of bulk mRNA ceases, can initiate translation on leaderless mRNAs and translate them [14]. Functional studies employing the aminoglycoside kasugamycin, which inhibits general initiation of translation in bacteria, revealed that the drug induced loss of several RPs which then allowed for the structural changes in rRNA that were necessary for the specialized translation of leaderless mRNAs [15]. Further evidence that specialized ribosomes can be generated by structural rearrangements of the canonical ribosome has also recently emerged [16]. These authors identified the stress-induced endoribonuclease MazF, which removes a fragment of rRNA at the ribosomal decoding centre thereby generating a sub-population of ribosomes that can now selectively translate leaderless mRNAs both *in vivo* and *in vitro*.

The ability to generate specialized ribosomes through the partial loss of rRNA sequences or RPs is not unique to prokaryotes. For example, the yeast *Saccharomyces cerevisiae* ribosome serves as a prototypic model for exploiting natural strategies to engineer specialized ribosomes [17], [18]. The yeast ribosome consists of a LSU made up of 25S, 5.8S and 5S rRNA and 46 RPs (RPs), and the SSU which contains the 18S rRNA and 32 RPs. Through an ancient genome duplication [19] 59 of the 78 yeast RPs are encoded by duplicated genes [20], [21]. Furthermore, the extensive modification of rRNA and RPs is thought to assist ribosome biogenesis and structural integrity, respectively [22], [23]. Currently, evidence is accumulating that these modifications may also serve a regulatory role in mRNA translation [24]. In addition, during evolution, the rRNAs have gained many extension segments, which may serve as instruments for specialized mRNA translation [25], while the most conserved sequence tracts are typically found in the core functional regions of the ribosome i.e. the A site of the SSU, where the triplets are decoded by the cognate aminoacyl-tRNAs (aa-tRNAs); the P-site of the LSU, where peptide bond formation is catalysed; and the E-site, where the decharged tRNAs leave the ribosome.

Even the most highly conserved rRNA tracts may be functionally altered as cryo-electron microscopy and X-ray analysis of yeast ribosomes have shown that most RPs with long protrusions from the surface of the ribosome can reach down to the core rRNA functional regions. This makes RPs interesting candidates for functional modulation of rRNA tracts [26], [27]. This observation, coupled with the fact that RPs with respect to number, sequence and position on the ribosome are highly conserved from yeast to man, makes RPs highly attractive candidates for studies on regulation of general and specialized mRNA translation in eukaryotes [28].

A new approach to the rational engineering of the efficiency of translation of specific mRNAs has recently emerged from a study of RP deficiencies in yeast that employed strains carrying single deletions in the 59 duplicated RP genes [17], [29], [30]. This revealed RP paralog-specific requirements for translation of selected mRNAs and led to the authors proposing a ‘ribosomal code‘ whereby subsets of mRNAs might be translated by compositionally distinct ‘specialized’ ribosomes [17]. Importantly, reducing, but not eliminating availability of both single copy and duplicated RP genes in yeast does not necessarily impair overall ribosome function and viability although it can lead to distinct phenotypes [31], [32]. Notably, ribosomal protein-mediated control of mRNA translation is also found in the mouse where a deficiency in the RP RpL38 generates a pool of “specialized ribosomes” that specifically affect the translation of a distinct subset of homeobox mRNAs during mouse development [33].

One unifying concept emerging from these reports of specialized ribosomes is that both artificially engineered ribosomes and the exploitation of naturally-occurring ribosome variants must be monitored and adapted to direct mRNA-specific translation, yet which does not compromise endogenous bulk translation that may lead to cell death [34], [35]. One such approach is presented here, where we employ a collection of 124 diploid yeast strains in which one copy of one or the other of the yeast RP gene pairs has been deleted from the genome. Thus we are able to test single copy and duplicate RP genes as well as being able to assess possible differences exerted by reduction in either one of the paralogous RP genes. For each strain we evaluated the translation of two pairs of reporter mRNA based on Firefly (FF) luciferase with one member of each pair carrying a premature termination codon (PTC) mutation. In one pair the FF was fused in-frame to a human LAMB3 cDNA encoding the extracellular matrix protein laminin β3 either without or with a PTC. A PTC mutant of LAMB3 has been implicated in the blistering skin disease Epidermolysis bullosa (EB). In addition all strains were engineered to co-express a wild type Renilla (REN) luciferase gene to provide an internal control. Using these reporters we describe the systematic screening of yeast cells, carrying individually altered ribosomes, for their ability to modulate the translation of individual luciferase-based reporter mRNAs. By so doing, we identify cells with specialized ribosomes and describe how the results of such a screen may be exploited in biotechnology, for directing increase in the translation of a specific heterologous mRNA, or how it can be used to guide the search for RP targets that can serve as a novel therapeutic target to induce specialized mRNA translation of a defective human disease gene.

## Materials and Methods

### Yeast Strains, *E. coli* Strains, Growth Media

In the yeast *S. cerevisiae*, the 78 RPs are encoded by 137 genes that include 19 single copy RP genes and 59 duplicated RP genes. 124 different diploid strains, heterozygous for deletions of one or other of most of the RP genes and viable under the culture conditions used in this study were obtained from the EUROSCARF gene deletion collection (http://web.uni-frankfurt.de/fb15/mikro/euroscarf) [36]. The remaining 13 strains could not be revived from long-term storage. Yeast and *E. coli* media, culture conditions and the manipulation of yeast and *E. coli* strains were as described previously [37]. The employed RP deletion strains (**Table S1**) were grown on yeast extract - peptone - dextrose (YPD) medium or defined YNB-based medium (SC) to provide the desired selective conditions.

### Design and Cloning of Reporters

**Figure 1:**
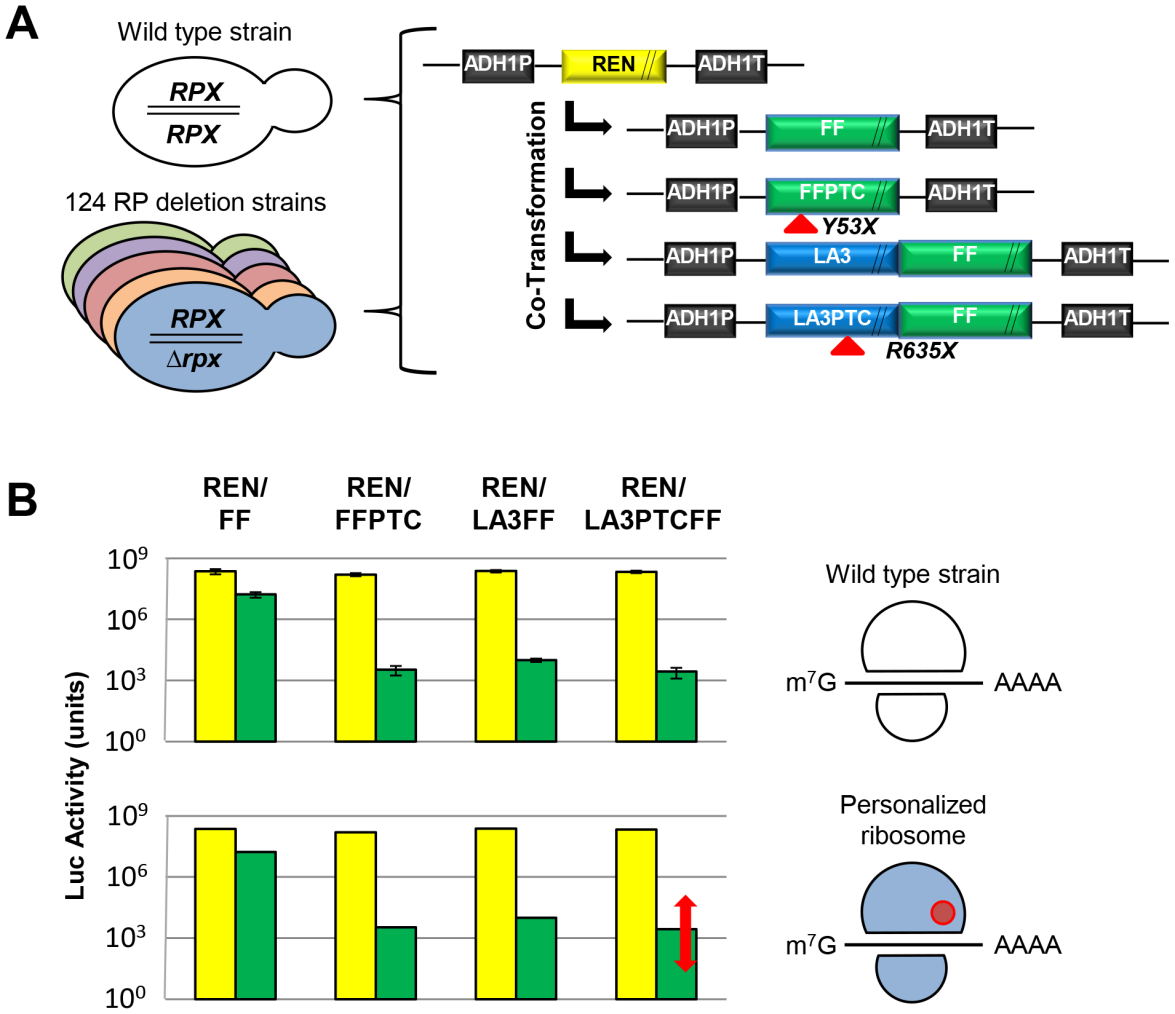
Yeast variant ribosome screen. (**A**) Five individual luciferase reporters were generated, all sharing identical promoter (ADH1P) and terminator (ADH1T) sequences. PTC codons (X) are depicted by a red arrow and the length of the coding sequence is indicated in base pairs. Co-transformed reporter pairs are indicated by the black arrows. NB: Not to scale. (**B**) Luciferase activity (relative light units) reports expression level of REN (yellow) and FF (green) reporters for the REN/FF, REN/FFPTC, REN/LA3FF and REN/LA3PTCFF reporter pairs. A possible change in reporter expression level between wild type strain and one of the 124 RP deletion strains (*RPX/Δrpx*) is reported by an altered luciferase readout (red arrow). This allows the identification of specialized ribosomes for selected modulation of a target mRNA; in this example, the LA3PTCFF mRNA.

Parent vectors used were the yeast centromeric plasmids YCplac33 (*URA3*) and YCplac111 (*LEU2*) [38]. The luciferase reporters (**Figure 1**) were cloned in a modular fashion. Parent vector YCplac33 was manipulated to harbor the firefly luciferase (FF) reporter gene under control of the yeast *ADH1* promoter and terminator sequences, and the resulting plasmid designated pLM162. This plasmid was then manipulated to harbor the LAMB3 FF fusion reporter (LA3FF) under the control of the yeast *ADH1* promoter and terminator sequences to generate the plasmid pLM168.

YCplac111 was manipulated to harbor the REN luciferase reporter gene under control of the yeast *ADH1* promoter and terminator sequences, respectively, and designated pLM164. The *ADH1* promoter was PCR amplified from pGBKT7 (Clontech, Mountain View, CA, USA) with a forward primer (5′ GGGAC*AAGCTT*ATCCTTTTGTTGTTTCCGGG 3′; *Hin*dIII restriction site shown in italics) and a reverse primer (5′ GGGAC*CTGCAG*GATAGACATTGTATATGAGATAGTTGATTGTATGCTTGGTA 3′; *Pst*I restriction site in italics) and cloned into YCplac33 via *Hin*dIII and *Pst*I restriction sites, restoring the wild type sequence of *ADH1* promoter and resulting in the plasmid pLM160. The *ADH1* terminator was amplified from pGBKT7 with a forward primer (GGGAC*GAGCTC*GCCGCATAACTAGCATAAC (*Sac*I restriction site in italics) and reverse primer (5′ GGGAC*GAATTC*AAGCGTGCGTGCCGGTAGAGGTGTGGTCA 3′; *Eco*RI restriction site in italics), and cloned into plasmid pLM160 via *Sac*I/*Eco*RI yielding plasmid pLM161.

The FF luciferase reporter was amplified from plasmid pGL4.10 (Promega Inc., Madison, WI, USA) with a forward primer (5′ GGGAC*GGTACC*GAAGATGCCAAAAACATTAAGAAGG 3′; *Kpn*I restriction site displayed in italics) and a reverse primer (5′ GGGAC*GAGCTC*TTACACGGCGATCTTGCCG 3′; *Sac*I restriction site in italics) and cloned into vector pLM161 via *Kpn*I/*Sac*I, generating the reporter plasmid pLM162. The start and the stop codons of the FF ORF were excluded so that the FF reporter harbors an ATG start codon flanked by 5′ *ADH1* sequences and a stop codon followed directly by *ADH1* 3′ sequences.

In an analogous fashion, the REN luciferase reporter was PCR amplified from plasmid pGL4.75 (Promega Inc) with a forward primer (5′ GGGAC*TCTAGA*CTTCCAAGGTGTACGAC 3′; *Xba*I restriction site in italics) and a reverse primer (5′ GGGAC*GAGCTC*TTACTGCTCGTTCTTCAGCA 3′; *Sac*I site in italics) and cloned into plasmid pLM161 via *Xba*I and *Sac*I, generating plasmid pLM163. Finally, the REN reporter plasmid pLM164 was constructed by sub-cloning the *ADH1* promoter-REN-*ADH1* terminator cassette from pLM163 to YCplac111 via *Hin*dIII/*Eco*RI restriction sites.

The LA3FF fusion reporter was cloned using a similar strategy. The human LAMB3 coding sequence was PCR amplified from a human cDNA library with a forward primer (5′ GGGAC*TCTAGA*AGACCATTCTTCCTCTTGTGTTTTGCCCTG 3′; *Xba*I restriction site in italics) and a reverse primer (5′ GGGAC*GGTACC*CTTGCAGGTGGCATAGTAGAGCACG 3′; *Kpn*I restriction site in italics). LAMB3 was cloned upstream in frame of the firefly luciferase via *Xba*I/*Kpn*I restriction enzyme sites into vector pLM162, thereby generating plasmid pLM168.

All constructs were verified by DNA sequencing employing the BigDye® Terminator v3.1 Cycle Sequencing Kit (Applied Biosystems, Forster City, CA, USA) with an ABI 3130 Genetic Analyzer (Applied Biosystems, Forster City, CA, USA).

### Generation of Premature Termination Codon (PTC) Alleles

The generation of a premature termination codon (PTC) in the parent FF luciferase reporter plasmid pLM162 was performed using the Quikchange II XL Site-directed Mutagenesis kit from StrataGene (La Jolly, CA, USA) according to the manufacturer’s protocol, using forward primer 5′-ATATCGAGGTGGACATTACCTAAGCCGAGTACTTC-3′ and reverse primer 5′-GAAGTACTCGGCTTAGGTAATGTCCACCTCGATAT-3′. This generates a C to A change in the tyrosine codon (UAC) at position 53, resulting in a premature UAA stop codon and thus generating plasmid pLM167 (FFPTC). Nucleotides changed by mutagenesis are underlined in the primer sequences.

In an analogous fashion the LAMB3-PTC fusion reporter was generated as described for the LA3FF reporter, using the mutated human LAMB3 coding sequence from a human cDNA library, harboring a PTC at codon R635, generating pLM169 (LA3PTCFF) [39].

### Luciferase Assay

For every experimental setup, the wild type strain and the individual RP EUROSCARF deletion strains were co-transformed with the REN reporter plasmid pLM161 and one or other of the four different FF reporters. This generated co-expressed REN/FF, REN/FFPTC, REN/LA3FF or REN/LA3PTCFF reporter pairs in each strain. The luciferase assays were performed with the Dual-Luciferase® Reporter Assay System and the reagents were prepared as indicated by the supplier (Promega Inc). Yeast cells were grown to exponential phase at 28°C in SC-ura-leu medium. After harvesting, cell concentration was determined using a CASY Model TT cell counter (Roche Diagnostics, Applied Sciences, Vienna, Austria) to analyse an equal amount of 1×10^7^ cells for each experiment. After centrifugation of the cell suspension, the resulting supernatant was removed and cells were lysed by dissolving in 1 ml of 1× passive lysis buffer (PLB) for 30 min at room temperature. After centrifugation to remove cell debris, 20 µl of the lysate were transferred into a black & white 96 multiwell isoplate (Perkin Elmer, Waltham, MA, USA) and luminescence measurements were performed with the Glomax Multi luminometer (Promega Inc., Madison, WI, USA). For the FF and the REN reactions 50 µl of LAR II and 50 µl Stop & Glo®, respectively, were automatically injected into the lysate. After a lag time of 2 sec upon substrate addition, luminescence signals were integrated for 10 sec. To be able to perform descriptive and interference statistical analysis, measurements were performed using two biological replicates each in triplicate to give a sum of six recordings for the respective REN and FF luminescence reporter pairs in every RP deletion strain.

The experimental conditions used were able to report luminescence signals within the linear range for both co-transformed FF and REN reporter activities (2×10^7^ and 1×10^8^ luminescence counts, respectively) and in addition, luminescence readouts for FFPTC, LA3FF and LA3PTCFF reporters were approximately 4×10^3^, 2×10^4^ and 3×10^3^, respectively. These latter levels of luciferase activity are above limit of detection and limit of quantification which, for the FF and REN reporter background signals, were approximately 5×10^1^ and 3×10^2^, respectively.

### Statistical Analysis

The individual reporter luciferase readouts were subjected to outlier statistics using the Shapiro-Wilks test (employing MS Excel), which is a thorough test for the normality of the obtained data. The descriptive statistics were performed employing Sigmastat 3.1 (**Table S2**). Multiple comparisons of the individual mean readouts of the respective reporter readouts against the grand mean were performed using an ANOVA test (Holm-Sidak method; P = 95%; alpha = 0.001) and are shown in **Table S3** and documented in **Tables S4–S11**. Values significantly different from the grand mean were then checked for being lower or higher (i.e. to find RP deletion strains which favour lower and higher expression levels of luciferase reporters) and these are documented in **Table S12**. Also, the data for every individual reporter signal was normalized to the grand mean of the respective reporter readouts in all ribosomal deletion strains and plotted as histograms. For the comparison of two different normalized data points for a given deletion strain (for example comparison of REN in the background of FF to REN in the background of FFPTC), the data were plotted into a two dimensional graph and the Pearson product moment was calculated using MS Excel.

### RT-PCR

A 50 ml yeast cell culture was grown in SC-ura-leu medium to an OD_600_ = 0.7–0.8 and cells were harvested by centrifugation (1000 g, 5 min, 4°C). RNA was extracted from these cells using the Qiagen RNeasy® Mini Kit (Qiagen, Hamburg, Germany) according to the manufacturer’s protocol and using Zymolyase (10 mg/ml) (Seikagaku Corporation, Tokyo, Japan) to remove cell walls. Efficient DNAse digestion during RNA isolation was achieved using the Qiagen RNAse-free DNAse Kit (Qiagen, Hamburg, Germany).

The following primers were used for monitoring the intactness of the LA3FF and LA3PTCFF mRNA coding sequences, respectively. The primers used for generating cDNAs are underlined within a given pair of primers, used for RT-PCR. Primer pair (1) LA3FF 5′ (LG873 GGGGGAGATCACAAACTTGA forward, LG874 GTGCTGGCAGACACAGACAT reverse), primer pair (2) LA3FF 3′ (LG 875 AAGATGTGGTTGGGAACCTG forward, LG876 ATCCGTGTCCAGAAGTCACC reverse), primer pair (3) FF 3′ (LG885 GGACTTGGACACCGGTAAGA forward, LG886 GAAGAAGTGCTCGTCCTCGT reverse), actin primer pair (LG881 ACATCGTTATGTCCGGTGGT forward, LG882 AGATGGACCACTTTCGTCGT reverse). Primer pair (4) REN 3′ (LG 879 TAGACGGCCTACCCTCTCCT forward, LG880 CATTTCATCTGGAGCGTCCT reverse) was used in the same fashion to evaluate the REN luciferase mRNA.

Generation of cDNAs was performed with the Thermo Scientific RevertAid™ H Minus First Strand cDNA Synthesis Kit (Thermo Scientific, Waltham, MA, USA). 1 µg of isolated RNA and 40 pmol of each primer LG874, LG876, LG886, LG882 and LG880 were diluted in a total volume of 19 µl with RNAse-free water, incubated at 68°C for 5 min and then cooled on ice for 5 min. 6 µl aliquots of a 5×MMLV reaction buffer (250 mM Tris-HCl (pH 8.3), 250 mM KCl, 20 mM MgCl_2_, 50 mM DTT), 2 µl dNTP (10 mM), 1 µl RNAse-free water, 1 µl RiboLock™ RNAse inhibitor (20 U/µl) and 1 µl MMLV reverse transcriptase (200 U/µl) were added and the resulting RNA/primer mix was incubated at 42°C for 80 min, followed by addition of 10 µl NaOH (0.1 M) and further incubation at 70°C for 10 min. Finally, for neutralization 10 µl HCl (0.1 M) was added to each reaction and the resulting cDNA was stored at −20°C prior to use.

RT-PCR was performed using a Rotorgene RG-3000 Real-time thermal cycler (Corbett Research, Mortlake, NSW, Australia) and the 2× GoTaq® qPCR Master Mix from Promega (Promega Inc). The cDNA stock was diluted 1∶10 and the RT-PCR master-mix was prepared by addition of 0.6 µl primer solution (10 pmol/µl) to 5 µl GoTaq® qPCR Master Mix. Finally 5 µl of diluted cDNA (1∶10) and 5 µl qPCR/primer mix were used to perform RT-PCR runs according to the following reaction conditions: hold (95°C, 4 min), then cycling (denaturation 95°C, 10 sec; annealing 61°C, 15 sec; elongation 72°C, 20 sec) for 50 cycles. Results were analyzed with Rotorgene 6 software (Corbett research, Mortlake, NSW, Australia), using a threshold value of 0.08 and MS Excel for calculation of Δct values and diagrams.

## Results and Discussion

This study employed a series of yeast strains based on the diploid BY4743, with each strain being heterozygous for a specific RPL (RP of the large subunit) or RPS (RP of the small subunit) gene deletion. Each strain would therefore be expected to have a sub-population of ‘specialized’ ribosomes as a consequence of reductions in RP gene dosage, but not to exhibit complete absence of a given RP in this strain. In this way, minimal interference with overall ribosome function can be achieved, comparable to the partial inactivation of a ribosomal protein in nature by post-translational modification [40]. This approach excludes the use of haploid RP gene deletions as these would generate ribosomes completely devoid of a given ribosomal protein. However, loss of non-essential single copy RPs as well as loss of one RP protein paralogue, may reduce cellular viability [41].

In each of the 124 strains we systematically quantified the translation of four different FF luciferase-based reporter mRNAs **(** **Figure 1** **)**. These reporters consisted of wild type FF, an FF derivative harboring a premature termination codon (FFPTC) at codon 53, a human laminin β3-FF fusion reporter (LA3FF) and a derivative of this fusion containing a premature termination codon at codon 635 in the LA3 sequence (LA3PTCFF). As an independent control, the levels of a wild type REN luciferase mRNA were also assayed in the same co-transformed cells. All REN and FF reporters were engineered to have identical 5′ and 3′ untranslated regions (UTRs), derived from the yeast *ADH1* mRNA.

Human laminin β3 (encoded by the LAMB3 gene) was chosen for this study because defects in the synthesis of this protein caused by premature termination codons (PTCs) have been associated with severe forms of the devastating blistering skin disease Epidermolysis bullosa (EB) that present with extremely sensitive skin and fragile epithelial lining of the gut and internal organs [39]. A number of other human genetic diseases that are due to PTCs (e.g. forms of Muscular Dystrophy and Cystic Fibrosis) have been treated with aminoglycoside antibiotics that stimulate translational readthrough of the PTC to produce a therapeutically beneficial level of the full length protein [42]. However, the severe side effects of these drugs on the kidney and ear mean that such antibiotic treatment is not well tolerated [43]. We were interested in exploring the potential of selective translation of LAMB3-PTC mRNA in order to generate full length LAMB3 protein via modification of ribosome composition as a potential route for treating EB and other genetic disease caused by PTCs.

To monitor the functionality of the various FF and REN luciferase reporters and to investigate any possible changes in their expression in the different specialized ribosome yeast strains, we first optimized and validated their luminescence readouts in wild type cells. To provide meaningful data sets for statistical analysis, two biological replicates each with three individual luciferase measurements were assayed individually for each reporter throughout the study (see Materials & Methods). The mean REN luminescence readout when co-expressed in the presence of each of the four FF-based reporters was approximately 2×10^8^ relative light units (RLU) for all four strains (**Table 1**). The mean luminescence readouts for the FF, FFPTC, LA3FF and LA3PTCFF reporters were 1.6×10^7^, 3.3×10^3^, 1×10^4^ and 2.6×10^3^ RLU respectively. Such a difference in luciferase signal strength between REN and FF reporters has been reported by others [44]. The relatively low levels of luciferase activity for the LA3FF reporter is a common feature of such large heterologous reporters in yeast (e.g. [45] but the levels nevertheless are significantly above background levels and are clearly detectable (**Table 1**). Importantly, the luciferase readouts document faithful execution of the PTC signal in the prototypic FF/FFPTC reporters. For the LA3FF/LA3PTCFF reporters, a smaller but nevertheless significant reduction of 75% in luciferase activity was observed. Endogenous readthrough of termination codons in yeast mRNAs can vary over a wide range and depend on the termination codon and its nucleotide context [46] as was observed for the two PTC reporters used here. Equivalent levels of readthrough of a termination codon have been reported in yeast with natural termination codons [47].

**Table 1:** Luciferase levels in the wild type parent strain co-transformed with the various reporter constructs used in this study.

Wild Type	Size	Outlier	Mean	Std Dev	Std Error	Max	Min	Median
**FF**	17	1	16086480,6	4724609,41	1145886,1	23804300	9526670	16007100
**REN[FF]**	17	1	218780647	59325599,4	14388571,3	332062000	152339000	212297000
**Ratio FF/REN[FF]**	17	1	0,073	0,00577	0,0014	0,08	0,0619	0,0754
**FFPTC**	18	0	3256,6	1719,964	405,399	7216,36	953,946	2956,2
**REN[FFPTC]**	18	0	156667167	28555052	6730490,31	212800000	121886000	151120000
**Ratio FFPTC/REN[FFPTC]**	18	0	0,00002	0,00000844	0,00000199	0,000035	0,00000769	0,0000189
**LA3FF**	18	0	10498,192	2550,23	601,095	16625,6	7133,19	10268,745
**REN[LA3FF]**	18	0	235116444	29864092,9	7039034,2	280782000	186504000	238958000
**Ratio LA3FF/REN[LA3FF]**	18	0	0,0000448	0,00000978	0,0000023	0,0000672	0,0000302	0,000043
**LA3PTCFF**	14	4	2605,625	1556,829	416,08	5313,34	577,717	2310,995
**REN[LA3PTCFF]**	14	4	203772857	21433332,4	5728299,05	246684000	176643000	199546000
**Ratio LA3PTCFF/REN[LA3PTCFF]**	14	4	0,0000128	0,00000795	0,00000213	0,0000288	0,00000293	0,0000113
**Background FF signal (Mean)**	50,266775	times 10	Limit of quantification		502,66775			
**Background REN signal (Mean)**	178,78515	times 10	Limit of quantification		1787,8515			

To exclude the possibility that the low LA3FF and LA3PTCFF protein expression levels resulted from the expression of fragmented mRNAs, we performed RT-PCR employing successive primer pairs covering the complete LA3FF and LA3PTCFF sequences (**Figure S1**). This revealed that both the LA3FF and LA3PTCFF mRNAs were present in wild type BY4743 cells as complete mRNAs and therefore the 75% reduction in LA3PTCFF protein levels compared to the LA3FF levels most likely reflects a post-transcriptional event i.e. termination of translation by the PTC signal and the subsequent failure to translate the downstream FF luciferase ORF.

**Figure 2:**
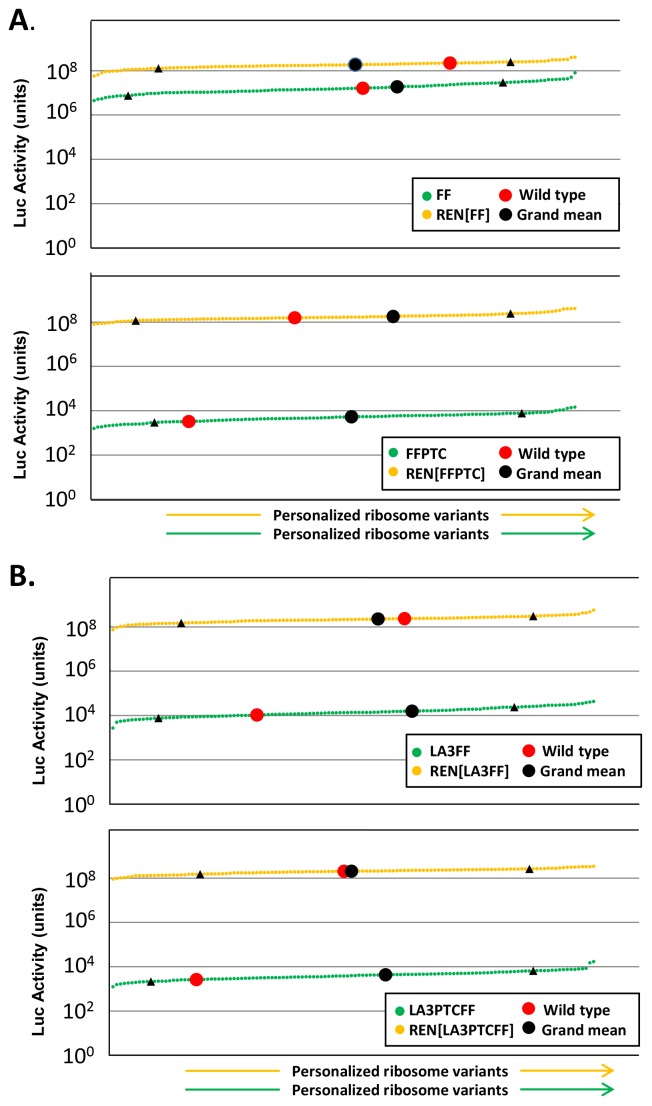
Luciferase expression profiles in 124 yeast strains each with a different individual specialized ribosome variant. The luciferase readouts of each of the four individual FF-based reporters, co-expressed with the REN reporter, are shown as follows: (**A**) FF/REN and FFPTC/REN; (**B**) LA3FF/REN and LA3PTCFF/REN. The luciferase readouts were monitored in all 124 yeast strains each with a different individual specialized ribosome variant, and sorted according to expression strength: lowest levels to the left, highest on the right. The REN profiles are shown in yellow and the individual FF-based reporters are shown in green. For each reporter spectrum the luciferase activities for the wild type parent strain are indicated as a red dot, the grand mean of the given spectrum of assays as a black dot and standard deviation of the grand mean as black triangles.

The luminescence readouts from the four individual FF-based reporters each in the presence of REN, were then determined in 124 different diploid yeast strains each carrying a knockout of one of the two copies of a given single copy or duplicated RPL or RPS gene (**Figures 1** **&** **2** **; Table S1**). A standardized growth regime was used for all strains as described in Materials and Methods. After a detailed descriptive statistical analysis (**Table S2**), the luminescence readouts were sorted according to increasing expression levels of the individual FF and REN reporters. For all individual reporters, co-expressed as REN and FF pairs, we observed that the different ribosome variants generated a continuum of luciferase readouts over a several-fold range (**Figure 2**). The levels of activity for the co-expressed FF and REN reporters in the wild type parent strain always fell within the range of expression readouts for the different RPL/RPS deletion strains and within the standard deviation of the grand mean as shown (**Figure 2** **, Table S3)**.

**Figure 3:**
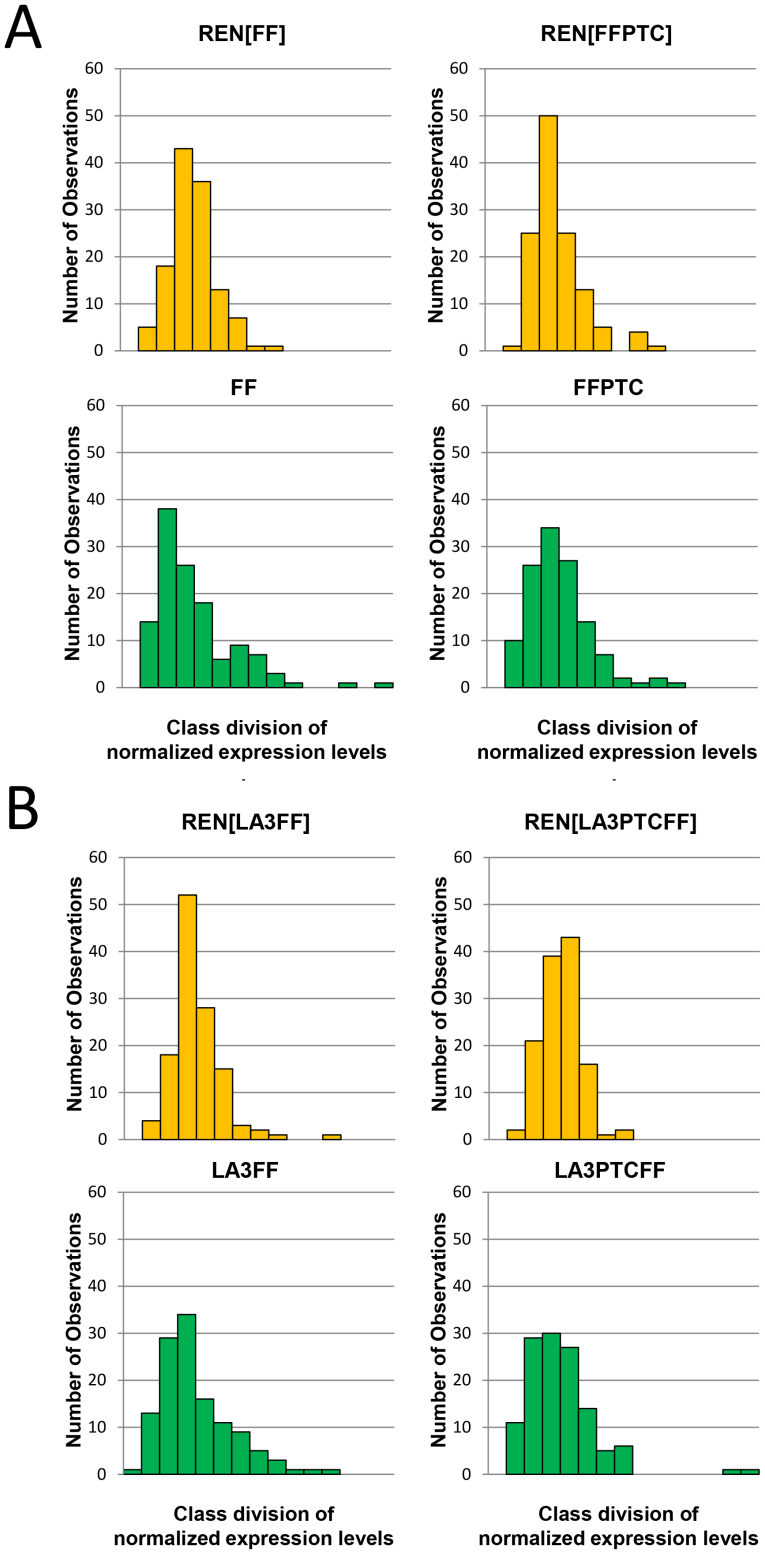
Variant ribosome screen detects specialized ribosomes. The histograms displayed show the variability in reporter luciferase readouts obtained in the set of 124 variant ribosome deletion strains, all normalized to grand mean of a given reporter expression spectrum (see **Figure 2**). The individual reporter readouts are class-divided from left to right into intervals of 0.25 fold relative to the grand mean. The REN readout histograms are in yellow and the individual FF-based reporter histograms are in green. (**A**): FF/FFPTC luciferase-based constructs; (**B**) human laminin β3-FF fusion reporter (LA3FF/LA3PTCFF) constructs.

To identify ribosomal variants that showed a significant increase or decrease for a given reporter expression signal, we next established whether the respective spectra of luminescence readouts observed for each of the different reporters were consistent for a given ribosomal variant. A histogram analysis indicated that the expression spectra observed were distinct for each of the four FF-based mRNAs, while for the REN reporter, a similar spectrum was observed irrespective of which FF-based reporter was being co-expressed in the same cell (**Figure 3**).

**Figure 4:**
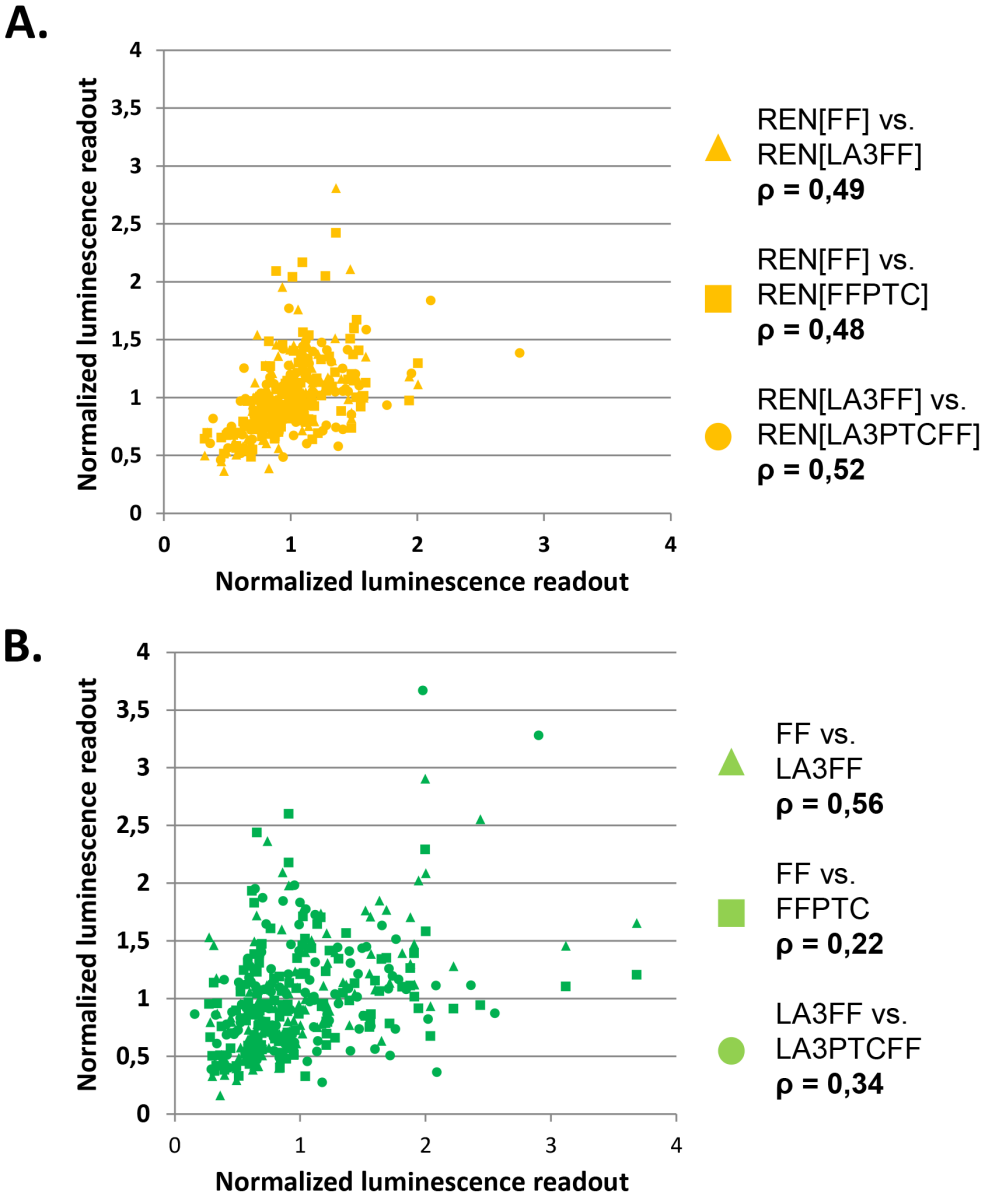
Correlation between reporter expression levels in the set of variant ribosome strains. The mRNA transcript-specific effects of RP deletion strains generate variability in reporter expression levels. Here each data point corresponds to a pairwise comparison of reporter expression levels in one RP deletion strain, normalized to the mean expression levels of each reporter. (**A**) A comparison of the differences in expression of the REN reporter paired with the different FF variants. The data points show a tight distribution around a straight line, as is expected since the REN expression constructs were identical in all cases. Outliers are most likely attributable to random errors in the reporter measurements. (**B**) A pairwise comparisons of the various FF-based luciferase reporters. Here there is a broader distribution indicating that in this case effects other than random error contribute to the deviation of the data points from a straight line e.g. specific regulation of the individual reporters in the different RP deletion strains.

We next generated correlation plots of observed differences between the different REN/FF reporter combinations to establish whether the spread of observed expression levels for each of the REN and FF reporters was due to random variation or to potential functional differences between ribosomes in the different deletion strains (**Figure 4**). All the data were normalized to the grand mean of the respective readouts across all 124 strains to facilitate comparison. Such correlation plots allowed for a pair wise comparison of reporter expression levels in individual strains. The resulting normalized REN correlation plots indicated a high correlation between all REN reporters used in the co-transformations (ρ ±0.5), meaning that the expression levels of the REN reporters in the background of different FF co-transformants were similar in a given strain and thereby largely independent of the identity of the co-expressed FF-based reporter. Interestingly, correlation of the FF reporters with their PTC derivatives showed a low correlation (ρ ±0.25) indicating that distinct specialized ribosome strains exist which preferentially increase or decrease translation of a given FF reporter mRNA, while leaving its PTC derivative unaltered and *vice versa*. Also, the correlation plot of the normalized FF set versus the normalized LA3FF set indicated an increased correlation of ρ>0.5, faithfully documenting translation of the identical FF coding sequence shared by the FF and LA3FF reporters.

To identify which ‘specialized ribosomes’ specifically increased or decreased the translation of a given luciferase reporter mRNA, we performed interference statistics (variance analysis) (**Tables S4–S11**). Variance analyses (ANOVA, α = 0.001) of validated sample size, mean expression value and standard deviations revealed specialized ribosomal variants that differed significantly from the grand mean with respect to low or high reporter expression levels (p = 0.95). This identification of selected subsets of ribosomal variants which significantly decreased or increased the translation of the respective luciferase reporter mRNAs adds further support to the proposal of the existence of a “ribosome code” [17]. This statistical analysis also revealed a significant cohort of ribosomal variants which showed little deviation from the grand mean expression level irrespective of the reporter mRNA under investigation. Therefore a significant fraction of the specialized ribosome subpopulations generated here did not impact on the ability to alter translation of any of the REN or FF mRNAs. This suggests the existence of a set of RPs not involved in any specialized mRNA translation.

**Figure 5:**
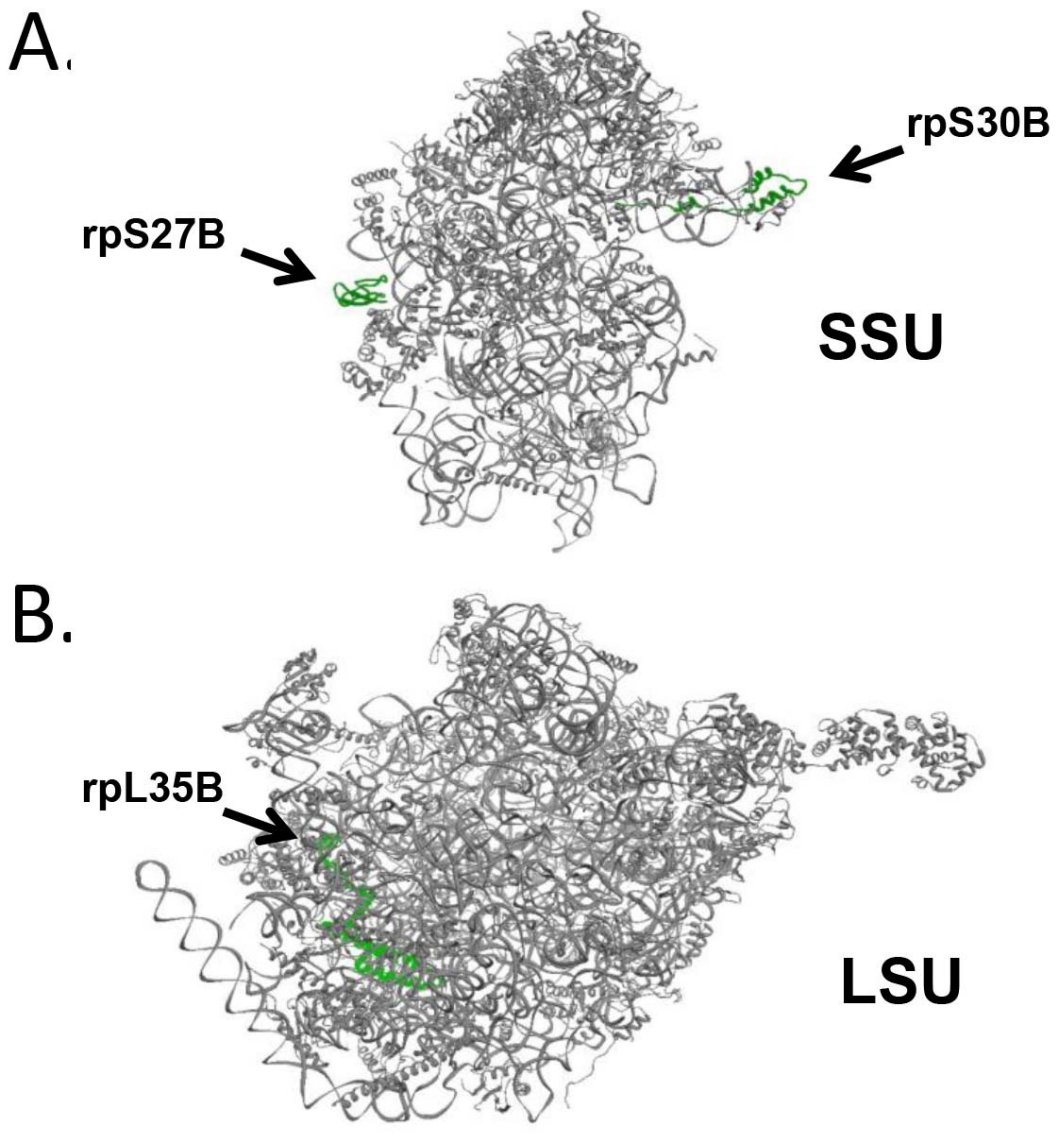
The location of specialized RPs on the yeast ribosome on the small (A) and large (B) ribosomal subunits. (**A**) On the small ribosomal subunit (SSU) RPs that increase translation of REN and FF reporter, but not PTC reporter mRNAs are highlighted in green. (**B**) The location of a RP on the large ribosomal subunit (LSU) that gave increased LAMB3-PTC reporter mRNA translation is highlighted in green. NB: Ribosome structure information [48]: USCF Chimera; PDB models: 3IZS, 3IZF, 3IZB and 3IZE.

A list of candidate specialized ribosomes with increasing specificity for the translation of a given reporter mRNA was assembled (**Table S12**) and selected candidates, rpS27B, rpS30B and rpL35B were mapped on to the 3D structure of the yeast ribosome [48] (**Figure 5** **)**. Interestingly, all of the RP candidates so identified are encoded by paralogous genes, with the respective paralogue having no significant effect on translation of any reporter mRNA (rpL35A, **Tables S5–S11)** or with non-significant alterations of reporter mRNAs (rpS27, rpS30 **Table S2).** The differential role of paralogous ribosomal proteins in translational control has been noted before [17]. Furthermore, it has been speculated, that while the position of the encoded protein on the ribosome, which is the same for both paralogues, may indeed determine translation associated functions, distinct roles for the paralogues may arise from subpopulations of paralogue-specific ribosomes, confined to distinct cellular compartments [49].

The positions of candidate RPs, rpS27B and rpS30B, are indicated on the SSU of the yeast ribosome, viewed from the inter subunit side, in **Figure 5A**. Specialized ribosomes with deficiencies in RPS27B and RPS30B, respectively, lead to a significant increase in the expression of all reporters tested except the PTC mutant variants **(Table S12**). These two RPs are located at the rim of the SSU (**Figure 5A**) in a region which shows increased flexibility during translation elongation [48]. RPs interacting with rRNA tracts involved in the elongation step of translation are known to modulate the rate of translation elongation [50]. This may be a consequence of an RP-mediated, subtle reconfiguration of rRNA tracts involved in translation elongation and hence may provide a mechanistic explanation for increase in translational efficiency in these mutants [50], [51].

Of what value is the discovery that experimentally engineering the formation of ‘specialized’ ribosomes can be used to modify the synthesis of target proteins at the post-transcriptional level? One approach would be to exploit an RNAi-based strategy to generate analogous variant ribosomes in mammalian cells and then screen for those knockdowns that show elevated levels of expression of high value protein biopharmaceuticals.

There might also be medical applications of such strategies. For example, in this study we show that the yeast strain with a sub-population of specialized ribosomes resulting from decreased levels of the RPL35B-encoding gene (**Figure 5B**), showed a near two-fold increase in the expression level of the LA3PTCFF reporter compared to the wild type (**Table S12**) despite the fact that LA3PTCFF mRNA levels in comparison to WT were only slightly reduced in the RPL35B deletion mutant (**Figure S1**). Importantly, the translation of all other reporter mRNAs, including the FFPTC reporter, was largely unaltered in this strain. Interestingly, the rpL35B protein, like the candidates on the small subunit, is accessible to different molecular environments, as it is located on the solvent side of the LSU, at the site where the nascent protein chain leaves the ribosome through the protein exit tunnel. However, most of the RPs on the LSU are exposed to the solvent side, but only the rpL35 proteins contact rpL25 and rpL39 [52]. These proteins in turn interact with and modify the nascent protein exit tunnel during ribosome stalling [52], a process also observed when a PTC codon is encountered in the A-site of the ribosome. The protein exit tunnel is flexible and may be functionally altered to support increased readthrough of premature termination codons by long-range interactions between the ribosomal subunits [53]. Interestingly, rpL36A, the reduced copy number of which selectively increases FFPTC expression but not LA3PTCFF expression also contacts the protein exit tunnel, albeit at a site different from rpL35B [27]. These observations suggest a ribosomal code for customized PTC readthrough. Indeed, there are additional RPs that modulate dynamics of the protein exit tunnel, which are not in contact with rpL35B or rpL36 [52]. This suggests, that these RPs might serve as regulators for other PTC-containing mRNAs. Thus, our findings confirm that the rational modification of a eukaryotic ribosome can customize increased translation of a specific, disease-associated mRNA and may represent a novel therapeutic strategy for the future.

The use of a specialized ribosome screen as described here can also be used to complement other studies on the functional specialization of ribosomes in bacteria and eukaryotes. For example, manipulation of ribosome structure and function by engineering rRNA tracts or the translation machinery to direct mRNA-specific protein synthesis may reveal unprecedented insights into molecular mechanisms controlling translation, but may have functional consequences for bulk translation, possibly leading to host cell death [12], [34], [35]. However, each specialized ribosome used in our screen would be distinguished by a subtle structural and possibly functional alteration in the ribosome that leads to a change within the dynamic range of the translation machinery (**Figure 2**). This could, for example, result in increasing translation elongation efficiency of canonical sequences, as in the case of rpS27B and rpS30B specialized ribosomes, or favor readthrough of a distinct PTC mutation, as in the case of rpL35B specialized ribosomes. We anticipate that the RPs, including modified forms thereof e.g. phosphorylated species, represent a vast functional space for changing the dynamic of target mRNA translation.

## Supporting Information

Figure S1 **Stability and integrity of LA3FF and LA3PTCFF mRNAs.** (A) mRNA constructs monitored by RT-PCR are depicted. The positions of the primer pairs are indicated (1) to (4). (B) RT-PCR results obtained for the parental BY4743 strain expressing either the LA3FF or the LA3PTCFF mRNAs as described in Materials and Methods. The y-axis shows the original fold expression Δct-values. Results for primer pairs (1), (2) and (3) for the LA3FF construct and for the LA3PTCFF constructs are depicted as grey bars, respectively. (C) The same analysis as shown in (B) but this time carried out in the +/rpL35B deletion strain with the corresponding RT-PCR results for the LA3FF and LA3PTCFF mRNA constructs shown. For comparison, primer pair (4) was used to monitor expression levels of the REN coding sequence. The RT-PCR experiments were performed in triplicate assays and the values represent the mean ± s.d. for three experiments.
(PDF)

Table S1 **Listing of large (LSU) and small (SSU) subunit variant RP deletion strains.**
(XLSX)

Table S2 **Descriptive statistical analysis of luciferase reporter readout data.**
(XLSX)

Table S3 **Grand mean of reporter expression profiling.**
(XLSX)

Table S4 **One way analysis of variance of FF reporter readouts.**
(DOCX)

Table S5 **One way analysis of variance of FFPTC reporter readouts.**
(DOCX)

Table S6 **One way analysis of variance of LA3FF reporter readouts.**
(DOCX)

Table S7 **One way analysis of variance of LA3PTCFF reporter readouts.**
(DOCX)

Table S8 **One way analysis of variance of REN[FF] reporter readouts.**
(DOCX)

Table S9 **One way analysis of variance of REN[FFPTC] reporter readouts.**
(DOCX)

Table S10 **One way analysis of variance of REN[LA3FF] reporter readouts.**
(DOCX)

Table S11 **One way analysis of variance of REN[LA3PTCFF] reporter readouts.**
(DOCX)

Table S12 **Heat map of specialized ribosome strains with increasing specificity to increase LAMB3-PTC expression, while leaving expression of the other reporters largely unaltered.**
(XLSX)
