# Supplementary figures and images for: Specialized Yeast Ribosomes: A Customized Tool for Selective mRNA Translation
Source: PLoS One. 2013 Jul 8;8(7):e67609. doi: 10.1371/journal.pone.0067609 (PMC3704640; doi:10.1371/journal.pone.0067609)

**A.**

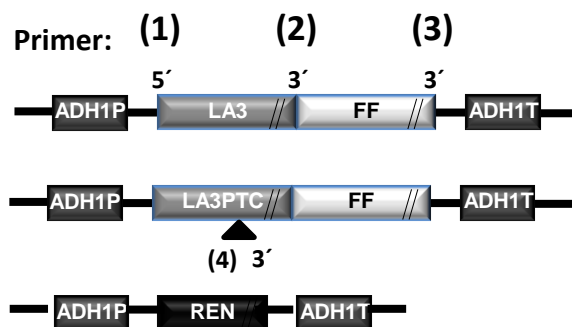

**B.**

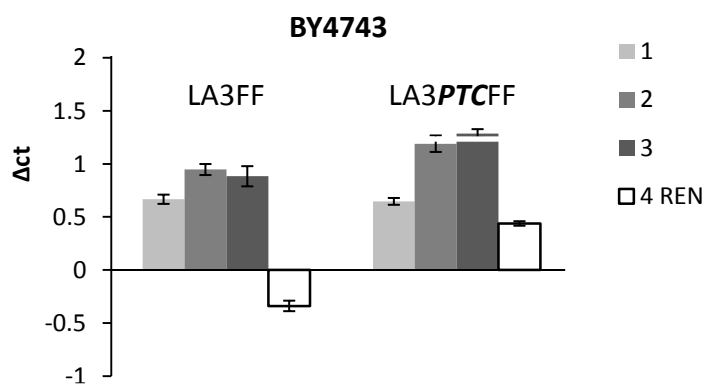

**C.**

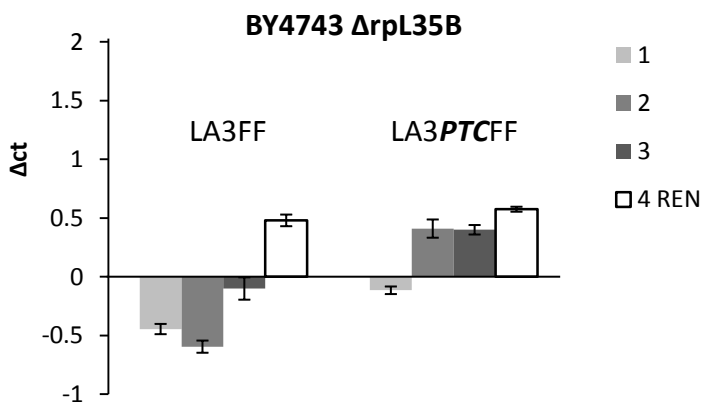

Supplement: Figure S1 — Stability and integrity of LA3FF and LA3PTCFF mRNAs. (A) mRNA constructs monitored by RT-PCR are depicted. The positions of the primer pairs are indicated (1) to (4). (B) RT-PCR results obtained for the parental BY4743 strain expressing either the LA3FF or the LA3PTCFF mRNAs as described in Materials and Methods. The y-axis shows the original fold expression Δct-values. Results for primer pairs (1), (2) and (3) for the LA3FF construct and for the LA3PTCFF constructs are depicted as grey bars, respectively. (C) The same analysis as shown in (B) but this time carried out in the +/rpL35B deletion strain with the corresponding RT-PCR results for the LA3FF and LA3PTCFF mRNA constructs shown. For comparison, primer pair (4) was used to monitor expression levels of the REN coding sequence. The RT-PCR experiments were performed in triplicate assays and the values represent the mean ± s.d. for three experiments. (PDF) [file pone.0067609.s001.pdf]
