# Supplementary material for: Specialized Yeast Ribosomes: A Customized Tool for Selective mRNA Translation
Source: PLoS One. 2013 Jul 8;8(7):e67609. doi: 10.1371/journal.pone.0067609 (PMC3704640; doi:10.1371/journal.pone.0067609)
Supplement: Table S4 — One way analysis of variance of FF reporter readouts. (DOCX) [file pone.0067609.s005.docx]

**Supplementary Table S4:** One way analysis of variance of FF reporter readouts.

**Data source:** FF in ANOVAs.SNB

**Group Name N Missing Mean Std Dev SEM**

dRpS0A 6 0 13434316,670 689563,211 281513,002

dRpS0B 6 0 10541733,330 447819,489 182821,541

dRpS1A 6 0 14342350,000 783988,206 320061,845

dRpS1B 6 0 10628816,670 450727,986 184008,930

dRpS2 11 0 11839136,364 576706,999 173883,703

dRpS3 6 0 19549800,000 1296235,230 529185,817

dRpS4A 4 0 13677650,000 750495,661 375247,830

dRpS4B 6 0 22122016,667 1531320,106 625158,815

dRpS5 6 0 23471300,000 1341131,843 547514,782

dRpS6A 12 0 6714554,167 1120272,945 323394,943

dRpS6B 12 0 6085540,000 441452,026 127436,223

dRpS7A 6 0 7281845,000 896420,561 365962,162

dRpS7B 6 0 17844950,000 1031057,932 420927,638

dRpS8A 6 0 11007816,667 1018231,163 415691,132

dRpS9A 12 0 25108025,000 2667451,945 770027,049

dRpS9B 12 0 18029700,000 1584225,239 457326,434

dRpS10A 6 0 11330533,333 1120779,983 457556,512

dRpS10B 6 0 20442033,333 1860297,240 759463,168

dRpS11A 6 0 12567050,000 1364189,297 556927,948

dRpS11B 6 0 15935233,333 2327350,804 950136,987

dRpS12 6 0 14961150,000 595000,695 242908,017

dRpS13 5 0 6428864,000 751615,199 336132,536

dRpS14A 6 0 26083000,000 1426108,077 582206,184

dRpS14B 6 0 41860950,000 4112257,291 1678822,009

dRpS15 6 0 33425200,000 1481676,542 604891,915

dRpS16A 6 0 21951483,333 803614,319 328074,172

dRpS16B 6 0 24496533,333 1672600,580 682836,327

dRpS17A 6 0 27548766,667 2340612,915 955551,221

dRpS17B 6 0 35160150,000 1964033,616 801813,366

dRpS18A 6 0 28149150,000 1744471,670 712177,577

dRpS18B 6 0 30014450,000 2298949,727 938542,296

dRpS19A 6 0 41183366,667 2176831,008 888687,538

dRpS19B 6 0 17228516,667 817833,982 333879,325

dRpS20 6 0 7791905,000 1031659,565 421173,254

dRpS21A 6 0 14607833,333 793756,901 324049,898

dRpS21B 6 0 8533728,333 773526,969 315791,063

dRpS22A 6 0 14138150,000 956367,136 390435,248

dRpS22B 6 0 79318200,000 3155055,631 1288046,068

dRpS23A 6 0 26566800,000 3625126,001 1479951,493

dRpS23B 3 0 8053763,333 164413,267 94924,044

dRpS24A 6 0 17065116,667 898465,215 366796,888

dRpS24B 6 0 12113983,333 2005877,450 818896,040

dRpS25A 6 0 14054416,667 1500142,687 612430,687

dRpS25B 6 0 20473366,667 1525079,490 622611,095

dRpS26B 5 0 33704340,000 877147,344 392272,217

dRpS27A 6 0 40446916,667 2504165,055 1022321,103

dRpS27B 6 0 43129616,667 1245747,762 508574,394

dRpS28A 6 0 29454983,333 2813807,646 1148732,161

dRpS28B 6 0 32734666,667 4307460,263 1758513,289

dRpS29A 10 0 22026790,000 3467732,255 1096593,224

dRpS29B 12 0 13728066,667 1735298,638 500937,568

dRpS30A 6 0 25528933,333 1178785,291 481237,080

dRpS30B 4 0 39077400,000 2775661,265 1387830,633

dRpS31 6 0 19417266,667 1595803,586 651484,086

dRpP0 6 0 27646133,333 3186205,480 1300762,940

dRpP1A 6 0 16803466,667 2299937,787 938945,670

dRpP1B 6 0 27210283,333 3327036,629 1358257,016

dRpP2A 6 0 25745516,667 673833,826 275091,508

dRpP2B 6 0 25168083,333 4141478,766 1690751,626

dRpL1A 6 0 13761450,000 965889,426 394322,707

dRpL1B 6 0 15765766,667 573815,467 234259,183

dRpL2A 6 0 14655333,333 961760,333 392637,012

dRpL2B 6 0 30773150,000 1709316,784 697825,655

dRpL3 5 0 10353302,000 449637,873 201084,170

dRpL4A 12 0 23150616,667 1751402,496 505586,351

dRpL6A 6 0 10681033,333 258831,441 105667,493

dRpL6B 6 0 9916333,333 534731,743 218303,320

dRpL7A 6 0 13532966,667 815596,507 332965,880

dRpL7B 6 0 14752750,000 468433,221 191237,062

dRpL8A 6 0 13982933,333 1293842,460 528208,972

dRpL8B 6 0 19480766,667 799054,878 326212,788

dRpL9A 6 0 14497450,000 442767,744 180759,175

dRpL10 6 0 11852200,000 730495,133 298223,389

dRpL11B 6 0 10575916,667 605124,792 247041,162

dRpL12A 3 0 13865133,333 1543119,536 890920,480

dRpL12B 6 0 19415083,333 965603,876 394206,132

dRpL13A 6 0 4401568,333 1528353,807 623947,829

dRpL13B 6 0 9620695,000 349712,019 142769,334

dRpL14A 6 0 11144366,667 652436,966 266356,276

dRpL15A 4 0 26579700,000 101490,000 50745,000

dRpL15B 6 0 11761183,333 1397614,779 570573,844

dRpL16A 6 0 13829066,667 726141,349 296445,964

dRpL16B 6 0 10887450,000 382451,397 156135,129

dRpL17A 6 0 13460416,667 791580,256 323161,286

dRpL18A 6 0 15473683,333 984569,052 401948,632

dRpL18B 3 0 8468883,333 113316,566 65423,350

dRpL19A 6 0 18916800,000 1692482,216 690952,971

dRpL19B 12 0 10212738,333 610923,308 176358,368

dRpL20A 6 0 12118733,333 538589,379 219878,193

dRpL20B 6 0 35844566,667 2749754,128 1122582,422

dRpL21A 6 0 19488566,667 1969471,725 804033,465

dRpL21B 6 0 10861066,667 959736,238 391810,678

dRpL22A 5 0 5140922,000 474856,896 212362,460

dRpL22B 6 0 15986450,000 1020137,622 416469,440

dRpL23A 6 0 11711733,333 1213609,126 495453,851

dRpL23B 6 0 7308040,000 329955,758 134703,874

dRpL24A 6 0 10560681,667 708213,092 289126,784

dRpL24B 6 0 9462015,000 747282,192 305076,677

dRpL25 6 0 16744633,333 844798,517 344887,550

dRpL26A 6 0 15056933,333 1124487,102 459069,937

dRpL26B 12 0 10609390,833 1011656,573 292040,097

dRpL27A 6 0 32231183,333 3656800,503 1492882,554

dRpL27B 6 0 50285566,667 2049537,906 836720,346

dRpL28 6 0 10186563,333 481720,406 196661,532

dRpL29 4 0 16160700,000 525587,665 262793,833

dRpL30 6 0 5350736,667 485785,090 198320,933

dRpL31A 6 0 39296000,000 1325273,382 541040,593

dRpL32 6 0 14350533,333 569535,687 232511,971

dRpL33A 6 0 7203576,667 887344,638 362256,932

dRpL33B 6 0 32896466,667 1483230,998 605526,519

dRpL34A 6 0 11231133,333 439768,297 179534,655

dRpL34B 6 0 10245525,000 426097,995 173953,778

dRpL35A 6 0 21639583,333 1538544,722 628108,253

dRpL35B 6 0 10703483,333 443757,192 181163,115

dRpL36A 5 0 12344120,000 573407,108 256435,454

dRpL37A 6 0 30312666,667 1448756,923 591452,537

dRpL37B 6 0 12807050,000 1089603,050 444828,582

dRpL38 3 0 16804100,000 626313,029 361601,996

dRpL40A 6 0 10219485,000 460202,318 187876,810

dRpL40B 6 0 9466336,667 910485,591 371704,186

dRpL41A 4 0 11087625,000 1046882,309 523441,155

dRpL41B 6 0 15644150,000 425801,478 173832,725

dRpL42A 6 0 19642816,667 2111581,737 862049,634

dRpL43B 6 0 9198245,000 2639022,888 1077376,583

Grand Mean 124 0 18475450,197 11001648,387 987977,191

**Source of Variation DF SS MS F P**

Between Groups 124 9,013E+016 7,269E+014 33,863 <0,001

Residual 773 1,659E+016 2,146E+013

Total 897 1,067E+017

The differences in the mean values among the treatment groups are greater than would be expected by chance; there is a statistically significant difference (P = <0,001).

Power of performed test with alpha = 0,050: 1,000

Multiple Comparisons versus Control Group (Holm-Sidak method):

Overall significance level = 0,05

Comparisons for factor:

**Comparison Diff of Means t Unadjusted P Critical Level Significant?**

Grand Mean vs. dRpS22B 60842749,803 31,417 2,966E-140 0,000 Yes

Grand Mean vs. dRpL27B 31810116,470 16,425 3,140E-052 0,000 Yes

Grand Mean vs. dRpS27B 24654166,470 12,730 7,706E-034 0,000 Yes

Grand Mean vs. dRpS14B 23385499,803 12,075 7,053E-031 0,000 Yes

Grand Mean vs. dRpS19A 22707916,470 11,725 2,439E-029 0,000 Yes

Grand Mean vs. dRpS27A 21971466,470 11,345 1,058E-027 0,000 Yes

Grand Mean vs. dRpL31A 20820549,803 10,751 3,212E-025 0,000 Yes

Grand Mean vs. dRpL20B 17369116,470 8,969 2,232E-018 0,000 Yes

Grand Mean vs. dRpS6B 12389910,197 8,846 6,098E-018 0,000 Yes

Grand Mean vs. dRpS30B 20601949,803 8,754 1,288E-017 0,000 Yes

Grand Mean vs. dRpS17B 16684699,803 8,615 3,898E-017 0,000 Yes

Grand Mean vs. dRpS6A 11760896,030 8,397 2,183E-016 0,000 Yes

Grand Mean vs. dRpS15 14949749,803 7,719 3,615E-014 0,000 Yes

Grand Mean vs. dRpL33B 14421016,470 7,446 2,564E-013 0,000 Yes

Grand Mean vs. dRpS28B 14259216,470 7,363 4,616E-013 0,000 Yes

Grand Mean vs. dRpL13A 14073881,864 7,267 8,989E-013 0,000 Yes

Grand Mean vs. dRpS26B 15228889,803 7,206 1,369E-012 0,000 Yes

Grand Mean vs. dRpL27A 13755733,136 7,103 2,774E-012 0,000 Yes

Grand Mean vs. dRpL30 13124713,530 6,777 2,432E-011 0,000 Yes

Grand Mean vs. dRpL2B 12297699,803 6,350 0,000000000367 0,000 Yes

Grand Mean vs. dRpL22A 13334528,197 6,310 0,000000000470 0,000 Yes

Grand Mean vs. dRpL37A 11837216,470 6,112 0,00000000156 0,000 Yes

Grand Mean vs. dRpS18B 11538999,803 5,958 0,00000000387 0,001 Yes

Grand Mean vs. dRpL19B 8262711,864 5,899 0,00000000546 0,001 Yes

Grand Mean vs. dRpL33A 11271873,530 5,820 0,00000000860 0,001 Yes

Grand Mean vs. dRpS7A 11193605,197 5,780 0,0000000108 0,001 Yes

Grand Mean vs. dRpL23B 11167410,197 5,766 0,0000000117 0,001 Yes

Grand Mean vs. dRpS13 12046586,197 5,700 0,0000000170 0,001 Yes

Grand Mean vs. dRpS28A 10979533,136 5,669 0,0000000202 0,001 Yes

Grand Mean vs. dRpL26B 7866059,364 5,616 0,0000000273 0,001 Yes

Grand Mean vs. dRpS20 10683545,197 5,517 0,0000000472 0,001 Yes

Grand Mean vs. dRpS21B 9941721,864 5,134 0,000000360 0,001 Yes

Grand Mean vs. dRpS18A 9673699,803 4,995 0,000000727 0,001 Yes

Grand Mean vs. dRpL43B 9277205,197 4,790 0,00000200 0,001 Yes

Grand Mean vs. dRpP0 9170683,136 4,735 0,00000260 0,001 Yes

Grand Mean vs. dRpS9A 6632574,803 4,735 0,00000260 0,001 Yes

Grand Mean vs. dRpS17A 9073316,470 4,685 0,00000331 0,001 Yes

Grand Mean vs. dRpL24B 9013435,197 4,654 0,00000383 0,001 Yes

Grand Mean vs. dRpL40B 9009113,530 4,652 0,00000387 0,001 Yes

Grand Mean vs. dRpL13B 8854755,197 4,572 0,00000562 0,001 Yes

Grand Mean vs. dRpS2 6636313,833 4,553 0,00000614 0,001 Yes

Grand Mean vs. dRpP1B 8734833,136 4,510 0,00000748 0,001 Yes

Grand Mean vs. dRpL6B 8559116,864 4,420 0,0000113 0,001 Yes

Grand Mean vs. dRpL28 8288886,864 4,280 0,0000210 0,001 Yes

Grand Mean vs. dRpL40A 8255965,197 4,263 0,0000227 0,001 Yes

Grand Mean vs. dRpL34B 8229925,197 4,250 0,0000240 0,001 Yes

Grand Mean vs. dRpS23A 8091349,803 4,178 0,0000328 0,001 Yes

Grand Mean vs. dRpS0B 7933716,867 4,097 0,0000463 0,001 Yes

Grand Mean vs. dRpL24A 7914768,530 4,087 0,0000483 0,001 Yes

Grand Mean vs. dRpL11B 7899533,530 4,079 0,0000499 0,001 Yes

Grand Mean vs. dRpS1B 7846633,527 4,052 0,0000560 0,001 Yes

Grand Mean vs. dRpL6A 7794416,864 4,025 0,0000627 0,001 Yes

Grand Mean vs. dRpL35B 7771966,864 4,013 0,0000657 0,001 Yes

Grand Mean vs. dRpL21B 7614383,530 3,932 0,0000919 0,001 Yes

Grand Mean vs. dRpS14A 7607549,803 3,928 0,0000932 0,001 Yes

Grand Mean vs. dRpL16B 7588000,197 3,918 0,0000971 0,001 Yes

Grand Mean vs. dRpS8A 7467633,530 3,856 0,000125 0,001 Yes

Grand Mean vs. dRpS23B 10421686,864 3,850 0,000128 0,001 Yes

Grand Mean vs. dRpL3 8122148,197 3,843 0,000131 0,001 Yes

Grand Mean vs. dRpL14A 7331083,530 3,785 0,000165 0,001 Yes

Grand Mean vs. dRpP2A 7270066,470 3,754 0,000187 0,001 Yes

Grand Mean vs. dRpL34A 7244316,864 3,741 0,000197 0,001 Yes

Grand Mean vs. dRpL18B 10006566,864 3,697 0,000234 0,001 Yes

Grand Mean vs. dRpS10A 7144916,864 3,689 0,000241 0,001 Yes

Grand Mean vs. dRpS30A 7053483,136 3,642 0,000288 0,001 Yes

Grand Mean vs. dRpL23A 6763716,864 3,493 0,000506 0,001 Yes

Grand Mean vs. dRpL15B 6714266,864 3,467 0,000555 0,001 Yes

Grand Mean vs. dRpP2B 6692633,136 3,456 0,000579 0,001 Yes

Grand Mean vs. dRpL15A 8104249,803 3,443 0,000605 0,001 Yes

Grand Mean vs. dRpL10 6623250,197 3,420 0,000659 0,001 Yes

Grand Mean vs. dRpS29B 4747383,530 3,389 0,000736 0,001 Yes

Grand Mean vs. dRpL4A 4675166,470 3,338 0,000885 0,001 Yes

Grand Mean vs. dRpS24B 6361466,864 3,285 0,00107 0,001 No

Grand Mean vs. dRpL20A 6356716,864 3,282 0,00108 0,001 No

Grand Mean vs. dRpL41A 7387825,197 3,139 0,00176 0,001 No

Grand Mean vs. dRpS16B 6021083,136 3,109 0,00195 0,001 No

Grand Mean vs. dRpS11A 5908400,197 3,051 0,00236 0,001 No

Grand Mean vs. dRpL37B 5668400,197 2,927 0,00352 0,001 No

Grand Mean vs. dRpL36A 6131330,197 2,901 0,00382 0,001 No

Grand Mean vs. dRpS0A 5041133,527 2,603 0,00942 0,001 No

Grand Mean vs. dRpL17A 5015033,530 2,590 0,00979 0,001 No

Grand Mean vs. dRpS5 4995849,803 2,580 0,0101 0,001 No

Grand Mean vs. dRpL7A 4942483,530 2,552 0,0109 0,001 No

Grand Mean vs. dRpL1A 4714000,197 2,434 0,0152 0,001 No

Grand Mean vs. dRpL16A 4646383,530 2,399 0,0167 0,001 No

Grand Mean vs. dRpS29A 3551339,803 2,332 0,0200 0,001 No

Grand Mean vs. dRpL8A 4492516,864 2,320 0,0206 0,001 No

Grand Mean vs. dRpS25A 4421033,530 2,283 0,0227 0,001 No

Grand Mean vs. dRpS22A 4337300,197 2,240 0,0254 0,001 No

Grand Mean vs. dRpS1A 4133100,197 2,134 0,0331 0,001 No

Grand Mean vs. dRpL32 4124916,864 2,130 0,0335 0,002 No

Grand Mean vs. dRpL9A 3978000,197 2,054 0,0403 0,002 No

Grand Mean vs. dRpS4A 4797800,197 2,039 0,0418 0,002 No

Grand Mean vs. dRpS21A 3867616,864 1,997 0,0462 0,002 No

Grand Mean vs. dRpL2A 3820116,864 1,973 0,0489 0,002 No

Grand Mean vs. dRpL7B 3722700,197 1,922 0,0549 0,002 No

Grand Mean vs. dRpS4B 3646566,470 1,883 0,0601 0,002 No

Grand Mean vs. dRpS12 3514300,197 1,815 0,0700 0,002 No

Grand Mean vs. dRpS16A 3476033,136 1,795 0,0731 0,002 No

Grand Mean vs. dRpL26A 3418516,864 1,765 0,0779 0,002 No

Grand Mean vs. dRpL12A 4610316,864 1,703 0,0890 0,002 No

Grand Mean vs. dRpL35A 3164133,136 1,634 0,103 0,002 No

Grand Mean vs. dRpL18A 3001766,864 1,550 0,122 0,002 No

Grand Mean vs. dRpL41B 2831300,197 1,462 0,144 0,002 No

Grand Mean vs. dRpL1B 2709683,530 1,399 0,162 0,003 No

Grand Mean vs. dRpS11B 2540216,864 1,312 0,190 0,003 No

Grand Mean vs. dRpL22B 2489000,197 1,285 0,199 0,003 No

Grand Mean vs. dRpS25B 1997916,470 1,032 0,303 0,003 No

Grand Mean vs. dRpS10B 1966583,136 1,015 0,310 0,003 No

Grand Mean vs. dRpL29 2314750,197 0,984 0,326 0,003 No

Grand Mean vs. dRpL25 1730816,864 0,894 0,372 0,004 No

Grand Mean vs. dRpP1A 1671983,530 0,863 0,388 0,004 No

Grand Mean vs. dRpS24A 1410333,530 0,728 0,467 0,004 No

Grand Mean vs. dRpS19B 1246933,530 0,644 0,520 0,005 No

Grand Mean vs. dRpL38 1671350,197 0,617 0,537 0,005 No

Grand Mean vs. dRpL42A 1167366,470 0,603 0,547 0,006 No

Grand Mean vs. dRpS3 1074349,803 0,555 0,579 0,006 No

Grand Mean vs. dRpL21A 1013116,470 0,523 0,601 0,007 No

Grand Mean vs. dRpL8B 1005316,470 0,519 0,604 0,009 No

Grand Mean vs. dRpS31 941816,470 0,486 0,627 0,010 No

Grand Mean vs. dRpL12B 939633,136 0,485 0,628 0,013 No

Grand Mean vs. dRpS7B 630500,197 0,326 0,745 0,017 No

Grand Mean vs. dRpS9B 445750,197 0,318 0,750 0,025 No

Grand Mean vs. dRpL19A 441349,803 0,228 0,820 0,050 No
