# Supplementary material for: Specialized Yeast Ribosomes: A Customized Tool for Selective mRNA Translation
Source: PLoS One. 2013 Jul 8;8(7):e67609. doi: 10.1371/journal.pone.0067609 (PMC3704640; doi:10.1371/journal.pone.0067609)
Supplement: Table S5 — One way analysis of variance of FFPTC reporter readouts. (DOCX) [file pone.0067609.s006.docx]

**Supplementary Table S5:** One way analysis of variance of FFPTC reporter readouts.

**One Way Analysis of Variance**

**Data source:** FFPTC in ANOVAs.SNB

**Group Name N Missing Mean Std Dev SEM**

RpS0A 6 0 2229,572 614,192 250,743

RpS0B 5 0 4393,390 350,296 156,657

RpS1A 6 0 3735,138 475,334 194,054

RpS1B 6 0 2018,383 437,641 178,666

RpS2 12 0 6092,602 1691,494 488,292

RpS3 6 0 10657,812 950,084 387,870

RpS4A 6 0 6773,677 2983,914 1218,178

RpS4B 6 0 5973,882 1952,655 797,168

RpS5 6 0 7029,558 3886,776 1586,770

RpS6A 6 0 5564,067 1694,050 691,593

RpS6B 6 0 3249,728 1327,453 541,930

RpS7A 6 0 4690,375 2284,723 932,734

RpS7B 6 0 3076,338 1302,672 531,814

RpS8A 6 0 1597,268 337,662 137,850

RpS9A 12 0 8333,950 2161,843 624,070

RpS9B 12 0 4119,629 998,807 288,331

RpS10A 6 0 5465,528 409,001 166,974

RpS10B 6 0 2490,338 933,392 381,056

RpS11A 6 0 6387,783 259,465 105,926

RpS11B 6 0 3469,885 327,357 133,643

RpS12 5 0 7209,462 536,905 240,111

RpS13 6 0 2452,755 869,022 354,777

RpS14A 6 0 3398,142 1111,573 453,798

RpS14B 6 0 4475,258 1430,864 584,148

RpS15 6 0 3683,935 1200,272 490,009

RpS16A 6 0 7146,150 2818,297 1150,565

RpS16B 6 0 8054,867 3179,575 1298,056

RpS17A 6 0 3219,905 786,305 321,008

RpS17B 6 0 5201,197 1644,343 671,300

RpS18A 6 0 6582,288 2956,190 1206,860

RpS18B 6 0 4810,438 1584,486 646,864

RpS19A 6 0 4972,688 507,633 207,240

RpS19B 6 0 3185,413 603,852 246,522

RpS20 6 0 2481,695 577,753 235,867

RpS21A 6 0 4379,047 900,932 367,804

RpS21B 6 0 1867,318 389,063 158,834

RpS22A 6 0 3241,458 943,779 385,296

RpS22B 6 0 5897,892 1665,734 680,033

RpS23A 6 0 6930,993 1480,422 604,380

RpS23B 6 0 3709,100 826,998 337,621

RpS24A 6 0 5743,532 1066,719 435,486

RpS24B 6 0 2547,512 608,816 248,548

RpS25A 6 0 4398,285 1775,665 724,912

RpS25B 6 0 2509,573 694,341 283,464

RpS26B 6 0 4229,578 1531,249 625,130

RpS27A 4 0 6178,630 1461,968 730,984

RpS27B 6 0 7738,952 1928,420 787,274

RpS28A 6 0 7666,857 458,440 187,157

RpS28B 6 0 4154,095 1871,690 764,114

RpS29A 12 0 8369,837 2384,304 688,289

RpS29B 12 0 6092,474 1014,517 292,866

RpS30A 6 0 4669,275 1037,943 423,738

RpS30B 6 0 5396,110 1468,523 599,522

RpS31 6 0 4638,855 974,377 397,788

RpP0 6 0 4449,400 388,987 158,803

RpP1A 4 0 1846,650 32,772 16,386

RpP1B 6 0 7693,490 2260,748 922,947

RpP2A 6 0 6558,113 723,038 295,179

RpP2B 5 0 4575,812 761,891 340,728

RpL1A 6 0 6082,288 2514,444 1026,517

RpL1B 6 0 7676,433 2236,033 912,857

RpL2A 6 0 14788,267 2816,424 1149,800

RpL2B 6 0 7911,697 2536,948 1035,705

RpL3 6 0 5181,543 1573,253 642,278

RpL4A 12 0 6438,904 1729,573 499,285

RpL6A 6 0 3559,730 1237,057 505,026

RpL6B 6 0 10988,837 2784,950 1136,951

RpL7A 6 0 2267,197 886,201 361,790

RpL7B 6 0 6449,548 1732,897 707,452

RpL8A 6 0 6048,125 1179,691 481,607

RpL8B 6 0 4546,408 1284,705 524,479

RpL9A 6 0 4003,100 1252,598 511,371

RpL10 6 0 4301,742 1759,755 718,417

RpL11B 6 0 13871,662 3884,346 1585,778

RpL12A 4 0 3007,480 1934,199 967,100

RpL12B 6 0 7025,880 1520,661 620,807

RpL13A 4 0 5411,298 1847,601 923,801

RpL13B 6 0 2447,807 685,803 279,978

RpL14A 5 0 3386,628 287,516 128,581

RpL15A 6 0 7623,202 3452,693 1409,556

RpL15B 5 0 5408,844 2159,626 965,814

RpL16A 6 0 5372,452 1754,929 716,447

RpL16B 6 0 7437,297 1337,911 546,200

RpL17A 3 0 6768,073 280,309 161,836

RpL18A 6 0 4121,478 1609,789 657,194

RpL18B 6 0 6020,788 1688,223 689,214

RpL19A 6 0 5297,053 1305,479 532,960

RpL19B 12 0 3896,372 537,526 155,170

RpL20A 6 0 4319,248 1514,754 618,396

RpL20B 6 0 5185,018 1223,525 499,502

RpL21A 6 0 3387,070 920,705 375,876

RpL21B 6 0 4418,142 1217,070 496,867

RpL22A 6 0 2113,238 447,409 182,654

RpL22B 6 0 4927,362 968,974 395,582

RpL23A 6 0 4541,762 1485,891 606,612

RpL23B 5 0 3969,752 458,294 204,955

RpL24A 5 0 6999,414 1196,554 535,115

RpL24B 6 0 3350,880 1172,301 478,590

RpL25 6 0 8636,695 3077,613 1256,430

RpL26A 6 0 4000,677 820,565 334,994

RpL26B 12 0 5932,273 2039,216 588,671

RpL27A 6 0 13037,143 2222,142 907,186

RpL27B 5 0 6281,860 2035,667 910,378

RpL28 6 0 4887,050 560,928 228,998

RpL29 4 0 6887,093 47,278 23,639

RpL30 6 0 2371,653 1083,241 442,231

RpL31A 6 0 5350,387 1025,068 418,482

RpL32 6 0 2705,513 747,083 304,995

RpL33A 6 0 3229,243 509,285 207,915

RpL33B 3 0 3836,960 480,472 277,401

RpL34A 6 0 3175,123 1000,642 408,510

RpL34B 6 0 2909,788 386,602 157,830

RpL35A 6 0 5961,735 1579,571 644,857

RpL35B 6 0 6088,173 2764,409 1128,565

RpL36A 6 0 9132,748 2727,097 1113,333

RpL37A 6 0 6323,630 1294,848 528,619

RpL37B 6 0 5587,098 1709,834 698,037

RpL38 6 0 6916,103 1498,885 611,917

RpL40A 4 0 10403,985 762,879 381,440

RpL40B 6 0 5505,902 764,925 312,279

RpL41A 6 0 8334,420 1486,111 606,702

RpL41B 6 0 6187,880 1036,051 422,966

RpL42A 6 0 4529,932 1668,100 680,999

RpL43B 6 0 4592,417 1333,132 544,249

Grand Mean 124 0 5347,286 2371,291 212,948

**Source of Variation DF SS MS F P**

Between Groups 124 4174219481,259 33663060,333 10,926 <0,001

Residual 765 2356936351,984 3080962,552

Total 889 6531155833,244

The differences in the mean values among the treatment groups are greater than would be expected by chance; there is a statistically significant difference (P = <0,001).

Power of performed test with alpha = 0,050: 1,000

Multiple Comparisons versus Control Group (Holm-Sidak method):

Overall significance level = 0,05

Comparisons for factor:

**Comparison Diff of Means t Unadjusted P Critical Level Significant?**

Grand Mean vs. RpL2A 9440,981 12,867 1,934E-034 0,000 Yes

Grand Mean vs. RpL11B 8524,376 11,618 7,504E-029 0,000 Yes

Grand Mean vs. RpL27A 7689,857 10,481 4,155E-024 0,000 Yes

Grand Mean vs. RpL6B 5641,551 7,689 4,561E-014 0,000 Yes

Grand Mean vs. RpS3 5310,526 7,238 1,111E-012 0,000 Yes

Grand Mean vs. RpS29A 3022,551 5,696 0,0000000175 0,000 Yes

Grand Mean vs. RpL40A 5056,699 5,671 0,0000000201 0,000 Yes

Grand Mean vs. RpS9A 2986,664 5,628 0,0000000256 0,000 Yes

Grand Mean vs. RpL36A 3785,462 5,159 0,000000316 0,000 Yes

Grand Mean vs. RpS8A 3750,018 5,111 0,000000405 0,000 Yes

Grand Mean vs. RpS21B 3479,968 4,743 0,00000251 0,000 Yes

Grand Mean vs. RpS1B 3328,903 4,537 0,00000662 0,000 Yes

Grand Mean vs. RpL25 3289,409 4,483 0,00000848 0,000 Yes

Grand Mean vs. RpL22A 3234,048 4,408 0,0000119 0,000 Yes

Grand Mean vs. RpS0A 3117,714 4,249 0,0000241 0,000 Yes

Grand Mean vs. RpL7A 3080,089 4,198 0,0000301 0,000 Yes

Grand Mean vs. RpL41A 2987,134 4,071 0,0000516 0,000 Yes

Grand Mean vs. RpL30 2975,633 4,056 0,0000551 0,000 Yes

Grand Mean vs. RpL13B 2899,479 3,952 0,0000848 0,000 Yes

Grand Mean vs. RpS13 2894,531 3,945 0,0000871 0,000 Yes

Grand Mean vs. RpP1A 3500,636 3,926 0,0000942 0,000 Yes

Grand Mean vs. RpS20 2865,591 3,906 0,000102 0,000 Yes

Grand Mean vs. RpS10B 2856,948 3,894 0,000107 0,001 Yes

Grand Mean vs. RpS25B 2837,713 3,868 0,000119 0,001 Yes

Grand Mean vs. RpS24B 2799,774 3,816 0,000147 0,001 Yes

Grand Mean vs. RpS16B 2707,581 3,690 0,000240 0,001 Yes

Grand Mean vs. RpL32 2641,773 3,601 0,000338 0,001 Yes

Grand Mean vs. RpL2B 2564,411 3,495 0,000501 0,001 Yes

Grand Mean vs. RpL34B 2437,498 3,322 0,000936 0,001 No

Grand Mean vs. RpS27B 2391,666 3,260 0,00116 0,001 No

Grand Mean vs. RpP1B 2346,204 3,198 0,00144 0,001 No

Grand Mean vs. RpL1B 2329,147 3,174 0,00156 0,001 No

Grand Mean vs. RpS28A 2319,571 3,161 0,00163 0,001 No

Grand Mean vs. RpL15A 2275,916 3,102 0,00199 0,001 No

Grand Mean vs. RpS7B 2270,948 3,095 0,00204 0,001 No

Grand Mean vs. RpL34A 2172,163 2,960 0,00317 0,001 No

Grand Mean vs. RpS19B 2161,873 2,946 0,00331 0,001 No

Grand Mean vs. RpS17A 2127,381 2,899 0,00385 0,001 No

Grand Mean vs. RpL33A 2118,043 2,887 0,00400 0,001 No

Grand Mean vs. RpS22A 2105,828 2,870 0,00422 0,001 No

Grand Mean vs. RpS6B 2097,558 2,859 0,00437 0,001 No

Grand Mean vs. RpL16B 2090,011 2,849 0,00451 0,001 No

Grand Mean vs. RpL19B 1450,914 2,734 0,00640 0,001 No

Grand Mean vs. RpL24B 1996,406 2,721 0,00666 0,001 No

Grand Mean vs. RpL21A 1960,216 2,672 0,00771 0,001 No

Grand Mean vs. RpS14A 1949,144 2,657 0,00806 0,001 No

Grand Mean vs. RpL12A 2339,806 2,624 0,00886 0,001 No

Grand Mean vs. RpS11B 1877,401 2,559 0,0107 0,001 No

Grand Mean vs. RpS16A 1798,864 2,452 0,0144 0,001 No

Grand Mean vs. RpL14A 1960,658 2,449 0,0146 0,001 No

Grand Mean vs. RpL6A 1787,556 2,436 0,0151 0,001 No

Grand Mean vs. RpS12 1862,176 2,326 0,0203 0,001 No

Grand Mean vs. RpS9B 1227,657 2,313 0,0210 0,001 No

Grand Mean vs. RpS5 1682,272 2,293 0,0221 0,001 No

Grand Mean vs. RpL12B 1678,594 2,288 0,0224 0,001 No

Grand Mean vs. RpS15 1663,351 2,267 0,0237 0,001 No

Grand Mean vs. RpS23B 1638,186 2,233 0,0259 0,001 No

Grand Mean vs. RpS1A 1612,148 2,197 0,0283 0,001 No

Grand Mean vs. RpS23A 1583,707 2,158 0,0312 0,001 No

Grand Mean vs. RpL38 1568,817 2,138 0,0328 0,001 No

Grand Mean vs. RpL24A 1652,128 2,063 0,0394 0,001 No

Grand Mean vs. RpL4A 1091,618 2,057 0,0400 0,001 No

Grand Mean vs. RpS4A 1426,391 1,944 0,0523 0,001 No

Grand Mean vs. RpL26A 1346,609 1,835 0,0668 0,001 No

Grand Mean vs. RpL9A 1344,186 1,832 0,0673 0,001 No

Grand Mean vs. RpL29 1539,807 1,727 0,0846 0,001 No

Grand Mean vs. RpL23B 1377,534 1,721 0,0857 0,001 No

Grand Mean vs. RpS18A 1235,002 1,683 0,0927 0,001 No

Grand Mean vs. RpL18A 1225,808 1,671 0,0952 0,001 No

Grand Mean vs. RpP2A 1210,827 1,650 0,0993 0,001 No

Grand Mean vs. RpS28B 1193,191 1,626 0,104 0,001 No

Grand Mean vs. RpS26B 1117,708 1,523 0,128 0,001 No

Grand Mean vs. RpL7B 1102,262 1,502 0,133 0,001 No

Grand Mean vs. RpL33B 1510,326 1,473 0,141 0,001 No

Grand Mean vs. RpL10 1045,544 1,425 0,155 0,001 No

Grand Mean vs. RpS11A 1040,497 1,418 0,157 0,001 No

Grand Mean vs. RpS2 745,316 1,405 0,161 0,001 No

Grand Mean vs. RpS29B 745,188 1,404 0,161 0,001 No

Grand Mean vs. RpL20A 1028,038 1,401 0,162 0,001 No

Grand Mean vs. RpL17A 1420,787 1,385 0,166 0,001 No

Grand Mean vs. RpL37A 976,344 1,331 0,184 0,001 No

Grand Mean vs. RpS21A 968,239 1,320 0,187 0,001 No

Grand Mean vs. RpS25A 949,001 1,293 0,196 0,001 No

Grand Mean vs. RpL21B 929,144 1,266 0,206 0,001 No

Grand Mean vs. RpP0 897,886 1,224 0,221 0,001 No

Grand Mean vs. RpS0B 953,896 1,191 0,234 0,001 No

Grand Mean vs. RpS14B 872,028 1,189 0,235 0,001 No

Grand Mean vs. RpL27B 934,574 1,167 0,243 0,001 No

Grand Mean vs. RpL41B 840,594 1,146 0,252 0,001 No

Grand Mean vs. RpL42A 817,354 1,114 0,266 0,001 No

Grand Mean vs. RpL26B 584,987 1,102 0,271 0,002 No

Grand Mean vs. RpL23A 805,524 1,098 0,273 0,002 No

Grand Mean vs. RpL8B 800,878 1,092 0,275 0,002 No

Grand Mean vs. RpL43B 754,869 1,029 0,304 0,002 No

Grand Mean vs. RpL35B 740,887 1,010 0,313 0,002 No

Grand Mean vs. RpL1A 735,002 1,002 0,317 0,002 No

Grand Mean vs. RpS31 708,431 0,966 0,335 0,002 No

Grand Mean vs. RpP2B 771,474 0,964 0,336 0,002 No

Grand Mean vs. RpL8A 700,839 0,955 0,340 0,002 No

Grand Mean vs. RpS27A 831,344 0,932 0,351 0,002 No

Grand Mean vs. RpS30A 678,011 0,924 0,356 0,002 No

Grand Mean vs. RpL18B 673,502 0,918 0,359 0,002 No

Grand Mean vs. RpS7A 656,911 0,895 0,371 0,002 No

Grand Mean vs. RpS4B 626,596 0,854 0,393 0,002 No

Grand Mean vs. RpL35A 614,449 0,837 0,403 0,003 No

Grand Mean vs. RpS22B 550,606 0,750 0,453 0,003 No

Grand Mean vs. RpS18B 536,848 0,732 0,465 0,003 No

Grand Mean vs. RpL28 460,236 0,627 0,531 0,003 No

Grand Mean vs. RpL22B 419,924 0,572 0,567 0,003 No

Grand Mean vs. RpS24A 396,246 0,540 0,589 0,003 No

Grand Mean vs. RpS19A 374,598 0,511 0,610 0,004 No

Grand Mean vs. RpL37B 239,812 0,327 0,744 0,004 No

Grand Mean vs. RpS6A 216,781 0,295 0,768 0,004 No

Grand Mean vs. RpL3 165,743 0,226 0,821 0,005 No

Grand Mean vs. RpL20B 162,268 0,221 0,825 0,005 No

Grand Mean vs. RpL40B 158,616 0,216 0,829 0,006 No

Grand Mean vs. RpS17B 146,089 0,199 0,842 0,006 No

Grand Mean vs. RpS10A 118,242 0,161 0,872 0,007 No

Grand Mean vs. RpL15B 61,558 0,0769 0,939 0,009 No

Grand Mean vs. RpL13A 64,012 0,0718 0,943 0,010 No

Grand Mean vs. RpL19A 50,233 0,0685 0,945 0,013 No

Grand Mean vs. RpS30B 48,824 0,0665 0,947 0,017 No

Grand Mean vs. RpL16A 25,166 0,0343 0,973 0,025 No

Grand Mean vs. RpL31A 3,101 0,00423 0,997 0,050 No
