# Supplementary material for: Specialized Yeast Ribosomes: A Customized Tool for Selective mRNA Translation
Source: PLoS One. 2013 Jul 8;8(7):e67609. doi: 10.1371/journal.pone.0067609 (PMC3704640; doi:10.1371/journal.pone.0067609)
Supplement: Table S6 — One way analysis of variance of LA3FF reporter readouts. (DOCX) [file pone.0067609.s007.docx]

**Supplementary Table S6:** One way analysis of variance of LA3FF reporter readouts.

**One Way Analysis of Variance**

**Data source:** LA3FF in ANOVAs.SNB

**Group Name N Missing Mean Std Dev SEM**

RpS0A 6 0 6514,013 1385,446 565,606

RpS0B 6 0 6731,992 1396,819 570,249

RpS1A 6 0 7049,067 1656,762 676,370

RpS1B 6 0 4964,513 2113,392 862,789

RpS2 12 0 7808,888 1720,282 496,603

RpS3 6 0 17796,233 2866,894 1170,405

RpS4A 5 0 25441,880 2310,515 1033,294

RpS4B 6 0 30424,133 2068,246 844,358

RpS5 5 0 25626,220 2251,726 1007,002

RpS6A 12 0 24888,433 2421,645 699,069

RpS6B 12 0 13503,914 2954,678 852,942

RpS7A 6 0 14831,567 2091,914 854,020

RpS7B 6 0 6774,858 1881,725 768,211

RpS8A 6 0 8288,745 1714,013 699,743

RpS9A 12 0 29577,333 4827,419 1393,556

RpS9B 12 0 8084,669 658,160 189,994

RpS10A 6 0 16279,300 711,293 290,384

RpS10B 5 0 13766,800 1862,011 832,717

RpS11A 6 0 10927,693 932,229 380,581

RpS11B 6 0 40229,600 13611,645 5556,931

RpS12 6 0 11945,417 1508,794 615,963

RpS13 6 0 5583,345 950,585 388,075

RpS14A 6 0 26648,983 2091,068 853,675

RpS14B 6 0 34424,167 6564,591 2679,983

RpS15 6 0 20692,167 1544,337 630,473

RpS16A 6 0 11831,070 1473,130 601,403

RpS16B 6 0 12805,398 1931,166 788,395

RpS17A 6 0 23854,050 1726,528 704,852

RpS17B 6 0 31441,567 2376,545 970,220

RpS18A 6 0 17014,433 2658,751 1085,431

RpS18B 6 0 13108,817 576,991 235,556

RpS19A 6 0 19076,900 2701,209 1102,764

RpS19B 6 0 15571,200 2065,876 843,390

RpS20 6 0 2719,528 920,397 375,751

RpS21A 6 0 8957,140 1904,733 777,604

RpS21B 6 0 5747,320 1124,667 459,143

RpS22A 6 0 7029,368 1533,365 625,994

RpS22B 6 0 28158,650 2671,849 1090,778

RpS23A 5 0 15200,820 438,121 195,934

RpS23B 6 0 8714,165 1993,504 813,845

RpS24A 6 0 12006,998 1747,883 713,570

RpS24B 6 0 9804,405 1867,653 762,466

RpS25A 6 0 29250,050 3641,705 1486,720

RpS25B 6 0 10088,095 1341,888 547,823

RpS26B 6 0 23581,450 4019,181 1640,824

RpS27A 6 0 29007,850 10032,560 4095,775

RpS27B 6 0 35474,483 6760,971 2760,155

RpS28A 6 0 19084,817 1509,489 616,246

RpS28B 6 0 29974,783 2009,717 820,464

RpS29A 12 0 15421,283 3010,149 868,955

RpS29B 12 0 9088,010 1314,238 379,388

RpS30A 6 0 18904,200 6185,976 2525,414

RpS30B 6 0 23715,833 1053,746 430,190

RpS31 4 0 13671,075 496,516 248,258

RpP0 6 0 16801,233 2625,702 1071,938

RpP1A 5 0 20761,960 4171,234 1865,433

RpP1B 6 0 26160,200 2449,585 1000,039

RpP2A 5 0 15952,500 881,164 394,069

RpP2B 6 0 25307,183 1927,964 787,088

RpL1A 6 0 23666,917 1295,336 528,819

RpL1B 6 0 16209,400 2582,197 1054,178

RpL2A 5 0 29322,860 2386,583 1067,312

RpL2B 5 0 21806,340 3418,254 1528,690

RpL3 6 0 16424,017 2985,821 1218,956

RpL4A 12 0 15439,008 1788,434 516,276

RpL6A 6 0 7748,215 705,478 288,010

RpL6B 5 0 10032,684 1296,304 579,725

RpL7A 6 0 13502,017 1316,597 537,498

RpL7B 6 0 10589,605 2815,502 1149,424

RpL8A 6 0 13454,467 2953,287 1205,674

RpL8B 5 0 14163,960 2171,179 970,981

RpL9A 6 0 13201,350 2554,197 1042,747

RpL10 6 0 16503,850 2812,768 1148,308

RpL11B 6 0 8875,070 1781,489 727,290

RpL12A 5 0 30989,140 1609,668 719,865

RpL12B 6 0 11665,075 1750,660 714,704

RpL13A 6 0 22670,100 7998,856 3265,519

RpL13B 3 0 15662,267 385,281 222,442

RpL14A 6 0 12399,900 1609,611 657,121

RpL15A 6 0 9375,783 2610,023 1065,537

RpL15B 6 0 13673,808 2871,622 1172,335

RpL16A 6 0 10201,573 1604,948 655,217

RpL16B 6 0 14722,213 4248,398 1734,401

RpL17A 5 0 10251,912 1456,252 651,256

RpL18A 6 0 18273,500 4647,902 1897,498

RpL18B 6 0 8516,182 1315,671 537,120

RpL19A 6 0 11541,667 2290,739 935,190

RpL19B 12 0 6180,413 859,146 248,014

RpL20A 6 0 8483,258 3654,600 1491,984

RpL20B 6 0 18963,100 1664,435 679,503

RpL21A 6 0 13752,783 1397,355 570,468

RpL21B 6 0 8085,415 3854,596 1573,632

RpL22A 6 0 6446,030 1948,990 795,672

RpL22B 6 0 14009,567 2607,603 1064,549

RpL23A 6 0 11166,952 2824,883 1153,254

RpL23B 6 0 11789,752 2905,800 1186,288

RpL24A 6 0 10031,807 3217,956 1313,725

RpL24B 6 0 9560,170 1941,429 792,585

RpL25 6 0 13361,815 2630,124 1073,744

RpL26A 6 0 9078,427 2813,978 1148,802

RpL26B 12 0 11514,662 2571,731 742,395

RpL27A 6 0 43019,933 3772,831 1540,252

RpL27B 6 0 21560,983 1871,807 764,162

RpL28 6 0 8785,080 1107,595 452,174

RpL29 6 0 11410,815 1945,497 794,246

RpL30 6 0 17414,833 4465,172 1822,899

RpL31A 6 0 37821,900 2865,988 1170,035

RpL32 6 0 9533,325 1347,556 550,137

RpL33A 5 0 12359,960 2566,148 1147,616

RpL33B 6 0 13830,933 2046,848 835,622

RpL34A 6 0 7503,398 800,895 326,964

RpL34B 6 0 9725,068 2098,465 856,695

RpL35A 6 0 16897,767 2385,204 973,755

RpL35B 6 0 12805,787 2040,521 833,039

RpL36A 6 0 15965,283 3499,744 1428,765

RpL37A 6 0 19260,500 1900,628 775,928

RpL37B 6 0 11209,483 2514,421 1026,508

RpL38 6 0 14720,783 3136,817 1280,600

RpL40A 6 0 8637,827 1497,955 611,538

RpL40B 6 0 10807,090 1359,480 555,005

RpL41A 6 0 13726,358 4028,943 1644,809

RpL41B 5 0 9027,170 577,348 258,198

RpL42A 6 0 19374,350 2450,736 1000,509

RpL43B 6 0 13053,867 1558,617 636,303

Grand Mean 124 0 15772,986 8104,191 727,778

**Source of Variation DF SS MS F P**

Between Groups 124 50987683121,056 411190992,912 23,108 <0,001

Residual 784 13950951278,038 17794580,712

Total 908 64938634399,094

The differences in the mean values among the treatment groups are greater than would be expected by chance; there is a statistically significant difference (P = <0,001).

Power of performed test with alpha = 0,050: 1,000

Multiple Comparisons versus Control Group (Holm-Sidak method):

Overall significance level = 0,05

Comparisons for factor:

**Comparison Diff of Means t Unadjusted P Critical Level Significant?**

Grand Mean vs. RpL27A 27246,947 15,452 3,220E-047 0,000 Yes

Grand Mean vs. RpS11B 24456,614 13,870 2,808E-039 0,000 Yes

Grand Mean vs. RpL31A 22048,914 12,504 7,633E-033 0,000 Yes

Grand Mean vs. RpS27B 19701,497 11,173 5,344E-027 0,000 Yes

Grand Mean vs. RpS9A 13804,347 10,824 1,521E-025 0,000 Yes

Grand Mean vs. RpS14B 18651,181 10,577 1,558E-024 0,000 Yes

Grand Mean vs. RpS17B 15668,581 8,886 4,289E-018 0,000 Yes

Grand Mean vs. RpS4B 14651,147 8,309 4,236E-016 0,000 Yes

Grand Mean vs. RpS28B 14201,797 8,054 2,973E-015 0,000 Yes

Grand Mean vs. RpL12A 15216,154 7,908 8,888E-015 0,000 Yes

Grand Mean vs. RpS25A 13477,064 7,643 6,201E-014 0,000 Yes

Grand Mean vs. RpL19B 9592,573 7,522 1,481E-013 0,000 Yes

Grand Mean vs. RpS27A 13234,864 7,506 1,662E-013 0,000 Yes

Grand Mean vs. RpS20 13053,458 7,403 3,443E-013 0,000 Yes

Grand Mean vs. RpS6A 9115,447 7,148 2,022E-012 0,000 Yes

Grand Mean vs. RpL2A 13549,874 7,042 4,148E-012 0,000 Yes

Grand Mean vs. RpS22B 12385,664 7,024 4,678E-012 0,000 Yes

Grand Mean vs. RpS2 7964,098 6,245 0,000000000695 0,000 Yes

Grand Mean vs. RpS14A 10875,997 6,168 0,00000000111 0,000 Yes

Grand Mean vs. RpS1B 10808,473 6,130 0,00000000139 0,000 Yes

Grand Mean vs. RpS9B 7688,317 6,029 0,00000000254 0,000 Yes

Grand Mean vs. RpP1B 10387,214 5,891 0,00000000570 0,000 Yes

Grand Mean vs. RpS13 10189,641 5,779 0,0000000109 0,001 Yes

Grand Mean vs. RpS21B 10025,666 5,686 0,0000000184 0,001 Yes

Grand Mean vs. RpP2B 9534,197 5,407 0,0000000851 0,001 Yes

Grand Mean vs. RpL22A 9326,956 5,289 0,000000159 0,001 Yes

Grand Mean vs. RpS0A 9258,973 5,251 0,000000195 0,001 Yes

Grand Mean vs. RpS29B 6684,976 5,242 0,000000205 0,001 Yes

Grand Mean vs. RpS0B 9040,994 5,127 0,000000371 0,001 Yes

Grand Mean vs. RpS5 9853,234 5,121 0,000000383 0,001 Yes

Grand Mean vs. RpS7B 8998,128 5,103 0,000000420 0,001 Yes

Grand Mean vs. RpS4A 9668,894 5,025 0,000000624 0,001 Yes

Grand Mean vs. RpS22A 8743,618 4,959 0,000000871 0,001 Yes

Grand Mean vs. RpS1A 8723,919 4,947 0,000000920 0,001 Yes

Grand Mean vs. RpL34A 8269,588 4,690 0,00000323 0,001 Yes

Grand Mean vs. RpS17A 8081,064 4,583 0,00000533 0,001 Yes

Grand Mean vs. RpL6A 8024,771 4,551 0,00000619 0,001 Yes

Grand Mean vs. RpS30B 7942,847 4,505 0,00000766 0,001 Yes

Grand Mean vs. RpL1A 7893,931 4,477 0,00000870 0,001 Yes

Grand Mean vs. RpS26B 7808,464 4,428 0,0000108 0,001 Yes

Grand Mean vs. RpL21B 7687,571 4,360 0,0000148 0,001 Yes

Grand Mean vs. RpS8A 7484,241 4,244 0,0000245 0,001 Yes

Grand Mean vs. RpL20A 7289,728 4,134 0,0000395 0,001 Yes

Grand Mean vs. RpL18B 7256,804 4,115 0,0000427 0,001 Yes

Grand Mean vs. RpL40A 7135,159 4,046 0,0000571 0,001 Yes

Grand Mean vs. RpS23B 7058,821 4,003 0,0000684 0,001 Yes

Grand Mean vs. RpL28 6987,906 3,963 0,0000808 0,001 Yes

Grand Mean vs. RpL11B 6897,916 3,912 0,0000995 0,001 Yes

Grand Mean vs. RpL13A 6897,114 3,911 0,0000997 0,001 Yes

Grand Mean vs. RpS21A 6815,846 3,865 0,000120 0,001 Yes

Grand Mean vs. RpL26A 6694,559 3,797 0,000158 0,001 Yes

Grand Mean vs. RpL15A 6397,203 3,628 0,000304 0,001 Yes

Grand Mean vs. RpL32 6239,661 3,539 0,000426 0,001 Yes

Grand Mean vs. RpL24B 6212,816 3,523 0,000451 0,001 Yes

Grand Mean vs. RpL41B 6745,816 3,506 0,000481 0,001 Yes

Grand Mean vs. RpL34B 6047,918 3,430 0,000635 0,001 Yes

Grand Mean vs. RpS24B 5968,581 3,385 0,000748 0,001 Yes

Grand Mean vs. RpL26B 4258,324 3,339 0,000880 0,001 No

Grand Mean vs. RpL27B 5787,997 3,282 0,00107 0,001 No

Grand Mean vs. RpL24A 5741,179 3,256 0,00118 0,001 No

Grand Mean vs. RpS25B 5684,891 3,224 0,00132 0,001 No

Grand Mean vs. RpL16A 5571,413 3,160 0,00164 0,001 No

Grand Mean vs. RpL2B 6033,354 3,136 0,00178 0,001 No

Grand Mean vs. RpL6B 5740,302 2,983 0,00294 0,001 No

Grand Mean vs. RpL7B 5183,381 2,940 0,00338 0,001 No

Grand Mean vs. RpL17A 5521,074 2,869 0,00422 0,001 No

Grand Mean vs. RpL40B 4965,896 2,816 0,00498 0,001 No

Grand Mean vs. RpS15 4919,181 2,790 0,00540 0,001 No

Grand Mean vs. RpS11A 4845,293 2,748 0,00614 0,001 No

Grand Mean vs. RpL23A 4606,034 2,612 0,00917 0,001 No

Grand Mean vs. RpP1A 4988,974 2,593 0,00970 0,001 No

Grand Mean vs. RpL37B 4563,503 2,588 0,00983 0,001 No

Grand Mean vs. RpL29 4362,171 2,474 0,0136 0,001 No

Grand Mean vs. RpL19A 4231,319 2,400 0,0166 0,001 No

Grand Mean vs. RpL12B 4107,911 2,330 0,0201 0,001 No

Grand Mean vs. RpL23B 3983,234 2,259 0,0242 0,001 No

Grand Mean vs. RpS16A 3941,916 2,236 0,0257 0,001 No

Grand Mean vs. RpS12 3827,569 2,171 0,0303 0,001 No

Grand Mean vs. RpS24A 3765,988 2,136 0,0330 0,001 No

Grand Mean vs. RpL42A 3601,364 2,042 0,0414 0,001 No

Grand Mean vs. RpL37A 3487,514 1,978 0,0483 0,001 No

Grand Mean vs. RpL14A 3373,086 1,913 0,0561 0,001 No

Grand Mean vs. RpS28A 3311,831 1,878 0,0607 0,001 No

Grand Mean vs. RpS19A 3303,914 1,874 0,0613 0,001 No

Grand Mean vs. RpL20B 3190,114 1,809 0,0708 0,001 No

Grand Mean vs. RpS6B 2269,072 1,779 0,0756 0,001 No

Grand Mean vs. RpS30A 3131,214 1,776 0,0762 0,001 No

Grand Mean vs. RpL33A 3413,026 1,774 0,0765 0,001 No

Grand Mean vs. RpS16B 2967,588 1,683 0,0928 0,001 No

Grand Mean vs. RpL35B 2967,199 1,683 0,0928 0,001 No

Grand Mean vs. RpL43B 2719,119 1,542 0,123 0,002 No

Grand Mean vs. RpS18B 2664,169 1,511 0,131 0,002 No

Grand Mean vs. RpL9A 2571,636 1,458 0,145 0,002 No

Grand Mean vs. RpL18A 2500,514 1,418 0,157 0,002 No

Grand Mean vs. RpL25 2411,171 1,367 0,172 0,002 No

Grand Mean vs. RpL8A 2318,519 1,315 0,189 0,002 No

Grand Mean vs. RpL7A 2270,969 1,288 0,198 0,002 No

Grand Mean vs. RpL15B 2099,178 1,190 0,234 0,002 No

Grand Mean vs. RpL41A 2046,628 1,161 0,246 0,002 No

Grand Mean vs. RpS3 2023,247 1,147 0,252 0,002 No

Grand Mean vs. RpL21A 2020,203 1,146 0,252 0,002 No

Grand Mean vs. RpL33B 1942,053 1,101 0,271 0,002 No

Grand Mean vs. RpS10B 2006,186 1,043 0,297 0,002 No

Grand Mean vs. RpL22B 1763,419 1,000 0,318 0,002 No

Grand Mean vs. RpS31 2101,911 0,981 0,327 0,003 No

Grand Mean vs. RpL30 1641,847 0,931 0,352 0,003 No

Grand Mean vs. RpL8B 1609,026 0,836 0,403 0,003 No

Grand Mean vs. RpS18A 1241,447 0,704 0,482 0,003 No

Grand Mean vs. RpL35A 1124,781 0,638 0,524 0,003 No

Grand Mean vs. RpL38 1052,203 0,597 0,551 0,003 No

Grand Mean vs. RpL16B 1050,773 0,596 0,551 0,004 No

Grand Mean vs. RpP0 1028,247 0,583 0,560 0,004 No

Grand Mean vs. RpS7A 941,419 0,534 0,594 0,004 No

Grand Mean vs. RpL10 730,864 0,414 0,679 0,005 No

Grand Mean vs. RpL3 651,031 0,369 0,712 0,005 No

Grand Mean vs. RpS23A 572,166 0,297 0,766 0,006 No

Grand Mean vs. RpS10A 506,314 0,287 0,774 0,006 No

Grand Mean vs. RpS29A 351,703 0,276 0,783 0,007 No

Grand Mean vs. RpL4A 333,978 0,262 0,793 0,009 No

Grand Mean vs. RpL1B 436,414 0,247 0,805 0,010 No

Grand Mean vs. RpS19B 201,786 0,114 0,909 0,013 No

Grand Mean vs. RpL36A 192,297 0,109 0,913 0,017 No

Grand Mean vs. RpP2A 179,514 0,0933 0,926 0,025 No

Grand Mean vs. RpL13B 110,719 0,0449 0,964 0,050 No
