# Supplementary material for: Specialized Yeast Ribosomes: A Customized Tool for Selective mRNA Translation
Source: PLoS One. 2013 Jul 8;8(7):e67609. doi: 10.1371/journal.pone.0067609 (PMC3704640; doi:10.1371/journal.pone.0067609)
Supplement: Table S7 — One way analysis of variance of LA3PTCFF reporter readouts. (DOCX) [file pone.0067609.s008.docx]

**Supplementary Table S7:** One way analysis of variance of LA3PTCFF reporter readouts.

**One Way Analysis of Variance**

**Data source:** LA3PTCFF in ANOVAs.SNB

**Group Name N Missing Mean Std Dev SEM**

RpS0A 6 0 1947,298 719,719 293,824

RpS0B 6 0 4699,148 935,853 382,060

RpS1A 6 0 3132,045 826,394 337,374

RpS1B 5 0 1559,104 109,760 49,086

RpS2 12 0 3566,270 985,209 284,405

RpS3 6 0 7175,050 1472,010 600,946

RpS4A 6 0 5801,783 1671,695 682,467

RpS4B 6 0 4706,960 1500,694 612,656

RpS5 6 0 3446,320 860,050 351,114

RpS6A 6 0 4909,555 1460,168 596,111

RpS6B 6 0 2205,242 468,979 191,460

RpS7A 5 0 2589,482 192,612 86,139

RpS7B 6 0 1752,893 300,540 122,695

RpS8A 6 0 2871,288 1537,621 627,731

RpS9A 12 0 4833,961 1417,194 409,109

RpS9B 12 0 2791,792 499,852 144,295

RpS10A 6 0 8011,390 2500,645 1020,884

RpS10B 6 0 3090,367 1751,199 714,924

RpS11A 6 0 7889,037 852,998 348,235

RpS11B 6 0 4504,858 1307,172 533,651

RpS12 6 0 7580,233 704,093 287,445

RpS13 6 0 3462,077 849,888 346,965

RpS14A 6 0 3074,332 1077,651 439,949

RpS14B 6 0 3316,772 1351,056 551,566

RpS15 6 0 3481,712 812,565 331,728

RpS16A 6 0 3802,848 1005,632 410,548

RpS16B 6 0 3687,450 745,871 304,501

RpS17A 6 0 2200,038 991,558 404,802

RpS17B 6 0 4363,070 2502,348 1021,579

RpS18A 5 0 7416,612 2214,956 990,558

RpS18B 5 0 4283,014 652,165 291,657

RpS19A 6 0 6980,968 883,410 360,651

RpS19B 6 0 2658,152 985,329 402,259

RpS20 6 0 3491,300 1487,486 607,264

RpS21A 6 0 3192,665 1012,007 413,150

RpS21B 6 0 2464,453 811,922 331,466

RpS22A 6 0 2771,647 1586,660 647,751

RpS22B 6 0 6609,278 1936,510 790,577

RpS23A 4 0 4187,560 652,637 326,318

RpS23B 6 0 4606,428 827,010 337,625

RpS24A 6 0 3250,678 745,959 304,536

RpS24B 4 0 1870,325 748,735 374,368

RpS25A 6 0 2045,153 402,657 164,384

RpS25B 6 0 3505,703 806,978 329,447

RpS26B 6 0 4398,512 1933,042 789,161

RpS27A 6 0 4385,740 1321,719 539,590

RpS27B 6 0 4497,300 1285,678 524,876

RpS28A 6 0 5310,305 1088,650 444,440

RpS28B 6 0 2974,717 600,505 245,155

RpS29A 12 0 4892,539 1200,038 346,421

RpS29B 10 0 3829,728 939,308 297,035

RpS30A 6 0 4112,780 1412,300 576,569

RpS30B 5 0 5700,612 1545,069 690,976

RpS31 6 0 2811,778 437,624 178,659

RpP0 6 0 2475,888 792,094 323,371

RpP1A 5 0 5985,628 1248,363 558,285

RpP1B 6 0 6935,507 1554,747 634,723

RpP2A 6 0 5312,445 1305,839 533,107

RpP2B 6 0 5763,990 2325,024 949,187

RpL1A 6 0 2569,863 991,928 404,953

RpL1B 6 0 4617,203 1241,107 506,680

RpL2A 5 0 16810,360 1950,786 872,418

RpL2B 5 0 3361,142 324,931 145,314

RpL3 6 0 3448,085 1225,537 500,323

RpL4A 12 0 4812,922 951,118 274,564

RpL6A 6 0 3806,412 1448,515 591,354

RpL6B 6 0 3996,910 1499,139 612,021

RpL7A 6 0 4140,668 2322,275 948,065

RpL7B 6 0 4942,088 765,749 312,616

RpL8A 6 0 3136,748 1749,919 714,401

RpL8B 6 0 2831,488 578,230 236,061

RpL9A 6 0 2193,630 643,060 262,528

RpL10 6 0 4515,110 925,809 377,960

RpL11B 6 0 5860,705 672,693 274,626

RpL12A 6 0 1651,712 381,353 155,687

RpL12B 6 0 3038,663 777,820 317,544

RpL13A 6 0 6625,762 2549,911 1040,997

RpL13B 6 0 2073,510 753,109 307,455

RpL14A 6 0 5018,417 1245,963 508,662

RpL15A 6 0 2917,168 696,648 284,405

RpL15B 6 0 4132,573 1902,087 776,524

RpL16A 6 0 4192,860 1059,334 432,471

RpL16B 6 0 7508,523 2357,759 962,551

RpL17A 6 0 6422,488 2396,970 978,559

RpL18A 6 0 3696,868 1293,901 528,233

RpL18B 5 0 5134,306 985,889 440,903

RpL19A 6 0 3513,130 657,425 268,393

RpL19B 10 0 2333,898 502,754 158,985

RpL20A 5 0 4194,600 163,419 73,083

RpL20B 6 0 4755,382 1062,753 433,867

RpL21A 6 0 3058,727 611,234 249,535

RpL21B 6 0 3788,100 1277,738 521,634

RpL22A 6 0 1826,798 820,011 334,768

RpL22B 6 0 3241,107 727,427 296,971

RpL23A 6 0 3234,183 903,883 369,009

RpL23B 6 0 3495,553 1042,633 425,653

RpL24A 4 0 6252,497 281,384 140,692

RpL24B 5 0 5070,596 330,208 147,674

RpL25 6 0 2586,220 551,545 225,167

RpL26A 6 0 2565,898 504,118 205,805

RpL26B 12 0 5170,284 1862,283 537,595

RpL27A 6 0 15024,650 2627,259 1072,574

RpL27B 3 0 4634,600 238,810 137,877

RpL28 6 0 5405,193 1285,771 524,914

RpL29 6 0 5748,537 199,890 81,605

RpL30 5 0 1244,807 470,389 210,364

RpL31A 6 0 3983,858 1516,808 619,234

RpL32 6 0 2745,115 562,024 229,445

RpL33A 6 0 2223,242 760,417 310,439

RpL33B 6 0 2670,123 468,959 191,452

RpL34A 6 0 3318,815 933,142 380,954

RpL34B 6 0 2139,997 541,408 221,029

RpL35A 6 0 2889,212 1672,755 682,899

RpL35B 6 0 8444,313 3023,124 1234,185

RpL36A 6 0 4451,697 1901,716 776,372

RpL37A 6 0 6605,233 1838,168 750,429

RpL37B 6 0 5020,227 1713,823 699,665

RpL38 6 0 6461,610 1229,354 501,882

RpL40A 6 0 6168,613 2771,782 1131,575

RpL40B 6 0 7537,355 1825,754 745,361

RpL41A 6 0 6717,377 1168,335 476,971

RpL41B 6 0 5239,272 1121,693 457,929

RpL42A 0 0 4367,597 3443,956 (+inf)

RpL43B 6 0 2777,707 1208,374 493,317

Grand Mean 124 0 4351,097 2197,995 197,386

**Source of Variation DF SS MS F P**

Between Groups 124 3397380641,684 27398230,981 12,547 <0,001

Residual 760 1659610225,359 2183697,665

Total 884 5056990867,043

The differences in the mean values among the treatment groups are greater than would be expected by chance; there is a statistically significant difference (P = <0,001).

Power of performed test with alpha = 0,050: 1,000

Multiple Comparisons versus Control Group (Holm-Sidak method):

Overall significance level = 0,05

Comparisons for factor:

**Comparison Diff of Means t Unadjusted P Critical Level Significant?**

Grand Mean vs. RpL2A 12459,263 18,484 2,789E-063 0,000 Yes

Grand Mean vs. RpL27A 10673,553 17,279 1,121E-056 0,000 Yes

Grand Mean vs. RpL35B 4093,216 6,626 6,511E-011 0,000 Yes

Grand Mean vs. RpS10A 3660,293 5,926 0,00000000472 0,000 Yes

Grand Mean vs. RpS11A 3537,940 5,728 0,0000000147 0,000 Yes

Grand Mean vs. RpS12 3229,136 5,228 0,000000222 0,000 Yes

Grand Mean vs. RpL40B 3186,258 5,158 0,000000318 0,000 Yes

Grand Mean vs. RpL16B 3157,426 5,112 0,000000405 0,000 Yes

Grand Mean vs. RpL30 3106,290 4,608 0,00000476 0,000 Yes

Grand Mean vs. RpS3 2823,953 4,572 0,00000565 0,000 Yes

Grand Mean vs. RpS18A 3065,515 4,548 0,00000630 0,000 Yes

Grand Mean vs. RpL12A 2699,385 4,370 0,0000142 0,000 Yes

Grand Mean vs. RpS19A 2629,871 4,257 0,0000233 0,000 Yes

Grand Mean vs. RpS7B 2598,204 4,206 0,0000291 0,000 Yes

Grand Mean vs. RpP1B 2584,410 4,184 0,0000320 0,000 Yes

Grand Mean vs. RpL19B 2017,199 4,153 0,0000366 0,000 Yes

Grand Mean vs. RpS1B 2791,993 4,142 0,0000383 0,000 Yes

Grand Mean vs. RpL22A 2524,299 4,087 0,0000484 0,000 Yes

Grand Mean vs. RpS0A 2403,799 3,891 0,000108 0,000 Yes

Grand Mean vs. RpL41A 2366,280 3,831 0,000138 0,000 Yes

Grand Mean vs. RpS25A 2305,944 3,733 0,000203 0,000 Yes

Grand Mean vs. RpL13B 2277,587 3,687 0,000243 0,001 Yes

Grand Mean vs. RpL13A 2274,665 3,682 0,000247 0,001 Yes

Grand Mean vs. RpS22B 2258,181 3,656 0,000274 0,001 Yes

Grand Mean vs. RpL37A 2254,136 3,649 0,000281 0,001 Yes

Grand Mean vs. RpL34B 2211,100 3,580 0,000366 0,001 Yes

Grand Mean vs. RpL9A 2157,467 3,493 0,000506 0,001 Yes

Grand Mean vs. RpS9B 1559,305 3,490 0,000510 0,001 Yes

Grand Mean vs. RpS17A 2151,059 3,482 0,000525 0,001 Yes

Grand Mean vs. RpS6B 2145,855 3,474 0,000542 0,001 Yes

Grand Mean vs. RpL33A 2127,855 3,445 0,000603 0,001 No

Grand Mean vs. RpL38 2110,513 3,417 0,000668 0,001 No

Grand Mean vs. RpL17A 2071,391 3,353 0,000838 0,001 No

Grand Mean vs. RpS24B 2480,772 3,305 0,000995 0,001 No

Grand Mean vs. RpS21B 1886,644 3,054 0,00233 0,001 No

Grand Mean vs. RpP0 1875,209 3,036 0,00248 0,001 No

Grand Mean vs. RpL40A 1817,516 2,942 0,00336 0,001 No

Grand Mean vs. RpL26A 1785,199 2,890 0,00396 0,001 No

Grand Mean vs. RpL1A 1781,234 2,884 0,00404 0,001 No

Grand Mean vs. RpL25 1764,877 2,857 0,00439 0,001 No

Grand Mean vs. RpS19B 1692,945 2,741 0,00627 0,001 No

Grand Mean vs. RpL33B 1680,974 2,721 0,00665 0,001 No

Grand Mean vs. RpS7A 1761,615 2,613 0,00914 0,001 No

Grand Mean vs. RpL32 1605,982 2,600 0,00951 0,001 No

Grand Mean vs. RpS22A 1579,450 2,557 0,0108 0,001 No

Grand Mean vs. RpL43B 1573,390 2,547 0,0111 0,001 No

Grand Mean vs. RpL24A 1901,400 2,533 0,0115 0,001 No

Grand Mean vs. RpS31 1539,319 2,492 0,0129 0,001 No

Grand Mean vs. RpL8B 1519,609 2,460 0,0141 0,001 No

Grand Mean vs. RpL11B 1509,608 2,444 0,0148 0,001 No

Grand Mean vs. RpP1A 1634,531 2,425 0,0155 0,001 No

Grand Mean vs. RpS8A 1479,809 2,396 0,0168 0,001 No

Grand Mean vs. RpL35A 1461,885 2,367 0,0182 0,001 No

Grand Mean vs. RpS4A 1450,686 2,349 0,0191 0,001 No

Grand Mean vs. RpL15A 1433,929 2,321 0,0205 0,001 No

Grand Mean vs. RpP2B 1412,893 2,287 0,0225 0,001 No

Grand Mean vs. RpL29 1397,440 2,262 0,0240 0,001 No

Grand Mean vs. RpS28B 1376,380 2,228 0,0262 0,001 No

Grand Mean vs. RpL12B 1312,434 2,125 0,0339 0,001 No

Grand Mean vs. RpL21A 1292,370 2,092 0,0368 0,001 No

Grand Mean vs. RpS14A 1276,765 2,067 0,0391 0,001 No

Grand Mean vs. RpS10B 1260,730 2,041 0,0416 0,001 No

Grand Mean vs. RpS30B 1349,515 2,002 0,0456 0,001 No

Grand Mean vs. RpS1A 1219,052 1,974 0,0488 0,001 No

Grand Mean vs. RpL8A 1214,349 1,966 0,0497 0,001 No

Grand Mean vs. RpS21A 1158,432 1,875 0,0611 0,001 No

Grand Mean vs. RpL26B 819,187 1,834 0,0671 0,001 No

Grand Mean vs. RpL23A 1116,914 1,808 0,0710 0,001 No

Grand Mean vs. RpL22B 1109,990 1,797 0,0727 0,001 No

Grand Mean vs. RpS24A 1100,419 1,781 0,0752 0,001 No

Grand Mean vs. RpS2 784,827 1,757 0,0794 0,001 No

Grand Mean vs. RpL28 1054,096 1,706 0,0883 0,001 No

Grand Mean vs. RpS14B 1034,325 1,674 0,0945 0,001 No

Grand Mean vs. RpL34A 1032,282 1,671 0,0951 0,001 No

Grand Mean vs. RpP2A 961,348 1,556 0,120 0,001 No

Grand Mean vs. RpS28A 959,208 1,553 0,121 0,001 No

Grand Mean vs. RpL2B 989,955 1,469 0,142 0,001 No

Grand Mean vs. RpS5 904,777 1,465 0,143 0,001 No

Grand Mean vs. RpL3 903,012 1,462 0,144 0,001 No

Grand Mean vs. RpS13 889,020 1,439 0,150 0,001 No

Grand Mean vs. RpL41B 888,175 1,438 0,151 0,001 No

Grand Mean vs. RpS15 869,385 1,407 0,160 0,001 No

Grand Mean vs. RpS20 859,797 1,392 0,164 0,001 No

Grand Mean vs. RpL23B 855,544 1,385 0,166 0,001 No

Grand Mean vs. RpS25B 845,394 1,369 0,172 0,001 No

Grand Mean vs. RpL19A 837,967 1,357 0,175 0,001 No

Grand Mean vs. RpS29A 541,442 1,212 0,226 0,001 No

Grand Mean vs. RpL18B 783,209 1,162 0,246 0,001 No

Grand Mean vs. RpL37B 669,130 1,083 0,279 0,001 No

Grand Mean vs. RpS9A 482,864 1,081 0,280 0,002 No

Grand Mean vs. RpL14A 667,320 1,080 0,280 0,002 No

Grand Mean vs. RpS16B 663,647 1,074 0,283 0,002 No

Grand Mean vs. RpS29B 521,369 1,073 0,283 0,002 No

Grand Mean vs. RpL24B 719,499 1,067 0,286 0,002 No

Grand Mean vs. RpL18A 654,229 1,059 0,290 0,002 No

Grand Mean vs. RpL4A 461,825 1,034 0,302 0,002 No

Grand Mean vs. RpL7B 590,991 0,957 0,339 0,002 No

Grand Mean vs. RpL21B 562,997 0,911 0,362 0,002 No

Grand Mean vs. RpS6A 558,458 0,904 0,366 0,002 No

Grand Mean vs. RpS16A 548,249 0,888 0,375 0,002 No

Grand Mean vs. RpL6A 544,685 0,882 0,378 0,002 No

Grand Mean vs. RpL20B 404,285 0,654 0,513 0,002 No

Grand Mean vs. RpL31A 367,239 0,595 0,552 0,002 No

Grand Mean vs. RpS4B 355,863 0,576 0,565 0,003 No

Grand Mean vs. RpL6B 354,187 0,573 0,567 0,003 No

Grand Mean vs. RpS0B 348,051 0,563 0,573 0,003 No

Grand Mean vs. RpL1B 266,106 0,431 0,667 0,003 No

Grand Mean vs. RpS23B 255,331 0,413 0,679 0,003 No

Grand Mean vs. RpS30A 238,317 0,386 0,700 0,003 No

Grand Mean vs. RpL15B 218,524 0,354 0,724 0,004 No

Grand Mean vs. RpL7A 210,429 0,341 0,733 0,004 No

Grand Mean vs. RpL27B 283,503 0,328 0,743 0,004 No

Grand Mean vs. RpL10 164,013 0,266 0,791 0,005 No

Grand Mean vs. RpL16A 158,237 0,256 0,798 0,005 No

Grand Mean vs. RpS11B 153,761 0,249 0,803 0,006 No

Grand Mean vs. RpS27B 146,203 0,237 0,813 0,006 No

Grand Mean vs. RpL20A 156,497 0,232 0,816 0,007 No

Grand Mean vs. RpS23A 163,537 0,218 0,828 0,009 No

Grand Mean vs. RpL36A 100,600 0,163 0,871 0,010 No

Grand Mean vs. RpS18B 68,083 0,101 0,920 0,013 No

Grand Mean vs. RpS26B 47,415 0,0768 0,939 0,017 No

Grand Mean vs. RpS27A 34,643 0,0561 0,955 0,025 No

Grand Mean vs. RpS17B 11,973 0,0194 0,985 0,050 No
