# Supplementary material for: Specialized Yeast Ribosomes: A Customized Tool for Selective mRNA Translation
Source: PLoS One. 2013 Jul 8;8(7):e67609. doi: 10.1371/journal.pone.0067609 (PMC3704640; doi:10.1371/journal.pone.0067609)
Supplement: Table S8 — One way analysis of variance of REN[FF] reporter readouts. (DOCX) [file pone.0067609.s009.docx]

**Supplementary Table S8:** One way analysis of variance of REN[FF] reporter readouts.

**One Way Analysis of Variance**

**Data source:** REN[FF] in ANOVAs.SNB

**Group Name N Missing Mean Std Dev SEM**

RpS0A 6 0 207340166,700 11419557,850 4662014,970

RpS0B 6 0 166858166,700 10068872,990 4110600,185

RpS1A 6 0 120009166,700 5475838,179 2235501,575

RpS1B 6 0 135961833,300 6751062,329 2756109,655

RpS2 11 0 134273000,000 5460391,524 1646369,990

RpS3 6 0 156789500,000 2634002,107 1075326,857

RpS4A 4 0 91565825,000 4403135,057 2201567,529

RpS4B 6 0 131988666,667 4775333,063 1949521,559

RpS5 6 0 245604666,667 16722060,886 6826752,770

RpS6A 12 0 167942083,333 17034781,294 4917517,783

RpS6B 12 0 146436333,333 10897687,531 3145891,415

RpS7A 6 0 57341600,000 5386881,036 2199184,974

RpS7B 6 0 207646333,333 19879163,782 8115634,630

RpS8A 6 0 214140333,333 16787419,464 6853435,297

RpS9A 12 0 162675250,000 7336909,507 2117983,339

RpS9B 12 0 115031083,333 5425136,370 1566101,972

RpS10A 6 0 79371366,667 3142964,722 1283109,975

RpS10B 6 0 110326333,333 7101796,806 2899296,405

RpS11A 6 0 113599000,000 5515675,734 2251765,189

RpS11B 6 0 154514166,667 4984991,531 2035114,271

RpS12 6 0 128638333,333 3904445,808 1593983,326

RpS13 5 0 190876800,000 692581,548 309731,884

RpS14A 6 0 188144666,667 12880279,526 5258352,097

RpS14B 6 0 156464000,000 16316735,115 6661279,217

RpS15 6 0 247241666,667 16416849,535 6702150,757

RpS16A 6 0 145968833,333 8778280,912 3583718,176

RpS16B 6 0 171177500,000 8179410,633 3339230,408

RpS17A 6 0 152226333,333 18367560,041 7498524,987

RpS17B 6 0 169659166,667 6631263,030 2707201,796

RpS18A 6 0 137879166,667 18641088,921 7610192,684

RpS18B 6 0 140224666,667 7626460,507 3113489,464

RpS19A 6 0 247246833,333 26908581,058 10985382,216

RpS19B 6 0 116780666,667 3889643,771 1587940,420

RpS20 6 0 95588533,333 9729346,707 3971989,160

RpS21A 6 0 249636500,000 27905386,195 11392326,209

RpS21B 6 0 243124500,000 19310291,658 7883393,558

RpS22A 6 0 175141000,000 7158945,677 2922627,334

RpS22B 6 0 259544166,667 14233643,138 5810860,478

RpS23A 6 0 153359166,667 12040197,480 4915390,038

RpS23B 3 0 169465000,000 11096939,894 6406821,235

RpS24A 6 0 150572500,000 6693504,635 2732611,824

RpS24B 6 0 225638333,333 15380591,207 6279100,067

RpS25A 6 0 166054666,667 12040898,499 4915676,228

RpS25B 6 0 177181833,333 4950224,779 2020920,803

RpS26B 5 0 186970000,000 28002547,429 12523119,919

RpS27A 6 0 171158333,333 7039298,535 2873781,593

RpS27B 6 0 226863333,333 10957986,981 4473579,452

RpS28A 6 0 134164000,000 6138828,162 2506166,103

RpS28B 6 0 201296000,000 11254845,570 4594771,463

RpS29A 10 0 182167700,000 4445670,304 1405844,389

RpS29B 12 0 95268583,333 4390590,297 1267454,245

RpS30A 6 0 195278666,667 13329994,144 5441947,321

RpS30B 4 0 250559500,000 1783447,691 891723,846

RpS31 6 0 194769333,333 13911094,632 5679180,602

RpP0 6 0 282520166,667 10981843,733 4483318,930

RpP1A 6 0 172839166,667 17338682,089 7078487,322

RpP1B 6 0 202544000,000 15962240,169 6516557,261

RpP2A 6 0 225322333,333 8077098,406 3297461,616

RpP2B 6 0 318146666,667 25480331,965 10402301,965

RpL1A 6 0 167026500,000 8340443,292 3404971,716

RpL1B 6 0 223244166,667 18112337,314 7394330,745

RpL2A 6 0 126413000,000 4202877,633 1715817,609

RpL2B 6 0 220427166,667 18417372,325 7518860,767

RpL3 5 0 222267600,000 8296370,128 3710249,515

RpL4A 12 0 182414000,000 12195617,350 3520571,480

RpL6A 6 0 163160500,000 6993827,328 2855218,050

RpL6B 6 0 189899833,333 9745022,369 3978388,723

RpL7A 6 0 150459333,333 2609746,246 1065424,443

RpL7B 6 0 404594333,333 34072454,667 13910021,370

RpL8A 6 0 217898500,000 10165392,934 4150004,287

RpL8B 6 0 230877333,333 7075475,946 2888550,959

RpL9A 6 0 143028666,667 2572868,645 1050369,226

RpL10 6 0 178828166,667 5597771,893 2285280,806

RpL11B 6 0 221446333,333 25292150,883 10325477,360

RpL12A 3 0 157635333,333 15290384,310 8827907,497

RpL12B 6 0 244974666,667 11942401,997 4875465,199

RpL13A 6 0 65062983,333 22356176,085 9126870,668

RpL13B 6 0 183634833,333 8161943,063 3332099,302

RpL14A 6 0 166508666,667 3815816,383 1557800,515

RpL15A 4 0 310295250,000 16240238,758 8120119,379

RpL15B 6 0 214409333,333 9260390,762 3780538,698

RpL16A 6 0 191865000,000 7687865,113 3138557,790

RpL16B 6 0 305999333,333 11705467,828 4778737,230

RpL17A 6 0 213506500,000 7328926,272 2992021,621

RpL18A 6 0 178584333,333 10886238,261 4444288,160

RpL18B 3 0 172912000,000 2266656,569 1308654,780

RpL19A 6 0 261086166,667 14403993,188 5880405,595

RpL19B 12 0 91513150,000 4941448,367 1426473,272

RpL20A 6 0 306844833,333 22844448,108 9326206,887

RpL20B 6 0 202794666,667 9070982,894 3703213,259

RpL21A 6 0 173055833,333 7822938,972 3193701,462

RpL21B 6 0 257093333,333 16219534,897 6621597,394

RpL22A 5 0 188593600,000 10974859,944 4908106,576

RpL22B 6 0 173293666,667 4301399,571 1756039,021

RpL23A 6 0 321491333,333 19437033,474 7935135,687

RpL23B 6 0 178050666,667 6770865,164 2764194,128

RpL24A 6 0 218139833,333 10147134,047 4142550,128

RpL24B 6 0 218122833,333 11387601,809 4648968,971

RpL25 6 0 189395000,000 16267958,704 6641366,330

RpL26A 6 0 263489500,000 22002306,177 8982403,883

RpL26B 12 0 140374166,667 10158113,352 2932394,739

RpL27A 6 0 148786000,000 10213706,575 4169728,249

RpL27B 6 0 239470000,000 6401410,532 2613364,906

RpL28 6 0 162731833,333 5038052,418 2056776,287

RpL29 4 0 236561250,000 14172808,035 7086404,018

RpL30 6 0 99827116,667 4249366,978 1734796,804

RpL31A 6 0 156070000,000 18773836,965 7664386,846

RpL32 6 0 225775833,333 11113998,712 4537270,974

RpL33A 6 0 112023000,000 6669277,922 2722721,310

RpL33B 6 0 140771833,333 12246296,345 4999529,547

RpL34A 6 0 125743833,333 5667482,736 2313740,138

RpL34B 6 0 115959500,000 3582646,885 1462609,466

RpL35A 6 0 196536666,667 7820738,345 3192803,060

RpL35B 6 0 235513000,000 14544558,969 5937791,335

RpL36A 5 0 208827000,000 2564531,829 1146893,500

RpL37A 6 0 178520166,667 10016932,553 4089395,590

RpL37B 6 0 146281166,667 11797253,111 4816208,415

RpL38 3 0 177192333,333 5291131,196 3054836,020

RpL40A 6 0 390416333,333 20362151,956 8312813,726

RpL40B 6 0 155765166,667 4590053,351 1873881,434

RpL41A 4 0 267857000,000 1276322,843 638161,422

RpL41B 6 0 213175833,333 10845768,768 4427766,558

RpL42A 6 0 193758500,000 7187206,098 2934164,603

RpL43B 6 0 155273666,667 17277247,196 7053406,632

Grand Mean 124 0 186537635,283 59696301,489 5360886,129

**Source of Variation DF SS MS F P**

Between Groups 124 2,716E+018 2,190E+016 31,441 <0,001

Residual 773 5,385E+017 6,966E+014

Total 897 3,254E+018

The differences in the mean values among the treatment groups are greater than would be expected by chance; there is a statistically significant difference (P = <0,001).

Power of performed test with alpha = 0,050: 1,000

Multiple Comparisons versus Control Group (Holm-Sidak method):

Overall significance level = 0,05

Comparisons for factor:

**Comparison Diff of Means t Unadjusted P Critical Level Significant?**

Grand Mean vs. RpL7B 218056698,050 19,764 1,090E-070 0,000 Yes

Grand Mean vs. RpL40A 203878698,050 18,479 1,998E-063 0,000 Yes

Grand Mean vs. RpL23A 134953698,050 12,232 1,411E-031 0,000 Yes

Grand Mean vs. RpP2B 131609031,384 11,929 3,136E-030 0,000 Yes

Grand Mean vs. RpL19B 95024485,283 11,909 3,845E-030 0,000 Yes

Grand Mean vs. RpS7A 129196035,283 11,710 2,844E-029 0,000 Yes

Grand Mean vs. RpS29B 91269051,950 11,438 4,245E-028 0,000 Yes

Grand Mean vs. RpL13A 121474651,950 11,010 2,722E-026 0,000 Yes

Grand Mean vs. RpL20A 120307198,050 10,904 7,488E-026 0,000 Yes

Grand Mean vs. RpL16B 119461698,050 10,828 1,551E-025 0,000 Yes

Grand Mean vs. RpS10A 107166268,616 9,713 4,019E-021 0,000 Yes

Grand Mean vs. RpL15A 123757614,717 9,230 2,540E-019 0,000 Yes

Grand Mean vs. RpS9B 71506551,950 8,961 2,370E-018 0,000 Yes

Grand Mean vs. RpP0 95982531,384 8,700 1,985E-017 0,000 Yes

Grand Mean vs. RpS20 90949101,950 8,244 7,152E-016 0,000 Yes

Grand Mean vs. RpL30 86710518,616 7,859 1,295E-014 0,000 Yes

Grand Mean vs. RpS4A 94971810,283 7,083 3,172E-012 0,000 Yes

Grand Mean vs. RpL26A 76951864,717 6,975 6,581E-012 0,000 Yes

Grand Mean vs. RpS10B 76211301,950 6,908 1,029E-011 0,000 Yes

Grand Mean vs. RpL19A 74548531,384 6,757 2,773E-011 0,000 Yes

Grand Mean vs. RpL33A 74514635,283 6,754 2,829E-011 0,000 Yes

Grand Mean vs. RpS22B 73006531,384 6,617 6,838E-011 0,000 Yes

Grand Mean vs. RpS11A 72938635,283 6,611 7,112E-011 0,001 Yes

Grand Mean vs. RpL34B 70578135,283 6,397 0,000000000274 0,001 Yes

Grand Mean vs. RpL21B 70555698,050 6,395 0,000000000278 0,001 Yes

Grand Mean vs. RpS19B 69756968,616 6,323 0,000000000434 0,001 Yes

Grand Mean vs. RpS2 52264635,283 6,294 0,000000000517 0,001 Yes

Grand Mean vs. RpL41A 81319364,717 6,065 0,00000000206 0,001 Yes

Grand Mean vs. RpS1A 66528468,583 6,030 0,00000000254 0,001 Yes

Grand Mean vs. RpL26B 46163468,616 5,785 0,0000000105 0,001 Yes

Grand Mean vs. RpS21A 63098864,717 5,719 0,0000000153 0,001 Yes

Grand Mean vs. RpL34A 60793801,950 5,510 0,0000000488 0,001 Yes

Grand Mean vs. RpS19A 60709198,050 5,503 0,0000000509 0,001 Yes

Grand Mean vs. RpS15 60704031,384 5,502 0,0000000510 0,001 Yes

Grand Mean vs. RpL2A 60124635,283 5,450 0,0000000679 0,001 Yes

Grand Mean vs. RpS5 59067031,384 5,354 0,000000114 0,001 Yes

Grand Mean vs. RpL12B 58437031,384 5,297 0,000000154 0,001 Yes

Grand Mean vs. RpS12 57899301,950 5,248 0,000000199 0,001 Yes

Grand Mean vs. RpS21B 56586864,717 5,129 0,000000369 0,001 Yes

Grand Mean vs. RpS6B 40101301,950 5,026 0,000000624 0,001 Yes

Grand Mean vs. RpS4B 54548968,616 4,944 0,000000938 0,001 Yes

Grand Mean vs. RpL27B 52932364,717 4,798 0,00000193 0,001 Yes

Grand Mean vs. RpS30B 64021864,717 4,775 0,00000215 0,001 Yes

Grand Mean vs. RpS28A 52373635,283 4,747 0,00000246 0,001 Yes

Grand Mean vs. RpS1B 50575801,983 4,584 0,00000532 0,001 Yes

Grand Mean vs. RpL35B 48975364,717 4,439 0,0000103 0,001 Yes

Grand Mean vs. RpS18A 48658468,616 4,410 0,0000118 0,001 Yes

Grand Mean vs. RpS18B 46312968,616 4,198 0,0000301 0,001 Yes

Grand Mean vs. RpL33B 45765801,950 4,148 0,0000372 0,001 Yes

Grand Mean vs. RpL8B 44339698,050 4,019 0,0000642 0,001 Yes

Grand Mean vs. RpL9A 43508968,616 3,944 0,0000876 0,001 Yes

Grand Mean vs. RpL29 50023614,717 3,731 0,000205 0,001 Yes

Grand Mean vs. RpS16A 40568801,950 3,677 0,000252 0,001 Yes

Grand Mean vs. RpS27B 40325698,050 3,655 0,000274 0,001 Yes

Grand Mean vs. RpL37B 40256468,616 3,649 0,000281 0,001 Yes

Grand Mean vs. RpL32 39238198,050 3,557 0,000399 0,001 Yes

Grand Mean vs. RpS24B 39100698,050 3,544 0,000418 0,001 Yes

Grand Mean vs. RpP2A 38784698,050 3,515 0,000465 0,001 Yes

Grand Mean vs. RpL27A 37751635,283 3,422 0,000655 0,001 Yes

Grand Mean vs. RpL1B 36706531,384 3,327 0,000919 0,001 No

Grand Mean vs. RpL7A 36078301,950 3,270 0,00112 0,001 No

Grand Mean vs. RpS24A 35965135,283 3,260 0,00116 0,001 No

Grand Mean vs. RpL11B 34908698,050 3,164 0,00162 0,001 No

Grand Mean vs. RpS17A 34311301,950 3,110 0,00194 0,001 No

Grand Mean vs. RpL2B 33889531,384 3,072 0,00220 0,001 No

Grand Mean vs. RpS23A 33178468,616 3,007 0,00272 0,001 No

Grand Mean vs. RpS9A 23862385,283 2,991 0,00287 0,001 No

Grand Mean vs. RpL3 35729964,717 2,968 0,00309 0,001 No

Grand Mean vs. RpS11B 32023468,616 2,903 0,00381 0,001 No

Grand Mean vs. RpL24A 31602198,050 2,864 0,00429 0,001 No

Grand Mean vs. RpL24B 31585198,050 2,863 0,00431 0,001 No

Grand Mean vs. RpL8A 31360864,717 2,843 0,00459 0,001 No

Grand Mean vs. RpL43B 31263968,616 2,834 0,00472 0,001 No

Grand Mean vs. RpL40B 30772468,616 2,789 0,00541 0,001 No

Grand Mean vs. RpL31A 30467635,283 2,762 0,00589 0,001 No

Grand Mean vs. RpS14B 30073635,283 2,726 0,00656 0,001 No

Grand Mean vs. RpS3 29748135,283 2,696 0,00716 0,001 No

Grand Mean vs. RpL15B 27871698,050 2,526 0,0117 0,001 No

Grand Mean vs. RpS8A 27602698,050 2,502 0,0126 0,001 No

Grand Mean vs. RpL17A 26968864,717 2,444 0,0147 0,001 No

Grand Mean vs. RpL41B 26638198,050 2,414 0,0160 0,001 No

Grand Mean vs. RpS6A 18595551,950 2,330 0,0200 0,001 No

Grand Mean vs. RpL28 23805801,950 2,158 0,0313 0,001 No

Grand Mean vs. RpL6A 23377135,283 2,119 0,0344 0,001 No

Grand Mean vs. RpS7B 21108698,050 1,913 0,0561 0,001 No

Grand Mean vs. RpS0A 20802531,417 1,886 0,0597 0,001 No

Grand Mean vs. RpL12A 28902301,950 1,874 0,0613 0,001 No

Grand Mean vs. RpS25A 20482968,616 1,857 0,0638 0,001 No

Grand Mean vs. RpL36A 22289364,717 1,851 0,0645 0,001 No

Grand Mean vs. RpL14A 20028968,616 1,815 0,0698 0,001 No

Grand Mean vs. RpS0B 19679468,583 1,784 0,0749 0,002 No

Grand Mean vs. RpL1A 19511135,283 1,768 0,0774 0,002 No

Grand Mean vs. RpS17B 16878468,616 1,530 0,126 0,002 No

Grand Mean vs. RpL20B 16257031,384 1,474 0,141 0,002 No

Grand Mean vs. RpP1B 16006364,717 1,451 0,147 0,002 No

Grand Mean vs. RpS27A 15379301,950 1,394 0,164 0,002 No

Grand Mean vs. RpS16B 15360135,283 1,392 0,164 0,002 No

Grand Mean vs. RpS28B 14758364,717 1,338 0,181 0,002 No

Grand Mean vs. RpP1A 13698468,616 1,242 0,215 0,002 No

Grand Mean vs. RpL21A 13481801,950 1,222 0,222 0,002 No

Grand Mean vs. RpL22B 13243968,616 1,200 0,230 0,002 No

Grand Mean vs. RpS23B 17072635,283 1,107 0,269 0,002 No

Grand Mean vs. RpS22A 11396635,283 1,033 0,302 0,002 No

Grand Mean vs. RpL35A 9999031,384 0,906 0,365 0,002 No

Grand Mean vs. RpL18B 13625635,283 0,884 0,377 0,003 No

Grand Mean vs. RpS25B 9355801,950 0,848 0,397 0,003 No

Grand Mean vs. RpS30A 8741031,384 0,792 0,428 0,003 No

Grand Mean vs. RpL23B 8486968,616 0,769 0,442 0,003 No

Grand Mean vs. RpS31 8231698,050 0,746 0,456 0,003 No

Grand Mean vs. RpL37A 8017468,616 0,727 0,468 0,003 No

Grand Mean vs. RpL18A 7953301,950 0,721 0,471 0,004 No

Grand Mean vs. RpL10 7709468,616 0,699 0,485 0,004 No

Grand Mean vs. RpL42A 7220864,717 0,654 0,513 0,004 No

Grand Mean vs. RpL38 9345301,950 0,606 0,545 0,005 No

Grand Mean vs. RpL4A 4123635,283 0,517 0,605 0,005 No

Grand Mean vs. RpS29A 4369935,283 0,504 0,615 0,006 No

Grand Mean vs. RpL16A 5327364,717 0,483 0,629 0,006 No

Grand Mean vs. RpS13 4339164,717 0,360 0,719 0,007 No

Grand Mean vs. RpL6B 3362198,050 0,305 0,761 0,009 No

Grand Mean vs. RpL13B 2902801,950 0,263 0,793 0,010 No

Grand Mean vs. RpL25 2857364,717 0,259 0,796 0,013 No

Grand Mean vs. RpL22A 2055964,717 0,171 0,864 0,017 No

Grand Mean vs. RpS14A 1607031,384 0,146 0,884 0,025 No

Grand Mean vs. RpS26B 432364,717 0,0359 0,971 0,050 No
