# Supplementary material for: Specialized Yeast Ribosomes: A Customized Tool for Selective mRNA Translation
Source: PLoS One. 2013 Jul 8;8(7):e67609. doi: 10.1371/journal.pone.0067609 (PMC3704640; doi:10.1371/journal.pone.0067609)
Supplement: Table S9 — One way analysis of variance of REN[FFPTC] reporter readouts. (DOCX) [file pone.0067609.s010.docx]

**Supplementary Table S9:** One way analysis of variance of REN[FFPTC] reporter readouts.

**One Way Analysis of Variance**

**Data source:** REN[FFPTC] in ANOVAs.SNB

**Group Name N Missing Mean Std Dev SEM**

RpS0A 6 0 220049166,667 19366184,833 7906211,851

Row 2 5 0 159568600,000 9488370,055 4243328,088

RpS1A 6 0 138187500,000 4421748,059 1805171,086

RpS1B 6 0 129663000,000 4959299,991 2024625,743

RpS2 12 0 145658000,000 9512529,470 2746030,725

RpS3 6 0 142807666,667 11971092,108 4887177,888

RpS4A 6 0 111880833,333 3968827,908 1620267,209

RpS4B 6 0 141320666,667 8897189,976 3632262,598

RpS5 6 0 249584166,667 22002098,022 8982318,904

RpS6A 6 0 190580833,333 7347266,523 2999508,998

RpS6B 6 0 109736833,333 4785931,149 1953848,210

RpS7A 6 0 115102833,333 4946320,467 2019326,875

RpS7B 6 0 168537833,333 7747921,255 3163075,607

RpS8A 6 0 174825666,667 11927339,396 4869315,918

RpS9A 12 0 189468500,000 9212731,472 2659486,498

RpS9B 12 0 80876466,667 3044969,902 879007,096

RpS10A 6 0 85154283,333 6188432,884 2526417,146

RpS10B 6 0 160456666,667 3284836,353 1341028,826

RpS11A 6 0 88551833,333 4958947,431 2024481,811

RpS11B 6 0 123514333,333 4007083,112 1635884,830

RpS12 5 0 136432600,000 4428812,177 1980625,017

RpS13 6 0 134411500,000 13084275,704 5341633,188

RpS14A 6 0 200717666,667 6155141,222 2512825,881

RpS14B 6 0 240772333,333 12201368,475 4981187,821

RpS15 6 0 122090500,000 5912649,685 2413829,126

RpS16A 6 0 106461150,000 13905401,692 5676856,469

RpS16B 6 0 168922666,667 3040519,473 1241286,877

RpS17A 6 0 141661333,333 10759090,178 4392380,172

RpS17B 6 0 338237000,000 22723855,518 9276975,168

RpS18A 6 0 165838833,333 13688002,826 5588103,754

RpS18B 6 0 210290333,333 7351421,024 3001205,066

RpS19A 6 0 199207833,333 11999179,846 4898644,659

RpS19B 6 0 91001250,000 5179785,482 2114638,568

RpS20 6 0 96370483,333 5158747,442 2106049,824

RpS21A 6 0 227334000,000 13985657,110 5709620,606

RpS21B 6 0 191303666,667 5268050,101 2150672,448

RpS22A 6 0 134245166,667 7580381,505 3094677,790

RpS22B 6 0 152541000,000 12572691,534 5132779,825

RpS23A 6 0 141777500,000 7600397,694 3102849,365

RpS23B 6 0 160125166,667 14412018,588 5883681,951

RpS24A 6 0 143497000,000 8257963,357 3371299,423

RpS24B 6 0 201529833,333 10005372,425 4084676,188

RpS25A 6 0 140755666,667 4375864,288 1786439,115

RpS25B 6 0 137378333,333 9984592,223 4076192,706

RpS26B 6 0 157096166,667 3613945,014 1475386,874

RpS27A 4 0 232245750,000 21881160,852 10940580,426

RpS27B 6 0 401276166,667 17851705,817 7287928,382

RpS28A 6 0 210464500,000 21874417,668 8930193,618

RpS28B 6 0 192894000,000 7363203,135 3006015,092

RpS29A 12 0 166305916,667 11242850,103 3245531,267

RpS29B 12 0 93401950,000 5115204,152 1476632,247

RpS30A 6 0 155654666,667 7076079,951 2888797,543

RpS30B 6 0 265506000,000 16757155,606 6841080,129

RpS31 6 0 163815166,667 13231314,650 5401661,586

RpP0 6 0 168431000,000 4923981,763 2010207,137

RpP1A 4 0 169254000,000 9261001,458 4630500,729

RpP1B 6 0 224467166,667 5222781,689 2132191,696

RpP2A 6 0 250965500,000 11995659,477 4897207,474

RpP2B 5 0 193107800,000 16806358,847 7516032,167

RpL1A 6 0 283131666,667 15550932,135 6348641,459

RpL1B 6 0 161055500,000 5929615,966 2420755,581

RpL2A 6 0 151272000,000 5118543,543 2089636,651

RpL2B 6 0 413246666,667 27594000,802 11265203,654

RpL3 6 0 170544000,000 6467840,753 2640484,930

RpL4A 12 0 133949166,667 5305380,851 1531531,531

RpL6A 6 0 154638500,000 6341965,303 2589096,493

RpL6B 6 0 199739166,667 9721725,925 3968877,989

RpL7A 6 0 138105000,000 5916450,558 2415380,826

RpL7B 6 0 247100166,667 7508730,610 3065426,435

RpL8A 6 0 214713666,667 10815510,282 4415413,583

RpL8B 6 0 293409000,000 14317837,937 5845232,861

RpL9A 6 0 168519500,000 4559100,163 1861244,848

RpL10 6 0 398936333,333 37377184,092 15259171,508

RpL11B 6 0 298095666,667 38209033,815 15598772,735

RpL12A 4 0 162558500,000 1324792,688 662396,344

RpL12B 6 0 131969833,333 9612364,921 3924231,546

RpL13A 4 0 122872750,000 291789,165 145894,582

RpL13B 6 0 155105333,333 8998076,454 3673449,330

RpL14A 5 0 148888200,000 6805631,727 3043571,034

RpL15A 6 0 268456333,333 20085428,147 8199841,704

RpL15B 5 0 248264400,000 2056218,447 919568,845

RpL16A 6 0 178919166,667 7106251,429 2901114,997

RpL16B 6 0 191037666,667 5765694,257 2353834,824

RpL17A 3 0 164884333,333 6986288,738 4033535,684

RpL18A 6 0 203839333,333 3683623,687 1503833,073

RpL18B 6 0 159068000,000 4942218,530 2017652,266

RpL19A 6 0 210802500,000 7619255,049 3110547,848

RpL19B 12 0 124714250,000 4309551,783 1244060,441

RpL20A 6 0 318439166,667 14954067,813 6104972,620

RpL20B 6 0 197686333,333 7056302,960 2880723,620

RpL21A 6 0 131302333,333 7057302,138 2881131,533

RpL21B 6 0 390914000,000 11434368,107 4668061,232

RpL22A 6 0 155135833,333 4449662,658 1816567,173

RpL22B 6 0 184688000,000 3672134,747 1499142,733

RpL23A 6 0 215151666,667 14661627,572 5985584,392

RpL23B 5 0 144864200,000 1584330,679 708534,219

RpL24A 5 0 262318000,000 6173763,196 2760990,837

RpL24B 6 0 176249500,000 10525132,336 4296867,283

RpL25 6 0 163112666,667 10737955,833 4383752,112

RpL26A 6 0 258191333,333 8906587,195 3636098,996

RpL26B 12 0 146746000,000 9577868,191 2764892,389

RpL27A 6 0 189184166,667 14054875,189 5737878,769

RpL27B 5 0 176275000,000 10625188,022 4751728,538

RpL28 6 0 175513000,000 6828272,256 2787630,475

RpL29 4 0 121826000,000 11716569,578 5858284,789

RpL30 6 0 128253500,000 5468912,826 2232674,312

RpL31A 6 0 126347500,000 7502868,858 3063033,385

RpL32 6 0 149926166,667 5754565,819 2349291,658

RpL33A 6 0 131668833,333 6750699,295 2755961,447

RpL33B 3 0 138658000,000 249240,847 143899,270

RpL34A 6 0 106242833,333 2806514,808 1145754,873

RpL34B 6 0 132543666,667 4085670,798 1667968,119

RpL35A 6 0 197731833,333 10611002,449 4331923,610

RpL35B 6 0 153794500,000 8470019,663 3457871,048

RpL36A 6 0 177281000,000 8956889,907 3656634,992

RpL37A 6 0 144103333,333 6122140,693 2499353,472

RpL37B 6 0 146369333,333 6700225,031 2735355,415

RpL38 6 0 138789833,333 4867803,998 1987272,660

RpL40A 4 0 185813750,000 8272581,716 4136290,858

RpL40B 6 0 168447833,333 9217187,042 3762900,853

RpL41A 6 0 208756333,333 7628278,224 3114231,544

RpL41B 6 0 206909333,333 19039298,723 7772761,155

RpL42A 6 0 213286166,667 13430493,385 5482975,965

RpL43B 6 0 183715666,667 14081138,515 5748600,727

Grand Mean 124 0 179817640,860 63715721,695 5721840,719

**Source of Variation DF SS MS F P**

Between Groups 124 3,100E+018 2,500E+016 33,087 <0,001

Residual 765 5,781E+017 7,557E+014

Total 889 3,678E+018

The differences in the mean values among the treatment groups are greater than would be expected by chance; there is a statistically significant difference (P = <0,001).

Power of performed test with alpha = 0,050: 1,000

Multiple Comparisons versus Control Group (Holm-Sidak method):

Overall significance level = 0,05

Comparisons for factor:

**Comparison Diff of Means t Unadjusted P Critical Level Significant?**

Grand Mean vs. RpL2B 233429025,807 20,314 1,048E-073 0,000 Yes

Grand Mean vs. RpS27B 221458525,807 19,273 9,054E-068 0,000 Yes

Grand Mean vs. RpL10 219118692,473 19,069 1,274E-066 0,000 Yes

Grand Mean vs. RpL21B 211096359,140 18,371 1,020E-062 0,000 Yes

Grand Mean vs. RpS17B 158419359,140 13,787 8,821E-039 0,000 Yes

Grand Mean vs. RpL20A 138621525,807 12,064 8,423E-031 0,000 Yes

Grand Mean vs. RpS9B 98941174,193 11,905 4,206E-030 0,000 Yes

Grand Mean vs. RpS29B 86415690,860 10,398 8,892E-024 0,000 Yes

Grand Mean vs. RpL11B 118278025,807 10,293 2,325E-023 0,000 Yes

Grand Mean vs. RpL8B 113591359,140 9,885 9,095E-022 0,000 Yes

Grand Mean vs. RpL1A 103314025,807 8,991 1,896E-018 0,000 Yes

Grand Mean vs. RpS10A 94663357,527 8,238 7,561E-016 0,000 Yes

Grand Mean vs. RpS11A 91265807,527 7,942 7,075E-015 0,000 Yes

Grand Mean vs. RpS19B 88816390,860 7,729 3,403E-014 0,000 Yes

Grand Mean vs. RpL15A 88638692,473 7,714 3,808E-014 0,000 Yes

Grand Mean vs. RpS30B 85688359,140 7,457 2,402E-013 0,000 Yes

Grand Mean vs. RpS20 83447157,527 7,262 9,399E-013 0,000 Yes

Grand Mean vs. RpL26A 78373692,473 6,821 1,843E-011 0,000 Yes

Grand Mean vs. RpL19B 55103390,860 6,630 6,322E-011 0,000 Yes

Grand Mean vs. RpL24A 82500359,140 6,579 8,756E-011 0,000 Yes

Grand Mean vs. RpL34A 73574807,527 6,403 0,000000000266 0,000 Yes

Grand Mean vs. RpS16A 73356490,860 6,384 0,000000000299 0,000 Yes

Grand Mean vs. RpP2A 71147859,140 6,192 0,000000000971 0,001 Yes

Grand Mean vs. RpS6B 70080807,527 6,099 0,00000000170 0,001 Yes

Grand Mean vs. RpS5 69766525,807 6,071 0,00000000199 0,001 Yes

Grand Mean vs. RpS4A 67936807,527 5,912 0,00000000508 0,001 Yes

Grand Mean vs. RpL7B 67282525,807 5,855 0,00000000707 0,001 Yes

Grand Mean vs. RpS7A 64714807,527 5,632 0,0000000250 0,001 Yes

Grand Mean vs. RpL4A 45868474,193 5,519 0,0000000466 0,001 Yes

Grand Mean vs. RpL15B 68446759,140 5,459 0,0000000649 0,001 Yes

Grand Mean vs. RpS14B 60954692,473 5,305 0,000000148 0,001 Yes

Grand Mean vs. RpS15 57727140,860 5,024 0,000000631 0,001 Yes

Grand Mean vs. RpS11B 56303307,527 4,900 0,00000117 0,001 Yes

Grand Mean vs. RpL31A 53470140,860 4,653 0,00000385 0,001 Yes

Grand Mean vs. RpL30 51564140,860 4,487 0,00000832 0,001 Yes

Grand Mean vs. RpS1B 50154640,860 4,365 0,0000145 0,001 Yes

Grand Mean vs. RpL21A 48515307,527 4,222 0,0000271 0,001 Yes

Grand Mean vs. RpL33A 48148807,527 4,190 0,0000311 0,001 Yes

Grand Mean vs. RpL12B 47847807,527 4,164 0,0000348 0,001 Yes

Grand Mean vs. RpL29 57991640,860 4,153 0,0000366 0,001 Yes

Grand Mean vs. RpS21A 47516359,140 4,135 0,0000394 0,001 Yes

Grand Mean vs. RpL34B 47273974,193 4,114 0,0000431 0,001 Yes

Grand Mean vs. RpS2 34159640,860 4,110 0,0000438 0,001 Yes

Grand Mean vs. RpL13A 56944890,860 4,078 0,0000502 0,001 Yes

Grand Mean vs. RpL26B 33071640,860 3,979 0,0000757 0,001 Yes

Grand Mean vs. RpS22A 45572474,193 3,966 0,0000800 0,001 Yes

Grand Mean vs. RpS13 45406140,860 3,951 0,0000849 0,001 Yes

Grand Mean vs. RpP1B 44649525,807 3,886 0,000111 0,001 Yes

Grand Mean vs. RpS27A 52428109,140 3,754 0,000187 0,001 Yes

Grand Mean vs. RpS25B 42439307,527 3,693 0,000237 0,001 Yes

Grand Mean vs. RpL7A 41712640,860 3,630 0,000302 0,001 Yes

Grand Mean vs. RpS1A 41630140,860 3,623 0,000311 0,001 Yes

Grand Mean vs. RpL38 41027807,527 3,570 0,000379 0,001 Yes

Grand Mean vs. RpS0A 40231525,807 3,501 0,000490 0,001 Yes

Grand Mean vs. RpS12 43385040,860 3,460 0,000570 0,001 Yes

Grand Mean vs. RpS25A 39061974,193 3,399 0,000710 0,001 Yes

Grand Mean vs. RpS4B 38496974,193 3,350 0,000847 0,001 No

Grand Mean vs. RpS17A 38156307,527 3,321 0,000941 0,001 No

Grand Mean vs. RpS23A 38040140,860 3,310 0,000975 0,001 No

Grand Mean vs. RpS3 37009974,193 3,221 0,00133 0,001 No

Grand Mean vs. RpS24A 36320640,860 3,161 0,00164 0,001 No

Grand Mean vs. RpL37A 35714307,527 3,108 0,00195 0,001 No

Grand Mean vs. RpL23A 35334025,807 3,075 0,00218 0,001 No

Grand Mean vs. RpL8A 34896025,807 3,037 0,00247 0,001 No

Grand Mean vs. RpL42A 33468525,807 2,913 0,00369 0,001 No

Grand Mean vs. RpL37B 33448307,527 2,911 0,00371 0,001 No

Grand Mean vs. RpL23B 34953440,860 2,788 0,00544 0,001 No

Grand Mean vs. RpL19A 30984859,140 2,696 0,00716 0,001 No

Grand Mean vs. RpS28A 30646859,140 2,667 0,00781 0,001 No

Grand Mean vs. RpS18B 30472692,473 2,652 0,00817 0,001 No

Grand Mean vs. RpL32 29891474,193 2,601 0,00947 0,001 No

Grand Mean vs. RpL33B 41159640,860 2,563 0,0106 0,001 No

Grand Mean vs. RpL41A 28938692,473 2,518 0,0120 0,001 No

Grand Mean vs. RpL2A 28545640,860 2,484 0,0132 0,001 No

Grand Mean vs. RpL14A 30929440,860 2,467 0,0139 0,001 No

Grand Mean vs. RpS22B 27276640,860 2,374 0,0179 0,001 No

Grand Mean vs. RpL41B 27091692,473 2,358 0,0186 0,001 No

Grand Mean vs. RpL35B 26023140,860 2,265 0,0238 0,001 No

Grand Mean vs. RpL6A 25179140,860 2,191 0,0287 0,001 No

Grand Mean vs. RpL13B 24712307,527 2,151 0,0318 0,001 No

Grand Mean vs. RpL22A 24681807,527 2,148 0,0320 0,001 No

Grand Mean vs. RpS30A 24162974,193 2,103 0,0358 0,001 No

Grand Mean vs. RpL18A 24021692,473 2,091 0,0369 0,001 No

Grand Mean vs. RpS26B 22721474,193 1,977 0,0484 0,001 No

Grand Mean vs. RpS24B 21712192,473 1,890 0,0592 0,001 No

Grand Mean vs. RpS14A 20900025,807 1,819 0,0693 0,001 No

Grand Mean vs. RpL18B 20749640,860 1,806 0,0714 0,001 No

Grand Mean vs. RpL6B 19921525,807 1,734 0,0834 0,001 No

Grand Mean vs. RpS23B 19692474,193 1,714 0,0870 0,001 No

Grand Mean vs. RpS19A 19390192,473 1,687 0,0919 0,001 No

Grand Mean vs. RpS10B 19360974,193 1,685 0,0924 0,002 No

Grand Mean vs. RpL1B 18762140,860 1,633 0,103 0,002 No

Grand Mean vs. RpS29A 13511724,193 1,626 0,104 0,002 No

Grand Mean vs. Row 2 20249040,860 1,615 0,107 0,002 No

Grand Mean vs. RpL35A 17914192,473 1,559 0,119 0,002 No

Grand Mean vs. RpL20B 17868692,473 1,555 0,120 0,002 No

Grand Mean vs. RpL25 16704974,193 1,454 0,146 0,002 No

Grand Mean vs. RpS31 16002474,193 1,393 0,164 0,002 No

Grand Mean vs. RpL12A 17259140,860 1,236 0,217 0,002 No

Grand Mean vs. RpS18A 13978807,527 1,217 0,224 0,002 No

Grand Mean vs. RpS9A 9650859,140 1,161 0,246 0,002 No

Grand Mean vs. RpS28B 13076359,140 1,138 0,255 0,002 No

Grand Mean vs. RpP2B 13290159,140 1,060 0,290 0,002 No

Grand Mean vs. RpS21B 11486025,807 1,000 0,318 0,002 No

Grand Mean vs. RpP0 11386640,860 0,991 0,322 0,003 No

Grand Mean vs. RpL40B 11369807,527 0,989 0,323 0,003 No

Grand Mean vs. RpL9A 11298140,860 0,983 0,326 0,003 No

Grand Mean vs. RpS7B 11279807,527 0,982 0,327 0,003 No

Grand Mean vs. RpL16B 11220025,807 0,976 0,329 0,003 No

Grand Mean vs. RpS16B 10894974,193 0,948 0,343 0,003 No

Grand Mean vs. RpS6A 10763192,473 0,937 0,349 0,004 No

Grand Mean vs. RpL17A 14933307,527 0,930 0,353 0,004 No

Grand Mean vs. RpL27A 9366525,807 0,815 0,415 0,004 No

Grand Mean vs. RpL3 9273640,860 0,807 0,420 0,005 No

Grand Mean vs. RpP1A 10563640,860 0,756 0,450 0,005 No

Grand Mean vs. RpS8A 4991974,193 0,434 0,664 0,006 No

Grand Mean vs. RpL40A 5996109,140 0,429 0,668 0,006 No

Grand Mean vs. RpL22B 4870359,140 0,424 0,672 0,007 No

Grand Mean vs. RpL28 4304640,860 0,375 0,708 0,009 No

Grand Mean vs. RpL43B 3898025,807 0,339 0,735 0,010 No

Grand Mean vs. RpL24B 3568140,860 0,311 0,756 0,013 No

Grand Mean vs. RpL27B 3542640,860 0,283 0,778 0,017 No

Grand Mean vs. RpL36A 2536640,860 0,221 0,825 0,025 No

Grand Mean vs. RpL16A 898474,193 0,0782 0,938 0,050 No
