# Supplementary material for: Specialized Yeast Ribosomes: A Customized Tool for Selective mRNA Translation
Source: PLoS One. 2013 Jul 8;8(7):e67609. doi: 10.1371/journal.pone.0067609 (PMC3704640; doi:10.1371/journal.pone.0067609)
Supplement: Table S10 — One way analysis of variance of REN[LA3FF] reporter readouts. (DOCX) [file pone.0067609.s011.docx]

**Supplementary Table S10:** One way analysis of variance of REN[LA3FF] reporter readouts.

**One Way Analysis of Variance**

**Data source:** REN[LA3FF] in ANOVAs.SNB

**Group Name N Missing Mean Std Dev SEM**

RpS0A 6 0 210250166,700 11892920,370 4855264,410

RpS0B 6 0 168025166,700 6796624,733 2774710,428

RpS1A 6 0 231125166,700 14552665,300 5941100,730

RpS1B 6 0 220380500,000 18137663,452 7404670,097

RpS2 12 0 140720333,333 8640395,260 2494267,265

RpS3 6 0 169031666,667 6364968,363 2598487,453

RpS4A 5 0 120355400,000 6234153,856 2787998,361

RpS4B 6 0 146099500,000 14804689,646 6043989,239

RpS5 5 0 431753800,000 18250063,499 8161676,515

RpS6A 12 0 199817250,000 15171666,356 4379682,827

RpS6B 12 0 151743000,000 14629827,707 4223267,482

RpS7A 6 0 142938000,000 5487486,601 2240257,024

RpS7B 6 0 232382666,667 19732960,058 8055947,209

RpS8A 6 0 223619333,333 17820635,552 7275243,999

RpS9A 12 0 204753500,000 13632451,347 3935349,727

RpS9B 12 0 124530416,667 6148449,686 1774904,541

RpS10A 6 0 75073766,667 4067600,243 1660590,845

RpS10B 5 0 131986000,000 8012569,656 3583330,085

RpS11A 6 0 130030666,667 5441179,603 2221352,271

RpS11B 6 0 159408166,667 5140094,101 2098434,630

RpS12 6 0 159993166,667 4948037,527 2020027,862

RpS13 6 0 154336833,333 8093658,837 3304222,384

RpS14A 6 0 267802000,000 26564889,512 10845070,730

RpS14B 6 0 213361000,000 11251558,843 4593429,663

RpS15 6 0 168649333,333 4510632,579 1841458,039

RpS16A 6 0 190715166,667 5468821,131 2232636,878

RpS16B 6 0 169192000,000 17943678,842 7325476,212

RpS17A 6 0 188604500,000 7954861,023 3247558,414

RpS17B 6 0 201863500,000 11258458,593 4596246,474

RpS18A 6 0 200440166,667 15927710,977 6502460,777

RpS18B 6 0 156966000,000 10030740,431 4095032,633

RpS19A 6 0 178300500,000 17328573,706 7074360,592

RpS19B 6 0 192798500,000 17092510,638 6977988,248

RpS20 6 0 142897500,000 10181416,753 4156545,984

RpS21A 6 0 235038833,333 14625786,986 5970952,534

RpS21B 6 0 201781333,333 10153696,463 4145229,223

RpS22A 6 0 165334333,333 12229892,946 4992832,888

RpS22B 6 0 202239333,333 10193422,284 4161447,221

RpS23A 5 0 154164000,000 6883792,886 3078525,767

RpS23B 6 0 217155666,667 17383492,246 7096780,992

RpS24A 6 0 278426000,000 11256821,328 4595578,063

RpS24B 6 0 309767000,000 23242517,600 9488718,076

RpS25A 6 0 289544833,333 18651910,716 7614610,664

RpS25B 6 0 199784166,667 15635277,911 6383075,478

RpS26B 6 0 214039666,667 18993719,422 7754153,484

RpS27A 6 0 297367500,000 22769128,238 9295457,679

RpS27B 6 0 575217500,000 48551132,904 19820917,008

RpS28A 6 0 262539333,333 23836725,024 9731302,241

RpS28B 6 0 233050833,333 15268794,326 6233459,181

RpS29A 12 0 146841000,000 13913825,838 4016575,547

RpS29B 12 0 103716950,000 7187969,923 2074988,185

RpS30A 6 0 191258333,333 8235503,789 3362130,343

RpS30B 6 0 327257500,000 20346435,980 8306397,706

RpS31 4 0 257237250,000 14051235,115 7025617,558

RpP0 6 0 283105333,333 20365460,816 8314164,563

RpP1A 5 0 294747400,000 8168053,673 3652864,651

RpP1B 6 0 313517166,667 19257919,850 7862012,857

RpP2A 5 0 303660600,000 20333838,307 9093568,940

RpP2B 6 0 243557166,667 9686254,207 3954396,721

RpL1A 6 0 95089000,000 3051634,631 1245824,621

RpL1B 6 0 218315666,667 14594737,677 5958276,706

RpL2A 5 0 241158000,000 23994049,200 10730465,013

RpL2B 5 0 344672600,000 35374237,678 15819840,020

RpL3 6 0 195538833,333 14846377,732 6061008,329

RpL4A 12 0 137288083,333 5391820,277 1556484,444

RpL6A 6 0 147887333,333 5069546,357 2069633,634

RpL6B 5 0 251105400,000 28299870,853 12656086,996

RpL7A 6 0 238917166,667 9458307,849 3861338,010

RpL7B 6 0 271411500,000 12334520,562 5035546,933

RpL8A 6 0 199414166,667 11667566,574 4763264,108

RpL8B 5 0 349604000,000 17037977,389 7619615,128

RpL9A 6 0 243807500,000 15331841,765 6259198,190

RpL10 6 0 354834833,333 19347879,619 7898738,779

RpS4A4 6 0 253591500,000 12148510,324 4959608,571

RpL12A 5 0 154143000,000 10292902,239 4603125,818

RpL12B 6 0 335492000,000 16757953,419 6841405,835

RpL13A 6 0 121850183,333 21100158,917 8614103,806

RpL13B 3 0 204369000,000 6204658,572 3582261,297

RpL14A 6 0 236926000,000 12837453,548 5240868,465

RpL15A 6 0 249845833,333 19574838,108 7991394,194

RpL15B 6 0 204071000,000 13076386,275 5338412,342

RpL16A 6 0 206660666,667 9154263,939 3737212,604

RpL16B 6 0 343768333,333 28705788,682 11719089,156

RpL17A 5 0 429286200,000 51290810,514 22937947,786

RpL18A 6 0 221399833,333 24413496,451 9966768,190

RpL18B 6 0 184309666,667 11594802,882 4733558,455

RpL19A 6 0 286443333,333 23318012,134 9519538,591

RpL19B 12 0 109419475,000 7227893,699 2086513,186

RpL20A 6 0 286703833,333 24447035,464 9980460,435

RpL20B 6 0 276174500,000 14998326,183 6123041,024

RpL21A 6 0 184806000,000 8066033,548 3292944,407

RpL21B 6 0 267956333,333 11370187,240 4641859,503

RpL22A 6 0 476421333,333 68814448,008 28093380,758

RpL22B 6 0 240978000,000 16409937,599 6699328,971

RpL23A 6 0 329809000,000 16245761,872 6632304,512

RpL23B 6 0 239209333,333 9958589,211 4065577,021

RpL24A 6 0 229539333,333 11843738,239 4835185,889

RpL24B 6 0 242794333,333 11146142,807 4550393,746

RpL25 6 0 273351500,000 15154281,544 6186709,534

RpL26A 6 0 281781500,000 10641178,765 4344243,039

RpL26B 12 0 192222916,667 9982843,970 2881798,827

RpL27A 6 0 375791666,667 19612460,515 8006753,477

RpL27B 6 0 193460333,333 8009674,991 3269936,122

RpL28 6 0 243334000,000 14683123,700 5994360,149

RpL29 6 0 275006666,667 17417375,550 7110613,793

RpL30 6 0 130062500,000 6262394,869 2556611,999

RpL31A 6 0 221793666,667 9145001,119 3733431,073

RpL32 6 0 360826500,000 19184287,933 7831952,752

RpL33A 5 0 150748600,000 5185869,146 2319191,187

RpL33B 6 0 215545000,000 12671237,082 5173010,877

RpL34A 6 0 141491000,000 3345974,836 1365988,507

RpL34B 6 0 170181000,000 7507907,938 3065090,581

RpL35A 6 0 254171500,000 22756344,388 9290238,694

RpL35B 6 0 196581166,667 7604708,842 3104609,384

RpL36A 6 0 211299666,667 15468359,741 6314931,421

RpL37A 6 0 186380500,000 11324581,136 4623240,889

RpL37B 6 0 246742166,667 16789521,297 6854293,367

RpL38 6 0 204130666,667 7650378,073 3123253,770

RpL40A 6 0 288340500,000 10306913,714 4207779,904

RpL40B 6 0 228677166,667 10102589,320 4124364,819

RpL41A 6 0 322098333,333 15314680,652 6252192,195

RpL41B 5 0 242628600,000 7265017,983 3249014,814

RpL42A 6 0 213641333,333 21532847,287 8790748,094

RpL43B 6 0 197438666,667 12956909,333 5289636,085

Grand Mean 124 0 226798652,353 78329612,654 7034206,869

**Source of Variation DF SS MS F P**

Between Groups 124 4,753E+018 3,833E+016 32,117 <0,001

Residual 784 9,356E+017 1,193E+015

Total 908 5,688E+018

The differences in the mean values among the treatment groups are greater than would be expected by chance; there is a statistically significant difference (P = <0,001).

Power of performed test with alpha = 0,050: 1,000

Multiple Comparisons versus Control Group (Holm-Sidak method):

Overall significance level = 0,05

Comparisons for factor:

**Comparison Diff of Means t Unadjusted P Critical Level Significant?**

Grand Mean vs. RpS27B 348418847,647 24,128 1,244E-096 0,000 Yes

Grand Mean vs. RpL22A 249622680,980 17,286 5,744E-057 0,000 Yes

Grand Mean vs. RpS5 204955147,647 13,007 3,668E-035 0,000 Yes

Grand Mean vs. RpL17A 202487547,647 12,850 1,966E-034 0,000 Yes

Grand Mean vs. RpS29B 123081702,353 11,785 1,250E-029 0,000 Yes

Grand Mean vs. RpL19B 117379177,353 11,239 2,812E-027 0,000 Yes

Grand Mean vs. RpS10A 151724885,686 10,507 3,002E-024 0,000 Yes

Grand Mean vs. RpL27A 148993014,314 10,318 1,721E-023 0,000 Yes

Grand Mean vs. RpS9B 102268235,686 9,792 1,943E-021 0,000 Yes

Grand Mean vs. RpL32 134027847,647 9,281 1,600E-019 0,000 Yes

Grand Mean vs. RpL1A 131709652,353 9,121 6,162E-019 0,000 Yes

Grand Mean vs. RpL10 128036180,980 8,866 5,026E-018 0,000 Yes

Grand Mean vs. RpL4A 89510569,020 8,571 5,441E-017 0,000 Yes

Grand Mean vs. RpS2 86078319,020 8,242 7,101E-016 0,000 Yes

Grand Mean vs. RpL16B 116969680,980 8,100 2,098E-015 0,000 Yes

Grand Mean vs. RpL8B 122805347,647 7,793 2,074E-014 0,000 Yes

Grand Mean vs. RpS29A 79957652,353 7,656 5,651E-014 0,000 Yes

Grand Mean vs. RpL12B 108693347,647 7,527 1,428E-013 0,000 Yes

Grand Mean vs. RpL2B 117873947,647 7,480 1,990E-013 0,000 Yes

Grand Mean vs. RpL13A 104948469,020 7,268 8,853E-013 0,000 Yes

Grand Mean vs. RpS6B 75055652,353 7,187 1,550E-012 0,000 Yes

Grand Mean vs. RpL23A 103010347,647 7,133 2,229E-012 0,000 Yes

Grand Mean vs. RpS30B 100458847,647 6,957 7,351E-012 0,001 Yes

Grand Mean vs. RpS4A 106443252,353 6,755 2,785E-011 0,001 Yes

Grand Mean vs. RpS11A 96767985,686 6,701 3,949E-011 0,001 Yes

Grand Mean vs. RpL30 96736152,353 6,699 4,006E-011 0,001 Yes

Grand Mean vs. RpL41A 95299680,980 6,599 7,595E-011 0,001 Yes

Grand Mean vs. RpS10B 94812652,353 6,017 0,00000000273 0,001 Yes

Grand Mean vs. RpP1B 86718514,314 6,005 0,00000000292 0,001 Yes

Grand Mean vs. RpL34A 85307652,353 5,908 0,00000000517 0,001 Yes

Grand Mean vs. RpS20 83901152,353 5,810 0,00000000907 0,001 Yes

Grand Mean vs. RpS7A 83860652,353 5,807 0,00000000922 0,001 Yes

Grand Mean vs. RpS24B 82968347,647 5,746 0,0000000131 0,001 Yes

Grand Mean vs. RpS4B 80699152,353 5,588 0,0000000316 0,001 Yes

Grand Mean vs. RpL6A 78911319,020 5,465 0,0000000624 0,001 Yes

Grand Mean vs. RpS13 72461819,020 5,018 0,000000647 0,001 Yes

Grand Mean vs. RpS27A 70568847,647 4,887 0,00000124 0,001 Yes

Grand Mean vs. RpP2A 76861947,647 4,878 0,00000130 0,001 Yes

Grand Mean vs. RpS18B 69832652,353 4,836 0,00000160 0,001 Yes

Grand Mean vs. RpL33A 76050052,353 4,826 0,00000167 0,001 Yes

Grand Mean vs. RpS11B 67390485,686 4,667 0,00000360 0,001 Yes

Grand Mean vs. RpS12 66805485,686 4,626 0,00000435 0,001 Yes

Grand Mean vs. RpL12A 72655652,353 4,611 0,00000468 0,001 Yes

Grand Mean vs. RpS23A 72634652,353 4,609 0,00000471 0,001 Yes

Grand Mean vs. RpS25A 62746180,980 4,345 0,0000157 0,001 Yes

Grand Mean vs. RpP1A 67948747,647 4,312 0,0000182 0,001 Yes

Grand Mean vs. RpL40A 61541847,647 4,262 0,0000228 0,001 Yes

Grand Mean vs. RpS22A 61464319,020 4,256 0,0000233 0,001 Yes

Grand Mean vs. RpL20A 59905180,980 4,148 0,0000371 0,001 Yes

Grand Mean vs. RpL19A 59644680,980 4,130 0,0000401 0,001 Yes

Grand Mean vs. RpS0B 58773485,653 4,070 0,0000518 0,001 Yes

Grand Mean vs. RpS15 58149319,020 4,027 0,0000620 0,001 Yes

Grand Mean vs. RpS3 57766985,686 4,000 0,0000692 0,001 Yes

Grand Mean vs. RpS16B 57606652,353 3,989 0,0000725 0,001 Yes

Grand Mean vs. RpL34B 56617652,353 3,921 0,0000960 0,001 Yes

Grand Mean vs. RpP0 56306680,980 3,899 0,000105 0,001 Yes

Grand Mean vs. RpL26A 54982847,647 3,808 0,000151 0,001 Yes

Grand Mean vs. RpS24A 51627347,647 3,575 0,000371 0,001 Yes

Grand Mean vs. RpL20B 49375847,647 3,419 0,000660 0,001 Yes

Grand Mean vs. RpS19A 48498152,353 3,358 0,000822 0,001 No

Grand Mean vs. RpL29 48208014,314 3,338 0,000882 0,001 No

Grand Mean vs. RpL26B 34575735,686 3,311 0,000974 0,001 No

Grand Mean vs. RpL25 46552847,647 3,224 0,00132 0,001 No

Grand Mean vs. RpL7B 44612847,647 3,089 0,00208 0,001 No

Grand Mean vs. RpL18B 42488985,686 2,942 0,00335 0,001 No

Grand Mean vs. RpL21A 41992652,353 2,908 0,00374 0,001 No

Grand Mean vs. RpL21B 41157680,980 2,850 0,00448 0,001 No

Grand Mean vs. RpS14A 41003347,647 2,839 0,00464 0,001 No

Grand Mean vs. RpL37A 40418152,353 2,799 0,00525 0,001 No

Grand Mean vs. RpS17A 38194152,353 2,645 0,00833 0,001 No

Grand Mean vs. RpS6A 26981402,353 2,583 0,00996 0,001 No

Grand Mean vs. RpS16A 36083485,686 2,499 0,0127 0,001 No

Grand Mean vs. RpS28A 35740680,980 2,475 0,0135 0,001 No

Grand Mean vs. RpS30A 35540319,020 2,461 0,0141 0,001 No

Grand Mean vs. RpS19B 34000152,353 2,355 0,0188 0,001 No

Grand Mean vs. RpL27B 33338319,020 2,309 0,0212 0,001 No

Grand Mean vs. RpL3 31259819,020 2,165 0,0307 0,001 No

Grand Mean vs. RpS9A 22045152,353 2,111 0,0351 0,001 No

Grand Mean vs. RpL35B 30217485,686 2,093 0,0367 0,001 No

Grand Mean vs. RpL43B 29359985,686 2,033 0,0424 0,001 No

Grand Mean vs. RpL8A 27384485,686 1,896 0,0583 0,001 No

Grand Mean vs. RpL35A 27372847,647 1,896 0,0584 0,001 No

Grand Mean vs. RpS25B 27014485,686 1,871 0,0618 0,001 No

Grand Mean vs. RpS4A4 26792847,647 1,855 0,0639 0,001 No

Grand Mean vs. RpS18A 26358485,686 1,825 0,0683 0,001 No

Grand Mean vs. RpS31 30438597,647 1,734 0,0832 0,001 No

Grand Mean vs. RpS21B 25017319,020 1,732 0,0836 0,001 No

Grand Mean vs. RpS17B 24935152,353 1,727 0,0846 0,001 No

Grand Mean vs. RpS22B 24559319,020 1,701 0,0894 0,001 No

Grand Mean vs. RpL15A 23047180,980 1,596 0,111 0,001 No

Grand Mean vs. RpL15B 22727652,353 1,574 0,116 0,002 No

Grand Mean vs. RpL38 22667985,686 1,570 0,117 0,002 No

Grand Mean vs. RpL6B 24306747,647 1,543 0,123 0,002 No

Grand Mean vs. RpL16A 20137985,686 1,395 0,164 0,002 No

Grand Mean vs. RpL37B 19943514,314 1,381 0,168 0,002 No

Grand Mean vs. RpL9A 17008847,647 1,178 0,239 0,002 No

Grand Mean vs. RpP2B 16758514,314 1,161 0,246 0,002 No

Grand Mean vs. RpS0A 16548485,653 1,146 0,252 0,002 No

Grand Mean vs. RpL28 16535347,647 1,145 0,253 0,002 No

Grand Mean vs. RpL13B 22429652,353 1,111 0,267 0,002 No

Grand Mean vs. RpL24B 15995680,980 1,108 0,268 0,002 No

Grand Mean vs. RpL36A 15498985,686 1,073 0,283 0,002 No

Grand Mean vs. RpL41B 15829947,647 1,005 0,315 0,002 No

Grand Mean vs. RpL22B 14179347,647 0,982 0,326 0,002 No

Grand Mean vs. RpS14B 13437652,353 0,931 0,352 0,003 No

Grand Mean vs. RpL2A 14359347,647 0,911 0,362 0,003 No

Grand Mean vs. RpL42A 13157319,020 0,911 0,363 0,003 No

Grand Mean vs. RpS26B 12758985,686 0,884 0,377 0,003 No

Grand Mean vs. RpL23B 12410680,980 0,859 0,390 0,003 No

Grand Mean vs. RpL7A 12118514,314 0,839 0,402 0,003 No

Grand Mean vs. RpL33B 11253652,353 0,779 0,436 0,004 No

Grand Mean vs. RpL14A 10127347,647 0,701 0,483 0,004 No

Grand Mean vs. RpS23B 9642985,686 0,668 0,504 0,004 No

Grand Mean vs. RpL1B 8482985,686 0,587 0,557 0,005 No

Grand Mean vs. RpS21A 8240180,980 0,571 0,568 0,005 No

Grand Mean vs. RpS1B 6418152,353 0,444 0,657 0,006 No

Grand Mean vs. RpS28B 6252180,980 0,433 0,665 0,006 No

Grand Mean vs. RpS7B 5584014,314 0,387 0,699 0,007 No

Grand Mean vs. RpL18A 5398819,020 0,374 0,709 0,009 No

Grand Mean vs. RpL31A 5004985,686 0,347 0,729 0,010 No

Grand Mean vs. RpS1A 4326514,347 0,300 0,765 0,013 No

Grand Mean vs. RpS8A 3179319,020 0,220 0,826 0,017 No

Grand Mean vs. RpL24A 2740680,980 0,190 0,850 0,025 No

Grand Mean vs. RpL40B 1878514,314 0,130 0,897 0,050 No
