# Supplementary material for: Specialized Yeast Ribosomes: A Customized Tool for Selective mRNA Translation
Source: PLoS One. 2013 Jul 8;8(7):e67609. doi: 10.1371/journal.pone.0067609 (PMC3704640; doi:10.1371/journal.pone.0067609)
Supplement: Table S11 — One way analysis of variance of REN[LA3PTCFF] reporter readouts. (DOCX) [file pone.0067609.s012.docx]

**Supplementary Table S11:** One way analysis of variance of REN[LA3PTCFF] reporter readouts.

**One Way Analysis of Variance**

**Data source:** REN[LA3PTCFF] in ANOVAs.SNB

**Group Name N Missing Mean Std Dev SEM**

RpS0A 6 0 219521000,000 10243591,792 4181928,837

RpS0B 6 0 151771833,333 8713296,814 3557188,529

RpS1A 6 0 243459166,667 20699271,653 8450442,266

RpS1B 5 0 188621200,000 7283683,182 3257362,144

RpS2 12 0 133613166,667 5974253,150 1724618,332

RpS3 6 0 148648000,000 12127290,019 4950945,418

RpS4A 6 0 128546833,333 4101651,444 1674492,190

RpS4B 6 0 155401500,000 10963041,489 4475642,946

RpS5 6 0 339592000,000 34015248,216 13886666,934

RpS6A 6 0 194130500,000 5352542,004 2185166,123

RpS6B 6 0 136752166,667 3966001,282 1619113,243

RpS7A 5 0 118223000,000 7102187,374 3176194,751

RpS7B 6 0 241620000,000 8844540,983 3610768,736

RpS8A 6 0 228048666,667 8895548,767 3631592,577

RpS9A 12 0 171357750,000 6542478,528 1888650,870

RpS9B 12 0 95505550,000 3736608,267 1078665,894

RpS10A 6 0 111713833,333 3936569,848 1607097,911

RpS10B 6 0 182788333,333 10363501,506 4230881,773

RpS11A 6 0 138534833,333 10358179,443 4228709,050

RpS11B 6 0 133680833,333 3728549,821 1522174,090

RpS12 6 0 172685166,667 6259956,403 2555616,500

RpS13 6 0 185048666,667 6412281,799 2617803,082

RpS14A 6 0 211490500,000 11378281,377 4645163,921

RpS14B 6 0 201813833,333 8363443,535 3414361,526

RpS15 6 0 136015500,000 4244667,184 1732878,121

RpS16A 6 0 172451833,333 7378873,123 3012412,338

RpS16B 6 0 181652000,000 9625218,647 3929479,058

RpS17A 6 0 171161833,333 6970633,125 2845749,057

RpS17B 6 0 207065500,000 7811020,209 3188835,647

RpS18A 5 0 205757400,000 6054290,941 2707561,220

RpS18B 5 0 191679000,000 12902922,770 5770362,484

RpS19A 6 0 206526666,667 23106168,862 9433053,937

RpS19B 6 0 89416766,667 3603855,712 1471267,934

RpS20 6 0 179546333,333 8257259,901 3371012,238

RpS21A 6 0 238979833,333 18679339,361 7625808,361

RpS21B 6 0 236032000,000 16775448,012 6848547,973

RpS22A 6 0 192529166,667 12390695,903 5058480,420

RpS22B 6 0 327183333,333 32146375,022 13123702,647

RpS23A 4 0 154565250,000 1019376,730 509688,365

RpS23B 6 0 159687666,667 11332022,656 4626278,877

RpS24A 6 0 136971333,333 9633091,210 3932693,018

RpS24B 4 0 222332750,000 947622,420 473811,210

RpS25A 6 0 133915666,667 5861004,681 2392745,141

RpS25B 6 0 204214500,000 4515731,646 1843539,725

RpS26B 6 0 224177833,333 17451647,274 7124605,165

RpS27A 6 0 260832166,667 31520527,803 12868201,590

RpS27B 6 0 255817166,667 8345646,156 3407095,776

RpS28A 6 0 140454500,000 6137216,918 2505508,315

RpS28B 6 0 247072333,333 9291155,551 3793098,370

RpS29A 12 0 156539833,333 11006091,023 3177184,807

RpS29B 10 0 104276670,000 12945219,848 4093637,953

RpS30A 6 0 189963500,000 9164432,677 3741363,973

RpS30B 5 0 293168800,000 12735456,674 5695469,370

RpS31 6 0 131397500,000 7360868,556 3005062,004

RpP0 6 0 253967000,000 4995753,917 2039507,996

RpP1A 5 0 250330000,000 3128512,266 1399113,219

RpP1B 6 0 310368166,667 22260315,150 9087735,605

RpP2A 6 0 324556333,333 22414324,149 9150609,516

RpP2B 6 0 263672500,000 8016273,517 3272629,959

RpL1A 6 0 179807500,000 11224926,846 4582557,195

RpL1B 6 0 215669666,667 9352073,859 3817968,165

RpL2A 5 0 225983800,000 15711049,351 7026194,869

RpL2B 5 0 232268200,000 12331879,711 5514984,265

RpL3 6 0 202421500,000 11398871,922 4653569,975

RpL4A 12 0 127619916,667 6875073,850 1984662,869

RpL6A 6 0 213279000,000 9428301,947 3849088,152

RpL6B 6 0 208460666,667 13415980,481 5477051,096

RpL7A 6 0 244761000,000 6554596,067 2675902,639

RpL7B 6 0 263181500,000 11120968,892 4540116,538

RpL8A 6 0 223230333,333 14471080,162 5907793,737

RpL8B 6 0 236177833,333 12797418,262 5224524,128

RpL9A 6 0 199227333,333 8149528,199 3327030,955

RpL10 6 0 231480500,000 10507147,905 4289525,170

RpL11B 6 0 218659500,000 6069828,754 2477997,212

RpL12A 6 0 275731500,000 14031640,485 5728393,240

RpL12B 6 0 127185833,333 2738015,735 1117790,243

RpL13A 6 0 154616666,667 8315568,836 3394816,762

RpL13B 6 0 258382000,000 7390700,670 3017240,914

RpL14A 6 0 224373666,667 12993295,974 5304490,869

RpL15A 6 0 147926833,333 2425589,695 990242,846

RpL15B 6 0 275352833,333 15448798,108 6306945,417

RpL16A 6 0 190535333,333 5777624,552 2358705,346

RpL16B 6 0 275279000,000 26470205,930 10806416,319

RpL17A 6 0 205412166,667 13171452,424 5377222,935

RpL18A 6 0 210437666,667 6533508,787 2667293,793

RpL18B 5 0 193254400,000 9874411,820 4415971,213

RpL19A 6 0 231411833,333 6090946,114 2486618,338

RpL19B 10 0 101338730,000 3990495,011 1261905,323

RpL20A 5 0 307740000,000 10675844,580 4774382,840

RpL20B 6 0 257805833,333 30556729,572 12474732,610

RpL21A 6 0 180168833,333 8590928,341 3507231,809

RpL21B 6 0 317247333,333 13334754,076 5443890,555

RpL22A 6 0 265984333,333 7699961,134 3143495,970

RpL22B 6 0 225784500,000 6482286,441 2646382,358

RpL23A 6 0 231200333,333 7089901,118 2894440,011

RpL23B 6 0 246652000,000 5079178,634 2073565,994

RpL24A 4 0 312731000,000 6665496,281 3332748,141

RpL24B 5 0 238018000,000 17643739,130 7890520,014

RpL25 6 0 328178333,333 16727602,406 6829015,086

RpL26A 6 0 225712000,000 11733350,945 4790120,465

RpL26B 12 0 194452416,667 10628335,698 3068136,238

RpL27A 6 0 242674333,333 10091094,245 4119671,974

RpL27B 3 0 196490666,667 1573583,596 908508,913

RpL28 6 0 199391833,333 5311185,458 2168282,384

RpL29 6 0 132038000,000 7399584,610 3020867,767

RpL30 5 0 165254800,000 9465345,620 4233031,247

RpL31A 6 0 154051000,000 6690321,696 2731312,395

RpL32 6 0 187486000,000 4514031,812 1842845,770

RpL33A 6 0 149735500,000 3610898,655 1474143,203

RpL33B 6 0 206454166,667 9323592,643 3806340,757

RpL34A 6 0 128054833,333 6564917,621 2680116,396

RpL34B 6 0 170254166,667 6531079,893 2666302,201

RpL35A 6 0 173399166,667 9832072,282 4013926,701

RpL35B 6 0 184246666,667 7687547,194 3138428,000

RpL36A 6 0 208004333,333 7140115,004 2914939,744

RpL37A 6 0 174796500,000 7671191,922 3131750,988

RpL37B 6 0 211844666,667 19473153,752 7949881,729

RpL38 6 0 248789666,667 12961772,713 5291621,551

RpL40A 6 0 228977666,667 24697845,328 10082853,133

RpL40B 6 0 241392166,667 12896619,827 5265022,997

RpL41A 6 0 288332833,333 12931063,489 5279084,563

RpL41B 6 0 257054000,000 7527076,803 3072916,237

RpL42A 0 0 194505166,667 19384502,505 (+inf)

RpL43B 6 0 245042333,333 15746156,568 6428341,500

Grand Mean 124 0 204840798,925 54793269,107 4920580,823

**Source of Variation DF SS MS F P**

Between Groups 124 2,385E+018 1,923E+016 31,777 <0,001

Residual 760 4,600E+017 6,053E+014

Total 884 2,845E+018

The differences in the mean values among the treatment groups are greater than would be expected by chance; there is a statistically significant difference (P = <0,001).

Power of performed test with alpha = 0,050: 1,000

Multiple Comparisons versus Control Group (Holm-Sidak method):

Overall significance level = 0,05

Comparisons for factor:

**Comparison Diff of Means t Unadjusted P Critical Level Significant?**

Grand Mean vs. RpS9B 109335248,925 14,700 3,093E-043 0,000 Yes

Grand Mean vs. RpS5 134751201,075 13,103 1,631E-035 0,000 Yes

Grand Mean vs. RpL19B 103502068,925 12,798 4,243E-034 0,000 Yes

Grand Mean vs. RpS29B 100564128,925 12,434 1,921E-032 0,000 Yes

Grand Mean vs. RpL25 123337534,408 11,993 1,790E-030 0,000 Yes

Grand Mean vs. RpS22B 122342534,408 11,896 4,768E-030 0,000 Yes

Grand Mean vs. RpP2A 119715534,408 11,641 6,173E-029 0,000 Yes

Grand Mean vs. RpS19B 115424032,258 11,224 3,729E-027 0,000 Yes

Grand Mean vs. RpL21B 112406534,408 10,930 6,260E-026 0,000 Yes

Grand Mean vs. RpL4A 77220882,258 10,382 1,052E-023 0,000 Yes

Grand Mean vs. RpP1B 105527367,742 10,261 3,174E-023 0,000 Yes

Grand Mean vs. RpS2 71227632,258 9,576 1,379E-020 0,000 Yes

Grand Mean vs. RpL20A 102899201,075 9,169 4,380E-019 0,000 Yes

Grand Mean vs. RpS10A 93126965,592 9,056 1,128E-018 0,000 Yes

Grand Mean vs. RpL24A 107890201,075 8,633 3,494E-017 0,000 Yes

Grand Mean vs. RpL41A 83492034,408 8,119 1,898E-015 0,000 Yes

Grand Mean vs. RpS30B 88328001,075 7,871 1,213E-014 0,000 Yes

Grand Mean vs. RpS7A 86617798,925 7,718 3,709E-014 0,000 Yes

Grand Mean vs. RpL12B 77654965,592 7,551 1,240E-013 0,000 Yes

Grand Mean vs. RpL34A 76785965,592 7,467 2,261E-013 0,000 Yes

Grand Mean vs. RpS4A 76293965,592 7,419 3,169E-013 0,000 Yes

Grand Mean vs. RpS31 73443298,925 7,141 2,163E-012 0,001 Yes

Grand Mean vs. RpL29 72802798,925 7,079 3,302E-012 0,001 Yes

Grand Mean vs. RpS11B 71159965,592 6,919 9,636E-012 0,001 Yes

Grand Mean vs. RpS25A 70925132,258 6,897 1,121E-011 0,001 Yes

Grand Mean vs. RpL12A 70890701,075 6,893 1,146E-011 0,001 Yes

Grand Mean vs. RpL15B 70512034,408 6,856 1,462E-011 0,001 Yes

Grand Mean vs. RpL16B 70438201,075 6,849 1,533E-011 0,001 Yes

Grand Mean vs. RpS15 68825298,925 6,692 4,261E-011 0,001 Yes

Grand Mean vs. RpS6B 68088632,258 6,621 6,751E-011 0,001 Yes

Grand Mean vs. RpS24A 67869465,592 6,600 7,736E-011 0,001 Yes

Grand Mean vs. RpS29A 48300965,592 6,494 0,000000000151 0,001 Yes

Grand Mean vs. RpS11A 66305965,592 6,447 0,000000000202 0,001 Yes

Grand Mean vs. RpS28A 64386298,925 6,261 0,000000000640 0,001 Yes

Grand Mean vs. RpL22A 61143534,408 5,945 0,00000000420 0,001 Yes

Grand Mean vs. RpP2B 58831701,075 5,721 0,0000000153 0,001 Yes

Grand Mean vs. RpL7B 58340701,075 5,673 0,0000000200 0,001 Yes

Grand Mean vs. RpL15A 56913965,592 5,534 0,0000000431 0,001 Yes

Grand Mean vs. RpS3 56192798,925 5,464 0,0000000631 0,001 Yes

Grand Mean vs. RpS27A 55991367,742 5,445 0,0000000702 0,001 Yes

Grand Mean vs. RpL33A 55105298,925 5,358 0,000000111 0,001 Yes

Grand Mean vs. RpL13B 53541201,075 5,206 0,000000248 0,001 Yes

Grand Mean vs. RpS0B 53068965,592 5,160 0,000000315 0,001 Yes

Grand Mean vs. RpL20B 52965034,408 5,150 0,000000332 0,001 Yes

Grand Mean vs. RpL41B 52213201,075 5,077 0,000000482 0,001 Yes

Grand Mean vs. RpS27B 50976367,742 4,957 0,000000884 0,001 Yes

Grand Mean vs. RpL31A 50789798,925 4,939 0,000000967 0,001 Yes

Grand Mean vs. RpL13A 50224132,258 4,884 0,00000127 0,001 Yes

Grand Mean vs. RpS4B 49439298,925 4,807 0,00000184 0,001 Yes

Grand Mean vs. RpP0 49126201,075 4,777 0,00000214 0,001 Yes

Grand Mean vs. RpS9A 33483048,925 4,502 0,00000780 0,001 Yes

Grand Mean vs. RpS23B 45153132,258 4,391 0,0000129 0,001 Yes

Grand Mean vs. RpL38 43948867,742 4,274 0,0000217 0,001 Yes

Grand Mean vs. RpS28B 42231534,408 4,107 0,0000445 0,001 Yes

Grand Mean vs. RpL23B 41811201,075 4,066 0,0000529 0,001 Yes

Grand Mean vs. RpP1A 45489201,075 4,054 0,0000557 0,001 Yes

Grand Mean vs. RpS23A 50275548,925 4,023 0,0000633 0,001 Yes

Grand Mean vs. RpL43B 40201534,408 3,909 0,000101 0,001 Yes

Grand Mean vs. RpL7A 39920201,075 3,882 0,000113 0,001 Yes

Grand Mean vs. RpS1A 38618367,742 3,755 0,000186 0,001 Yes

Grand Mean vs. RpL27A 37833534,408 3,679 0,000251 0,001 Yes

Grand Mean vs. RpS7B 36779201,075 3,576 0,000370 0,001 Yes

Grand Mean vs. RpL40B 36551367,742 3,554 0,000403 0,001 Yes

Grand Mean vs. RpL30 39585998,925 3,527 0,000445 0,001 Yes

Grand Mean vs. RpL34B 34586632,258 3,363 0,000809 0,001 Yes

Grand Mean vs. RpS21A 34139034,408 3,320 0,000944 0,001 No

Grand Mean vs. RpS17A 33678965,592 3,275 0,00110 0,001 No

Grand Mean vs. RpS16A 32388965,592 3,149 0,00170 0,001 No

Grand Mean vs. RpS12 32155632,258 3,127 0,00183 0,001 No

Grand Mean vs. RpL35A 31441632,258 3,057 0,00231 0,001 No

Grand Mean vs. RpL8B 31337034,408 3,047 0,00239 0,001 No

Grand Mean vs. RpS21B 31191201,075 3,033 0,00250 0,001 No

Grand Mean vs. RpL24B 33177201,075 2,956 0,00321 0,001 No

Grand Mean vs. RpL37A 30044298,925 2,921 0,00359 0,001 No

Grand Mean vs. RpL10 26639701,075 2,590 0,00977 0,001 No

Grand Mean vs. RpL19A 26571034,408 2,584 0,00996 0,001 No

Grand Mean vs. RpL23A 26359534,408 2,563 0,0106 0,001 No

Grand Mean vs. RpS20 25294465,592 2,460 0,0141 0,001 No

Grand Mean vs. RpL2B 27427401,075 2,444 0,0148 0,001 No

Grand Mean vs. RpL1A 25033298,925 2,434 0,0152 0,001 No

Grand Mean vs. RpL21A 24671965,592 2,399 0,0167 0,001 No

Grand Mean vs. RpL40A 24136867,742 2,347 0,0192 0,001 No

Grand Mean vs. RpS8A 23207867,742 2,257 0,0243 0,001 No

Grand Mean vs. RpS16B 23188798,925 2,255 0,0244 0,001 No

Grand Mean vs. RpS10B 22052465,592 2,144 0,0323 0,001 No

Grand Mean vs. RpL22B 20943701,075 2,037 0,0420 0,001 No

Grand Mean vs. RpL26A 20871201,075 2,029 0,0428 0,001 No

Grand Mean vs. RpL35B 20594132,258 2,003 0,0456 0,001 No

Grand Mean vs. RpS13 19792132,258 1,925 0,0547 0,001 No

Grand Mean vs. RpL14A 19532867,742 1,899 0,0579 0,002 No

Grand Mean vs. RpL2A 21143001,075 1,884 0,0599 0,002 No

Grand Mean vs. RpS26B 19337034,408 1,880 0,0604 0,002 No

Grand Mean vs. RpL8A 18389534,408 1,788 0,0741 0,002 No

Grand Mean vs. RpL32 17354798,925 1,688 0,0919 0,002 No

Grand Mean vs. RpS30A 14877298,925 1,447 0,148 0,002 No

Grand Mean vs. RpS1B 16219598,925 1,445 0,149 0,002 No

Grand Mean vs. RpS0A 14680201,075 1,427 0,154 0,002 No

Grand Mean vs. RpS24B 17491951,075 1,400 0,162 0,002 No

Grand Mean vs. RpL26B 10388382,258 1,397 0,163 0,002 No

Grand Mean vs. RpL16A 14305465,592 1,391 0,165 0,002 No

Grand Mean vs. RpL11B 13818701,075 1,344 0,179 0,002 No

Grand Mean vs. RpS22A 12311632,258 1,197 0,232 0,002 No

Grand Mean vs. RpS18B 13161798,925 1,173 0,241 0,002 No

Grand Mean vs. RpL1B 10828867,742 1,053 0,293 0,003 No

Grand Mean vs. RpS6A 10710298,925 1,041 0,298 0,003 No

Grand Mean vs. RpL18B 11586398,925 1,032 0,302 0,003 No

Grand Mean vs. RpL6A 8438201,075 0,821 0,412 0,003 No

Grand Mean vs. RpL37B 7003867,742 0,681 0,496 0,003 No

Grand Mean vs. RpS14A 6649701,075 0,647 0,518 0,003 No

Grand Mean vs. RpL27B 8350132,258 0,581 0,561 0,004 No

Grand Mean vs. RpL9A 5613465,592 0,546 0,585 0,004 No

Grand Mean vs. RpL18A 5596867,742 0,544 0,586 0,004 No

Grand Mean vs. RpL28 5448965,592 0,530 0,596 0,005 No

Grand Mean vs. RpL6B 3619867,742 0,352 0,725 0,005 No

Grand Mean vs. RpL36A 3163534,408 0,308 0,758 0,006 No

Grand Mean vs. RpS14B 3026965,592 0,294 0,769 0,006 No

Grand Mean vs. RpL3 2419298,925 0,235 0,814 0,007 No

Grand Mean vs. RpS17B 2224701,075 0,216 0,829 0,009 No

Grand Mean vs. RpS19A 1685867,742 0,164 0,870 0,010 No

Grand Mean vs. RpL33B 1613367,742 0,157 0,875 0,013 No

Grand Mean vs. RpS18A 916601,075 0,0817 0,935 0,017 No

Grand Mean vs. RpS25B 626298,925 0,0609 0,951 0,025 No

Grand Mean vs. RpL17A 571367,742 0,0556 0,956 0,050 No
